# Flexible Selenium Nanowires with Tuneable Electronic Bandgaps

**DOI:** 10.1002/adma.202501821

**Published:** 2025-05-21

**Authors:** William J. Cull, Quentin M. Ramasse, Johannes Biskupek, Graham A. Rance, Ian Cardillo‐Zallo, Benjamin L. Weare, Michael W. Fay, R. Roy Whitney, Lyndsey R. Scammell, Jesum Alves Fernandes, Ute Kaiser, Amalia Patanè, Andrei N. Khlobystov

**Affiliations:** ^1^ School of Chemistry University of Nottingham Nottingham NG7 2RD UK; ^2^ SuperSTEM Laboratory SciTech Daresbury Campus Daresbury WA4 4AD UK; ^3^ School of Chemical and Process Engineering and School of Physics and Astronomy University of Leeds Leeds LS2 9JT UK; ^4^ Electron Microscopy Group of Materials Science Central Facility for Electron Microscopy Ulm University 89081 Ulm Germany; ^5^ Nanoscale and Microscale Research Centre University of Nottingham Nottingham NG7 2QL UK; ^6^ BNNT LLC Newport News VA 23606 USA; ^7^ School of Physics University of Nottingham Nottingham NG7 2RD UK

**Keywords:** bandgap, boron nitride nanotubes, carbon nanotubes, nanowires, phase change, selenium, transmission electron microscopy

## Abstract

Manipulating semiconductor properties without altering their chemical composition holds promise for electronic and optical materials. However, linking atomic positions in nanomaterials to their functional properties is challenging due to their polydispersity. This study utilizes nano test tubes to uncover distinct phases of selenium, an elemental semiconductor, demonstrating a remarkable structural plasticity between 0.4 and 3.0 nm. These structures are correlated with their electronic bandgaps, ranging from 2.2 to 2.5 eV, using ultra‐low‐loss electron energy loss spectroscopy and aberration‐corrected scanning transmission electron microscopy for individual nanowires in boron nitride nanotubes (BNNT). Notably, the variation in bandgaps diverges from that of bulk selenium and is non‐monotonic on the host‐nanotube diameter, indicating that conformational distortions in selenium chains begin counteracting quantum confinement effects at sub‐nm scales. A 1D phase diagram predicting selenium's atomic structure based on nanotube diameter, regardless of the chemistry of the host nanotube is developed, which can be BNNT or carbon nanotubes. Phase changes in selenium nanowires are imaged in real‐time by transmission electron microscopy using BNNT as a test tube with an adjustable diameter. These nanoscale findings pave the way for the development of advanced miniature tuneable and flexible electronic components, including transistors, optical sensors, and photovoltaics.

## Graphical abstract

Nanotubes serve as effective test tubes for selenium nanowires, as their diameter precisely controls the width of the nanowires and the Se─Se bonding, resulting in a variety of different structures. An electron beam can be used to modify the nanotubes, allowing real‐time observation of the nanowire extrusion process. Additionally, optically transparent boron nitride nanotubes enable direct bandgap measurements for individual nanowires, revealing the interplay between various phenomena occurring at the nanoscale.

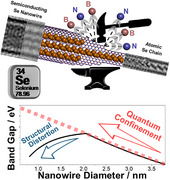

## 1 Introduction

Following its discovery in 1817,^[^ ^1^ ^]^ the element selenium (Se) has found a multitude of applications across the physical sciences. Experiments performed on Se in the 1870s led to the discovery of photoconductivity,^[^ ^2^, ^3^ ^]^ the realisation of the photovoltaic effect in solid semiconductors^[^ ^4^ ^]^ and its use as the photoactive component of the first solar cells.^[^ ^5^ ^]^ Ample research into the production and optimisation of Se semiconducting materials of specific allotropes has been performed in recent years due to its phase‐ and dimensionality‐dependent electronic and optical properties.^[^ ^6^, ^7^, ^8^ ^]^ When confined to the nanoscale Se can exist in various form‐factors,^[^ ^9^ ^]^ including nanowires (NWs),^[^ ^10^ ^]^ nanorods,^[^ ^11^ ^]^ nanosheets^[^ ^12^ ^]^ and nanoparticles.^[^ ^13^ ^]^ These nanomaterials have a wide range of potential applications, including components in nanoscale solar cells^[^ ^12^, ^14^ ^]^ and energy storage materials.^[^ ^15^ ^]^

A highly effective method for controlling dimensionally, and hence the functional properties of materials, is confinement in carbon nanotubes (CNTs). The cylindrical internal cavity of CNTs allows for the templated growth of high aspect ratio functional nanomaterials, while also allowing for atomic structures of materials to be studied by transmission electron microscopy (TEM).^[^ ^16^, ^17^, ^18^ ^]^ A wide range of semiconducting materials, including SnSe,^[^ ^19^ ^]^ In_2_Se_3_^[^ ^20^ ^]^ and CsPbBr_3_^[^ ^21^ ^]^ have been nanoconfined in this manner, alongside the elemental chalcogens S,^[^ ^22^ ^]^ Se^[^ ^23^ ^]^ and Te.^[^ ^24^ ^]^ Se was first encapsulated inside multi‐walled carbon nanotubes (MWCNTs) in 1996,^[^ ^25^ ^]^ but has since been encapsulated inside CNTs of a range of diameters.^[^ ^26^, ^27^ ^]^ Fujimori et al. utilized small‐diameter double‐walled carbon nanotubes (DWCNTs) to encapsulate single‐ and double‐helix Se chains measuring only 0.7 nm in diameter.^[^ ^23^ ^]^ As the diameter of the encapsulating CNT becomes larger, other conformations of Se are templated, taking the form of multiple trigonal Se (t‐Se) chains^[^ ^28^ ^]^ or ‘liquid‐like’ t‐Se with a visible meniscus.^[^ ^26^ ^]^ However, it is not possible to establish a connection between the structure of semiconducting NWs and their functional properties in CNTs, as any optical or electronic measurements are strongly dominated by the electronic structure of the host nanotube, cloaking the semiconductor wire within.

Boron nitride nanotubes (BNNTs) are isostructural to CNTs but have vastly different properties due to their differences in elemental composition.^[^ ^29^ ^]^ While CNTs are highly absorbing narrow‐gap semiconductors or metals, BNNTs are optically transparent insulators, making the latter ideal hosts for electronically, optically and magnetically active materials. However, examples of NW growth within BNNTs are rare^[^ ^30^ ^]^ and include graphene nanoribbons,^[^ ^31^, ^32^ ^]^ Fe NWs,^[^ ^33^ ^]^ KCl, KBr and KI NWs^[^ ^34^ ^]^ and nested single‐walled carbon nanotubes (SWCNTs).^[^ ^35^ ^]^ Recent studies have shown a clear advantage of BNNTs over CNTs as effective hosts for extending the fluorescence of organic dyes^[^ ^36^ ^]^ and allowing access to the electronic^[^ ^37^ ^]^ and optical^[^ ^38^ ^]^ properties of encapsulated semiconductors.

In this article, we utilize the power of nano test tubes to grow nanowires of an elementary semiconductor, selenium, at different degrees of confinement. We demonstrate that this material exhibits remarkable structural plasticity, existing in a wide variety of forms, ranging from linear atomic chains to trigonal helices to nanosized nanowires. The controlled synthesis of these structures unlocks the full potential of this semiconductor. Our toolbox includes two types of nano test tubes – one opaque and conducting and the other transparent and insulating – CNT and BNNT, respectively, and the electron beam that is utilized in three ways – to image atomic positions, record spectra at the single‐particle level, and manipulate the nanowires. Our approach uncovers the intricate relationship between the degree of confinement and the chemistry, structure and bonding of selenium nanowires, leading to tailorable optical properties.

## 2 Results and Discussion

### 2.1 Growth of Selenium Nanowires Inside Carbon and Boron Nitride Nanotubes

**Figure 1:**
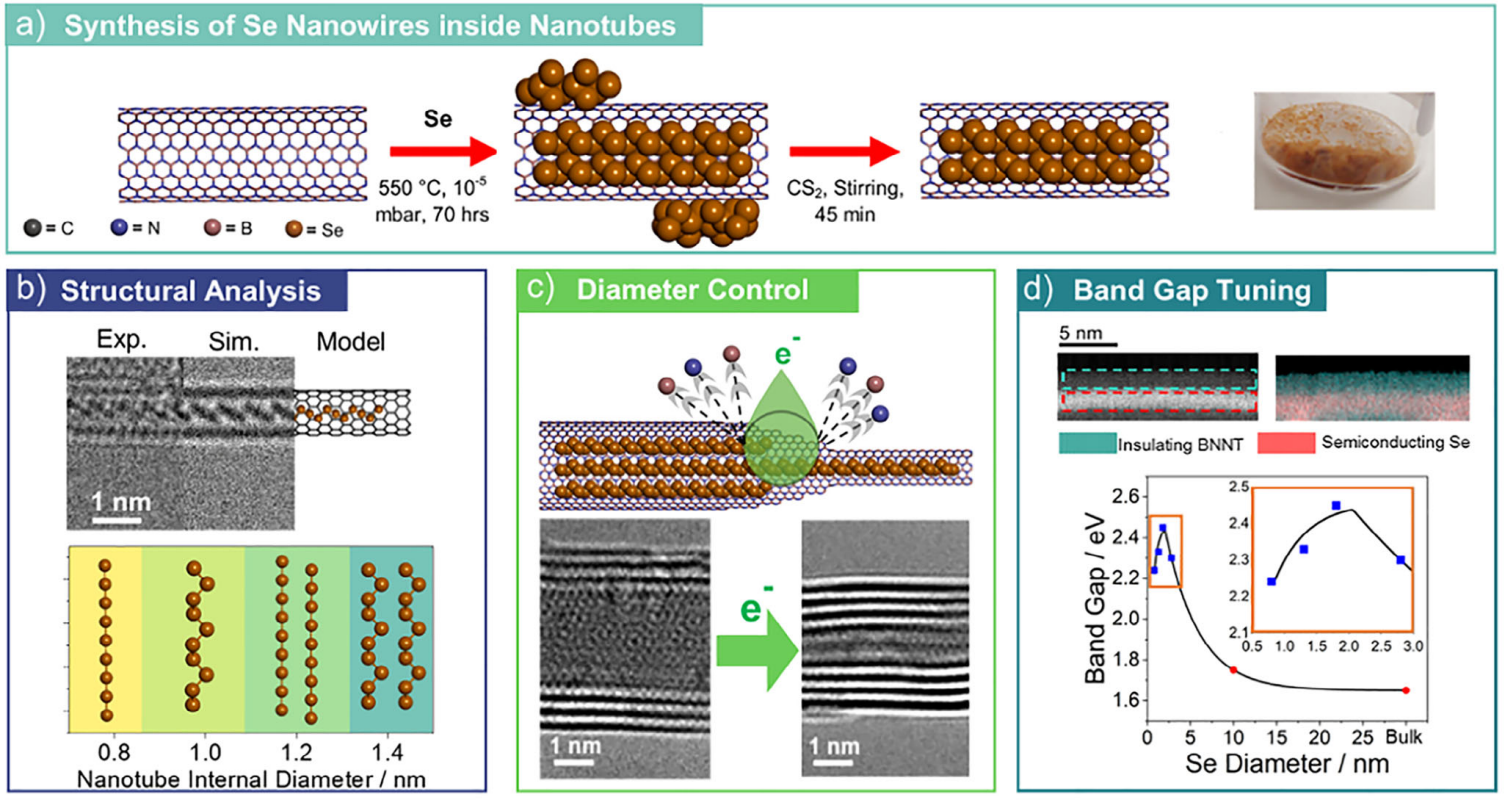
Sub‐nanometre diameter control and bandgap analysis of individual selenium nanowires through electron microscopy: a) Schematic depicting the two‐step procedure used to encapsulate Se inside BNNTs, the same procedure as used for CNTs and photograph of solid Se@BNNT, b) through AC‐HRTEM imaging the dependence of NT diameter on the structure and bonding of Se NWs is established, allowing for a 1D phase diagram to be produced, c) Through electron beam irradiation of Se@BNNT the diameter of encapsulated Se NWs can be controlled with sub‐nm precision, down to linear single‐atom chains, d) AC‐STEM EELS analysis reveals the effect of diameter on the bandgap of individual Se NWs.

In this study, five different NTs (technical specifications in Table S1), one BNNT and four CNTs of a range of diameters (Figures S1 and S2; Table S2, Supporting Information), are utilized as containers for the growth of Se NWs. A sublimation filling method was utilized (Methods, **Figure** **1**a) to prepare both Se inside CNTs (Se@CNT) and Se inside BNNTs (Se@BNNT). The same experimental conditions for Se NW growth were used for all varieties of NTs, allowing us to focus on the effect of NT diameter on the structure of the templated NW. Due to differences in the optical transparency of the nanocontainer, CNTs remain a black powder, whereas BNNTs turn from white to orange. For all NTs, Raman spectroscopy was utilized to confirm the nanoconfinement of Se (Figures S3 and S4; Tables S3 and S4, Supporting Information).

### 2.2 The Effect of NT Diameter on the Structure and Bonding of Encapsulated Selenium

**Figure 2:**
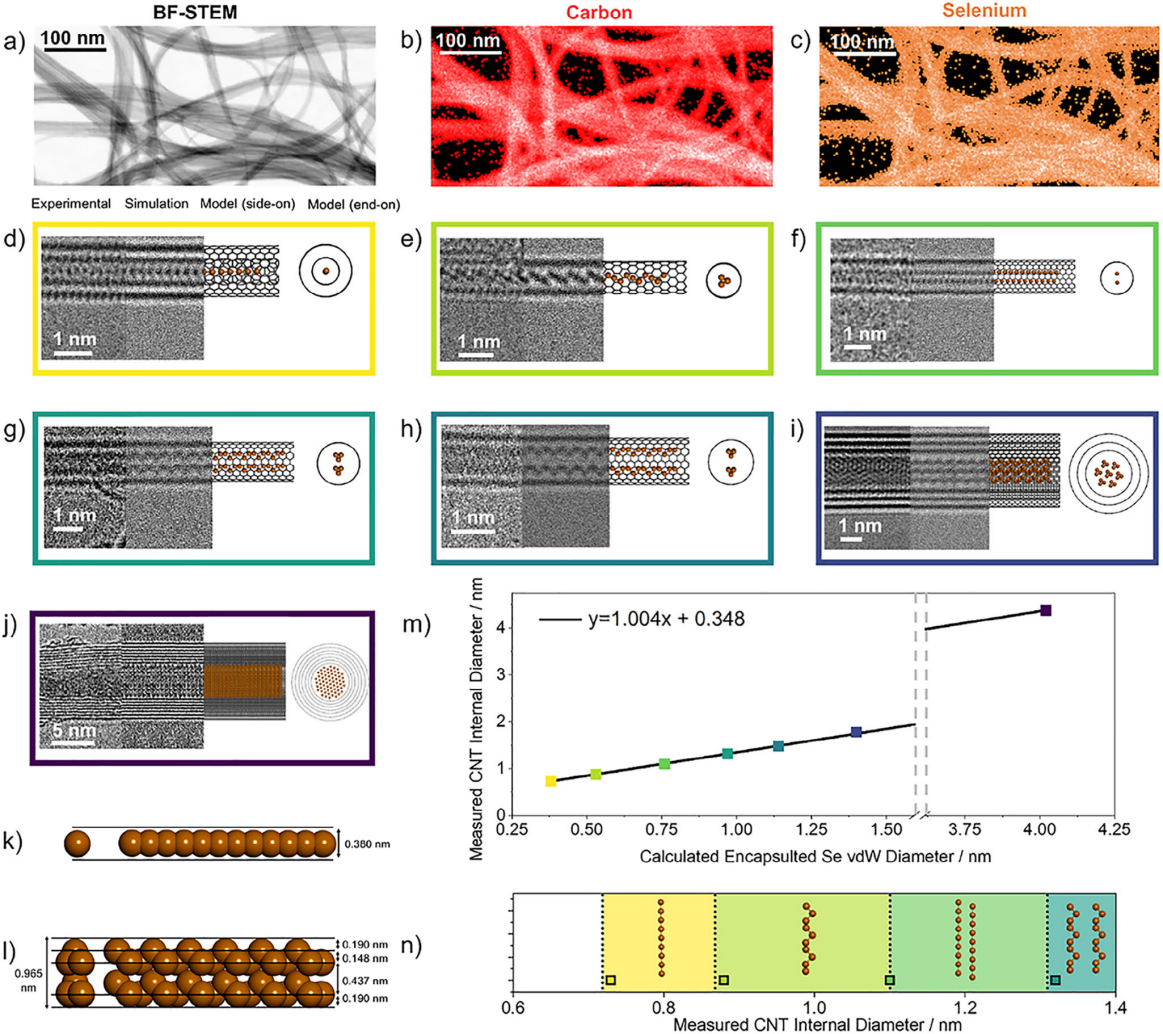
TEM Analysis of the Selenium NWs inside CNTs: a) 120 kV bright‐field (BF) STEM image of Se@SWCNT bundles, b,c) carbon and selenium EDX maps of the region shown in a), respectively, d–j) composite images containing experimental AC‐TEM image (left), simulated TEM image (centre left), side‐on molecular model (centre right) and end‐on molecular model (right) of various diameter Se@CNT systems, k,l) space‐filling molecular model a l‐Se chain and of two t‐Se chains, respectively, Se atoms in orange, m) graphical representation of the linear relationship between measured CNT internal diameter and simulated Se vdW diameter (Adj. R^2^ = 0.9998), n) 1D phase diagram showing the effect of CNT diameter on the conformation of encapsulated Se. Coloured squares correspond to conformations seen experimentally, coloured regions correspond to the dimensions of CNT that the specified conformations are expected to exist in and black dotted lines designate the minimum CNT internal diameters that can accommodate particular phases of Se, detailed further in Table S6 (Supporting Information).

Energy dispersive X‐ray (EDX) analysis (Figure S5, Supporting Information) was used to investigate the four Se@CNT systems, revealing peaks corresponding to Se (Lα). Scanning transmission electron microscopy‐energy dispersive X‐ray (STEM‐EDX) analysis confirmed both uniform Se distribution within 20–50 nm bundles of CNTs (**Figure** **2**a–c) and Se encapsulation by individual NTs (Figure S6, Supporting Information).

High‐resolution TEM (HRTEM) and aberration‐correct HRTEM (AC‐HRTEM) imaging, complemented by TEM image simulations, were utilized to determine the effect of CNT internal diameter on the structure and bonding of encapsulated Se NWs. The smallest diameter CNT (Figure 2d), measuring 0.73 nm in diameter, encapsulates a linear atomic chain of Se (l‐Se, structure in Figure 2k). A TEM image simulation of a l‐Se inside a CNT of the same diameter (Figure S7, Supporting Information) correlates well with experimental images, with interatomic distances of 0.23 nm in the experimental image and 0.24 nm in the simulated image. The longest single chain of l‐Se imaged by TEM measures at least 28 nm (Figure S8, Supporting Information). While the formation of l‐Se chains inside cancrinite nanochannels has been confirmed previously by Raman analysis,^[^ ^39^ ^]^ their existence has not been proven directly by imaging. The structure we observed for l‐Se in very narrow nanotubes is consistent with other chalcogens, as both Te^[^ ^24^ ^]^ and S^[^ ^22^ ^]^ have also been shown to adopt linear atomic chain conformations under the effects of extreme confinement.

Increasing the diameter of the CNT to 0.88 nm (Figure 2e) gives rise to high translational and rotational mobility of encapsulated Se chains (Figure S9, Supporting Information), which can cause difficulty in obtaining atomically resolved TEM images of the encapsulated species. To counteract this, encapsulated Se found near defective areas of CNTs (green arrow in Figure S10a, Supporting Information) were imaged. These defective regions reduced the translational mobility of Se, allowing for atomic resolution during AC‐HRTEM imaging. However, the rotational freedom of Se NWs remains on the timescale of image capture (≈ 1 fps), which allows us to inspect different orientations of the same NW. A simulated rotational tableau was compared to experimental images of a suspected single‐chain Se conformation, enabling the determination of the atomic structure as a single chain of t‐Se (Figure 2e; Figures S11 and S12, Supporting Information).^[^ ^40^ ^]^

When the diameter of the CNT is increased further, to 1.10 nm, encapsulated Se assumes a conformation of two co‐linear l‐Se chains (Figure 2f). The distance between the two chains was experimentally measured as 0.38 nm, which corresponds to double the van der Waals radius of Se (0.19 nm).^[^ ^41^ ^]^ Additionally, Se atomic spacings along a chain are measured as 0.23 nm in both experimental and simulated images (Figure S13, Supporting Information). As highlighted by the yellow square in Figure S13c (Supporting Information), the two co‐linear Se atomic chains appear to have a staggered conformation, likely driven by the optimisation of van der Waals interactions. In some cases, the two co‐linear l‐Se chains were observed to twist along the axis of the CNT (Figure S14, Supporting Information), leading to varied interchain separation distances in projection. Co‐linear atomic chains of Te inside CNTs have previously been reported by Qin et al., requiring encapsulation by a CNT of 1.2 nm in diameter.^[^ ^42^ ^]^ These co‐linear atomic chains of Se may have a similar appearance when in motion to the double helices previously reported by Fujimori et al.^[^ ^23^ ^]^ These authors showed that the fast motion of Se chains prevented detailed atomic imaging of their structures. In this work, we have focussed our investigation on stationary structures, which allows for an exact determination of Se atomic positions.

Once the diameter of the CNT becomes larger than 1.32 nm, encapsulation of two chains of t‐Se (Figure 2l) becomes possible. Due to van der Waals constraints, two chains of t‐Se is the largest diameter Se‐based structure that can be encapsulated in a CNT of this size. The internal diameter of the host CNT determines the spacing between the two chains of t‐Se (Figure 2g,h; Figure S15, Supporting Information), due to favorable interactions between the Se chains and the CNT walls,^[^ ^43^ ^]^ increasing the average inter‐chain separation as CNT diameter increases. Similar to the aforementioned co‐linear chains of l‐Se, in some instances, two chains of t‐Se were observed to twist along the axis of the CNT, resulting in varied interchain separation distances in projection (Figure S16, Supporting Information). Further increases in CNT diameter allow for the encapsulation of multiple t‐Se chains (Figure 2i), identified by comparing the inter‐chain spacing in simulated and experimental images (Figure S17, Supporting Information). When CNTs of this diameter encapsulate Se NWs, they still possess high translational and rotational mobility (Figure S18, Supporting Information). As soon as the diameter of a CNT becomes large enough to allow for two chains of l‐Se or t‐Se to be encapsulated, the encapsulated species becomes significantly less crystalline and more similar to that of amorphous selenium.^[^ ^44^ ^]^ The high mobility (Figure S9, Supporting Information) and irregular twist axes (Figure S14, Supporting Information) of these encapsulated nanowires result in long‐range disorder; however, short‐range order is observed in interatomic (Figure S13, Supporting Information) and interchain (Figure S15, Supporting Information) spacings, which are similar to literature values for crystalline structures. This is analogous to the assessment of amorphous selenium made by Laude and Fitton,^[^ ^45^ ^]^ who describe the aforementioned material as something more similar to a disordered crystal than a truly amorphous solid.^[^ ^46^ ^]^

TEM analysis of Se@CNT systems where the CNT internal diameter measures above 3 nm (Figure 2j) revealed encapsulated Se with menisci, as previously seen by Dutta et al.^[^ ^26^ ^]^ In two MWCNTs with different internal diameters, both convex and concave menisci are seen (Figure S19, Supporting Information), indicating that effective wetting of the internal surface of the larger diameter CNTs with Se, and thus the surface tension of Se is highly dependent on the diameter of the CNTs.^[^ ^47^ ^]^ Unlike when encapsulated by smaller diameter CNTs, no atomic contrast corresponding to t‐Se was observed in CNTs of this size. TEM simulations of large bundles of t‐Se (Figure S21, Supporting Information) show that a loss of defined contrast is seen when the structures are rotated around the CNT axis. Furthermore, these wide Se NWs exhibited translational motion, moving within the internal cavity of the CNTs during imaging (Figure S20, Supporting Information). These two factors explain the lack of spacing corresponding to t‐Se seen in the experimental images in CNTs of this diameter.

Our imaging data shows a clear linear relationship between CNT internal diameter and the diameter of the encapsulated Se NW (Figure 2m, data used shown in Table S5, Supporting Information), revealing that Se inside CNT fills all available space regardless of CNT internal diameter. The structural flexibility of Se chains allows them to switch between linear and trigonal geometries, maximising interaction with the CNT due to the nature of the Se─Se bond angles, which can be easily distorted. This is different to typical ionic and covalent materials encapsulated in CNTs, where the bond angles and stoichiometry constrain structural flexibility in confinement. Figure 2n presents a 1D phase diagram for Se, enabling the prediction and thus control of the nanoconfined Se conformation based on the dimensions of the host NT (data used are shown in Table S6, Supporting Information).

### 2.3 Real‐Time Phase Change Dynamics in Se NW Driven by In Situ Control of Nanotube Diameter

**Figure 3:**
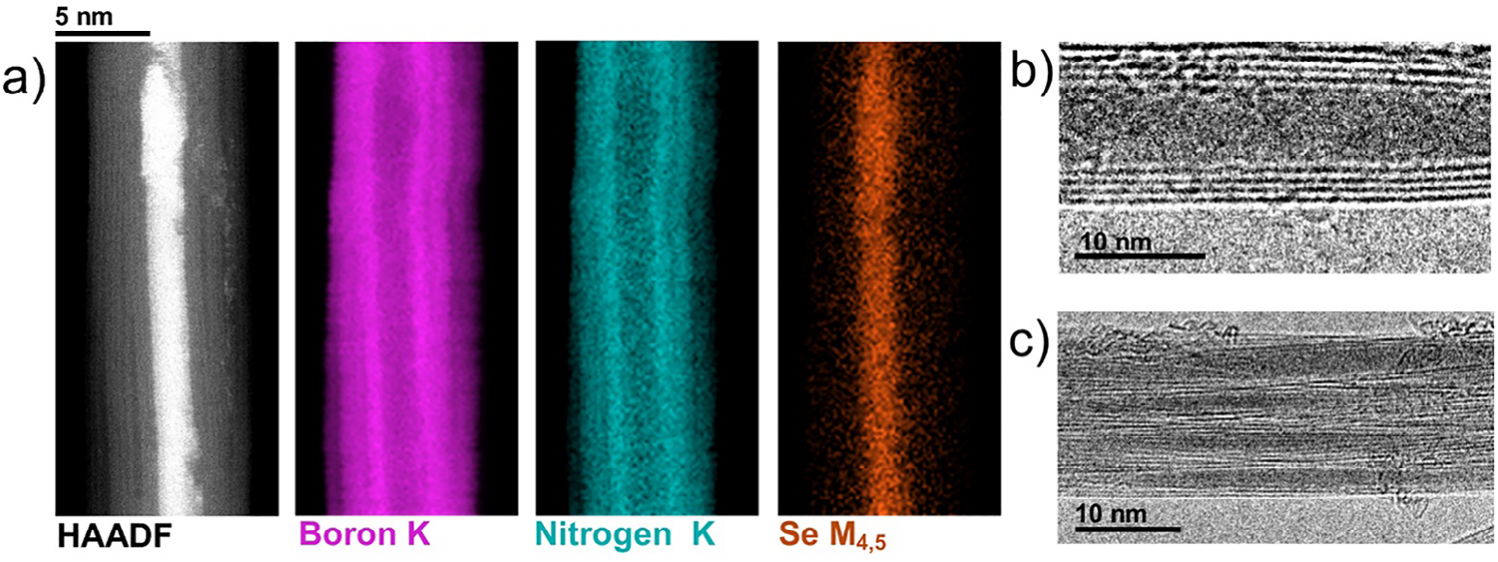
Nanoscale Characterisation of Se@BNNT a) 60 kV HAADF image and STEM‐EELS maps of Se@BNNT showing the encapsulation of Se, b,c) 200 kV TEM images of a single BNNT filled with Se and a bundle of Se‐filled BNNTs, respectively.

Electron microscopy of Se@BNNT revealed that the procedure detailed in Figure 1a clearly results in the successful encapsulation of Se by BNNTs. High‐angle annular dark‐field (HAADF) AC‐STEM imaging of a single BNNT filled with Se (**Figure** **3**a), alongside B (K), N (K) and Se (M_4,5_) edge EELS maps highlights the presence of Se inside the internal cavity of the BNNT. HRTEM imaging (Figure 3b,c) and EDX analysis (Figure S22, Supporting Information) of bundles of Se@BNNT also confirmed the presence of encapsulated Se. Similar to the results seen in TEM analysis of Se@CNT, encapsulated Se was found to be highly mobile inside BNNTs (Figure S23, Supporting Information), with larger diameter BNNTs showing little evidence of inter‐chain spacings corresponding to t‐Se. However, following continued 200 kV electron beam irradiation of Se@BNNT during TEM imaging, lines of contrast with a similar spacing to that of the interchain spacing of bulk t‐Se emerged (Figure S24, Supporting Information).

**Figure 4:**
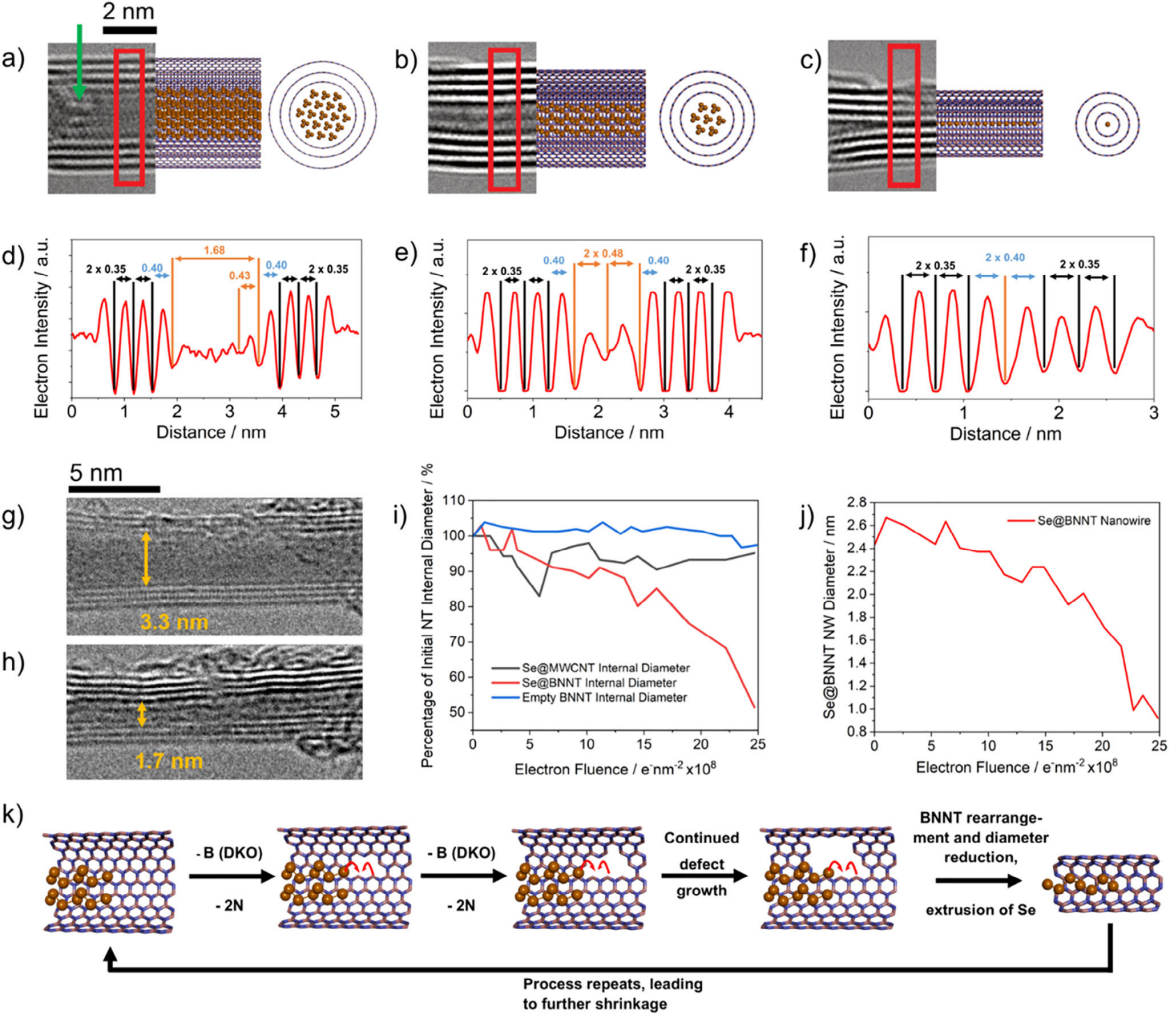
Controlling the diameter of Se NWs in‐situ by electron beam irradiation a), b) and c) composited images containing 80 kV AC‐TEM images (left) side on molecular models (centre) and end on molecular models (right) of the same section of Se@BNNT during continued e‐beam irradiation (see Video S1, Supporting Information), d,e,f) line profile analysis of the red boxes shown in a), b) and c), respectively, g) and h) 200 kV TEM images of Se@BNNT before and after an electron fluence of 25 × 10^8^ e^−^nm^−2^ has been administered, i) demonstration of the percentage change in internal NT diameter during continued e‐beam irradiation for Se@BNNT, Se@MWCNT and an empty BNNT, j) change in the measured diameter of the internally encapsulated Se NW shown in g) and h) during continued e‐beam irradiation, k) schematic showing the process that causes shrinkage of BNNTs and extrusion of Se following e‐beam irradiation.

To further understand the electron‐beam‐induced reaction (Figure S24, Supporting Information), Se@BNNT was imaged under controlled irradiation with an 80 kV electron beam using AC‐HRTEM. A single BNNT filled with Se under a flux of 1.2 × 10^8^ e^−^nm^−2^ s^−1^ (Video S1; Figure S25, Supporting Information) showed the formation of defects in the sidewall of the BNNT, indicated by the green arrow in **Figure** **4**a. Following further irradiation, these defects, caused by direct knock‐on (DKO) damage, grow. As shown previously (albeit using empty BNNTs) by Celik‐Aktas et al.^[^ ^48^ ^]^ and Cheng et al.^[^ ^49^ ^]^ the removal of B atoms in this manner can also lead to the elimination of N, resulting in the irradiated BNNT losing mass. This process is known to occur much more quickly for the inner BNNT of a multiwall BNNT, due to its higher curvature and, therefore, strain.^[^ ^48^, ^49^ ^]^ Our imaging under these conditions over 400 s shows the formation of lines of contrast corresponding to chains of Se, which are initially found in areas adjacent to defects in the BNNT sidewall (Figure 4a). The formation of these lines is reversible (Video S1, Supporting Information), so it is attributed to a temporary reduction in the translational and rotational motion of the encapsulated t‐Se chains rather than a phase change to a fully crystalline species (a process that would be irreversible under our conditions). Importantly, we observed the vacancy defects in BNNT not only propagate but also re‐arrange under the electron beam, causing the BNNT's diameter to contract. This is consistent with studies by Celik‐Aktas et al. and Cheng et al., where defects in the BNNT sidewalls were able to anneal in the presence of an electron beam, reorganising into a smaller diameter structure.^[^ ^48^, ^49^ ^]^ The same process here causes the phase change (t‐Se to l‐Se) of encapsulated Se due to the gradual reduction in BNNT diameter. A similar electron‐beam‐induced extrusion process has been previously reported for carbon nanotubes in Fe@MWCNTs, which required simultaneous in situ heating to 600 °C during electron beam irradiation.^[^ ^50^ ^]^ As the diameter of host BNNT decreased, we observed the formation of increasingly narrow Se NWs with more pronounced atomic spacings of t‐Se interchain separation, matching those seen previously in narrow CNTs. Further irradiation ultimately led to a linear Se chain (Figure 4c,f, inner diameter of BNNT 0.8 nm), agreeing with the prediction of the 1D phase diagram derived from CNT hosts (Figure 2m). Linear atomic chains of l‐Se (Figure 4c), seven t‐Se chains (Figure 4b) and smeared atomic contrast in larger NTs (Figure 4h) inside BNNTs mirror the observations in CNT (Figure 2d,i,j). While the interactions of Se with the host nanotube are predicted to be stronger in the case of BNNTs,^[^ ^43^ ^]^ they are not strong enough to prevent translational movement of the encapsulated nanowires (Figure S23, Supporting Information). We expect the BNNTs with narrowing diameter produced during electron beam irradiation to be stable, as discussed by Celik‐Aktas et al.^[^ ^48^ ^]^ and Cheng et al.^[^ ^49^ ^]^ and thus Se nanowires templated in real time under these conditions also to be stable as they appear to replicate nanowires that exist within CNTs under ambient conditions.

To probe this reaction further, the internal diameters of an empty BNNT, a Se‐filled BNNT and a Se‐filled CNT (all possessing initial diameters of ≈ 3.5 nm) were measured as a function of increasing electron dose at 200 kV. After administering a dose of 25 × 10^8^ e^−^nm^−2^, the internal diameter of the Se‐filled BNNT decreased by roughly 50% (Figure 4g–j; Video S2, Supporting Information). In contrast, the empty BNNT and Se‐filled CNT (Figure 4i; Figure S26, Supporting Information) saw an overall diameter reduction of less than 10%. Quantitative STEM‐EDX and local probe core–shell EELS analysis confirmed that electron beam irradiation of bundles of Se@BNNT caused a decrease in the amount B, N and Se, and minimal change in the structure of the B and N K‐edges, respectively (Figure S27, Supporting Information). Because of this, we expect very little change to the valence states of B and N atoms during irradiation. Furthermore, the interwall spacing of the BNNT remains consistent throughout the electron beam irradiation and the shrinkage of the BNNT (Figure 4a–c). This indicates that selenium atoms are not integrated into the hexagonal BN lattice, which continuously reconstructs itself under the electron beam.

The impact of Se on the contraction of BNNT in the electron beam is likely to relate to an effective bonding between Se and B or N dangling bonds in vacancies created by the electron beam (Figure S28, Supporting Information). This is supported by the appearance of defined contrast corresponding to t‐Se chains adjacent to newly formed defects (Figure 4a; Figure S24, Supporting Information). Such Se‐N or Se‐B bonding with defects causes the stabilisation of the latter, therefore reducing the barrier for defect formation in BNNT according to the Bell‐Evans‐Polanyi principle (Figure S29, Supporting Information).^[^ ^51^ ^]^ Unlike in BNNT, the presence of Se does not facilitate defect formation in CNTs, which may be related to the carbon lattice's ability to readily form five‐membered rings and redistribute the strain of the vacancies through Stone‐Wales rearrangement that slows down diameter constriction in CNT under the electron beam (Figure S30, Supporting Information).^[^ ^52^ ^]^

### 2.4 The Relationship Between Se NW Diameter and Bandgap

Ultra‐low‐loss EELS measurements can be used to determine the size and nature of optical bandgaps of nanoscale materials. Combining highly monochromated EELS with aberration‐corrected STEM (AC‐STEM) allows for the experimental determination of optical bandgaps with an extremely high energy and spatial resolution, uncovering structure‐property relationships at the nanoscale.^[^ ^53^ ^]^ This has been successfully performed on a range of nanomaterials, including CsPbBr_3_ nanosheets,^[^ ^54^ ^]^ ZnSe@ZnTe NWs^[^ ^55^ ^]^ and empty CNTs^[^ ^56^ ^]^ and BNNTs.^[^ ^57^ ^]^

The metallic or small‐gap semiconductor characteristics of CNTs result in a variety of electronic transitions, corresponding to van Hove singularities and π plasmons, which obscure the encapsulated semiconductors.^[^ ^56^ ^]^ However, considering that BNNTs have a bandgap of ≈ 5.5 eV, much wider than any of the forms of selenium,^[^ ^58^, ^59^ ^]^ optical transitions of semiconducting species encapsulated in BNNTs should be directly observable.

**Figure 5:**
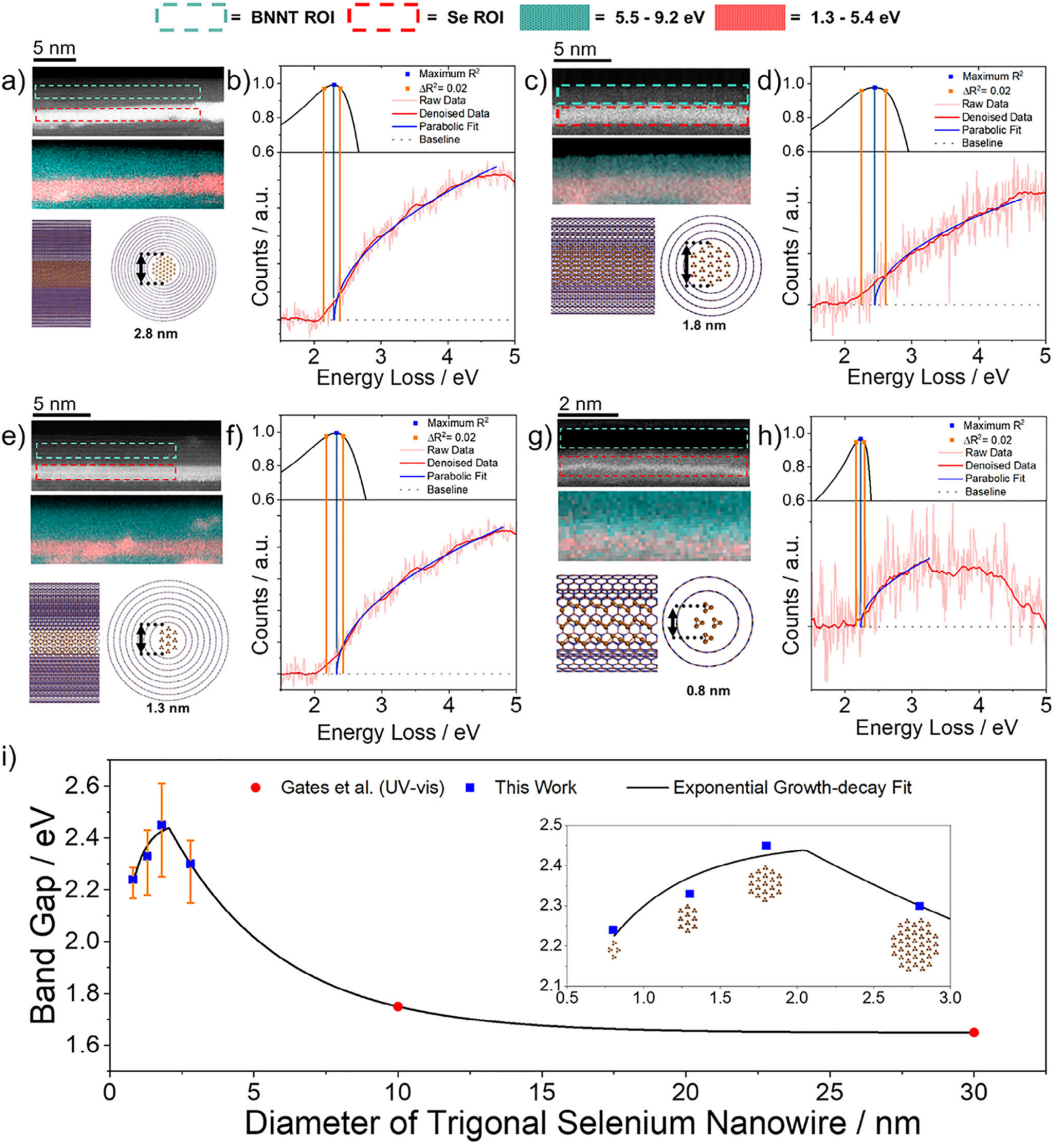
STEM‐EELS Bandgap Analysis of Se NWs Inside BNNTs a) 60 kV HAADF STEM image of Se@BNNT (top), false colour AC‐STEM‐EELS map illustrating semiconducting (pink) and insulating (cyan) regions, respectively, of the area shown above (middle) and a side on and end on molecular model of the Se@BNNT shown above, b) curve fitting of the ultra‐low‐loss EEL spectrum corresponding to the encapsulated Se shown in a), c) and d), e) and f) and g) and h) show the same as a) and b) but for Se NWs of different diameters, i) shows a summary of the bandgap energies of t‐Se NWs as their diameter is reduced, fitted to an exponential growth‐decay curve to guide the eye.

We developed a robust method for acquiring and processing the EELS data for individual Se NWs (Methods section, raw data in Figure S31, Supporting Information). High‐angle annular dark‐field (HAADF) AC‐STEM imaging (**Figure** **5**a,c,e,g) and subsequent AC‐STEM‐EELS analysis (Figure 5b,d,f,h) was performed at 60 kV, to prevent knock‐on damage and subsequent changes to Se@BNNT diameter during data acquisition. Additionally, the filled BNNTs analysed by STEM‐EELS were all above 1.4 nm in internal diameter, which, according to the 1D phase diagram (Figure 2n), will template the growth of NWs with different diameters but consisting of t‐Se chains of same disordered crystalline phase.

Our EELS measurements revealed that all Se NWs have a direct optical bandgap (Figure 5b,d,f,h). Systematic investigation showed that the relationship between the diameter of t‐Se NWs and their optical bandgap energy is non‐monotonic (Figure 5i; Table S7, Supporting Information) and consistently higher than that of bulk t‐Se reported by Gates et al.^[^ ^10^ ^]^ Notably, the bandgap reaches a maximum value of 2.45 eV for a 1.8 nm diameter BNNT, when the t‐Se NW consists of ≈20 helixes of t‐Se (Figure 5c,i, inset). This effect is attributed to quantum confinement.^[^ ^10^, ^60^ ^]^ However, as the BNNT internal diameter decreases from 1.8 to 0.8 nm, the energy of the bandgap decreases, reaching 2.24 eV at 0.8 nm, which corresponds to 4 helixes of t‐Se (Figure 5g,i inset). In the context of quantum confinement effects, the observed reduction in the bandgap in the sub‐nanometre regime is surprising. This phenomenon may be linked to the unique structural flexibility of Se chains. Previous studies have shown that the decrease in the bandgap of t‐Se chains confined within zeolites is associated with pressure‐induced distortions in Se─Se bond angles.^[^ ^61^ ^]^ Furthermore, theoretical studies of t‐Se chains under high pressure have predicted the same effect, with the pressure‐induced distortion of t‐Se bond angles causing a rapid decrease in the bandgap.^[^ ^62^ ^]^ This phenomenon of pressure‐induced bandgap decrease has also been reported in perovskites, attributed once again to bond distortion.^[^ ^63^, ^64^ ^]^ Our TEM imaging showed that bond angle distortions easily occur in individual selenium nanowires (Figures 2 and 4) when the internal diameter of the host nanotube approaches 1 nm, ultimately leading to the straightening of the helical t‐Se chain into a linear form, l‐Se. This transformation is likely related to theoretically predicted interactions of Se with nanotube walls.^[^ ^43^ ^]^ A key characteristic of our material systems is that when the diameter of the host nanotube decreases below 1.8 nm, the majority of the encapsulated t‐Se helixes come into direct contact with the walls of the host nanotube. This contact results in a greater distortion of the Se─Se bond angles, which may explain why the bandgap energy reaches its maximum at 1.8 nm. Below this diameter, any conformational changes tend to counteract the increase in the bandgap that is typically expected due to quantum confinement, thereby explaining the non‐monotonic correlation, which peaks at 1.8 nm (Figure 5i). Interestingly, single‐NW EELS measurements show bandgap values that are consistent with the orange colour of the Se@BNNT macroscopic material (powder; Figure 1a; Figure S32, Supporting Information) and resonance Raman analysis of Se@CNT (Figures S33–S36, Supporting Information).

## 3 Conclusion

We demonstrated a templated growth of selenium nanowires (Se NWs) with diameters ranging from a few nanometers to sub‐nanometer. The host carbon nanotubes provided an excellent platform for imaging the atomic structures of encapsulated Se. This allowed us to correlate the diameter of the nanotubes with the atomic structures of the NWs, leading to a 1D phase diagram that predicts the structure and bonding in Se NWs as a function of the internal diameter of the host nanotube. Furthermore, we imaged the dynamics of Se NW phase transitions, in real time and direct space, by applying a carefully controlled flux of an 80 kV electron beam to the host nanotube. This process involved the gradual reduction of the nanotube's diameter, resulting in the extrusion of a 2 nm NW down to a sub‐nm single linear Se chain – a process we imaged step‐by‐step. Using ultra‐low‐loss AC‐STEM‐EELS, we analysed the optical bandgap of individual Se NWs. We found that while the bandgap of NWs is consistently higher than that of bulk Se due to quantum confinement effects, conformational distortions of the t‐Se chains caused by interactions with the walls of the host nanotube become significant at diameters below 1.8 nm. These distortions lead to a decrease in the bandgap in NWs with diameters approaching 1 nm, thereby counteracting the effects of quantum confinement. Overall, the rich structural complexity of the single‐element semiconductor Se, achieved solely through nanoscale confinement, demonstrates the excellent tunability of this material's structure and properties, which can be precisely controlled by altering the diameter of the host nanotube. This can be achieved either by choosing host‐nanotubes of a specific diameter or by adjusting the nanotube diameter in situ using an electron beam as a tuning tool.

Qin et al.^[^ ^42^ ^]^ and Milligan et al.,^[^ ^38^ ^]^ have shown that semiconductors encapsulated in boron nitride nanotubes (BNNTs) can be effectively integrated into nanoscale field‐effect transistors and optoelectronic devices. While these devices have many attractive properties, such as an ideal form factor, high performance and high chemical stability, understanding and controlling the fundamental properties of the encapsulated semiconductors is vital if they are to find wider applications. Our study demonstrates that the dimensions, atomic structures and bandgap of semiconducting nanowires can be precisely adjusted within boron nitride nanotubes. This ability to tune fundamental properties presents exciting opportunities for creating and characterising electronic devices with customized functionalities. Our research shows that these adjustments can be achieved using only an electron beam, which serves as a three‐in‐one tool for imaging, spectroscopy, and nano‐fabrication.

## 4 Experimental Section

### Materials

CNTs were acquired from Nanointegris (HiPCO SWCNT), Carbon Solutions (P2 SWCNT), Nanocyl (Nanocyl 2100s) and NANOLAB (PD30 MWCNT). BNNTs (BNNT LLC) were used as received. Selenium and all solvents were purchased from Sigma‐Aldrich. TEM grids were purchased from Agar Scientific.

### Synthesis

In order to prepare CNTs for filling with Se, they must first be opened by removing their end‐caps. This procedure involves either thermal annealing or acid washing of the pristine CNTs, the latter of which has the added benefit of removing residual metal catalyst present from the synthesis of the CNTs. TGA analysis (Figure S37; Table S8, Supporting Information) was utilized to confirm the removal of reactive end caps and reduction in residual metal catalyst. Unlike CNTs, the BNNTs used in this study possessed neither residual catalyst from their synthesis, or end caps, meaning they could be utilized as a container for Se NWs in their pristine state.

### Opening of HiPCO SWCNTs

HiPCO SWCNTs (30 mg) were refluxed in concentrated nitric acid (10 mL, 83 °C, 3 h). The reaction mixture was then diluted with water (1 L) and filtered to give opened HiPCO SWCNTs (23 mg) as a black powder.

### Opening of P2 SWCNTs

P2 SWCNTs (250 mg) were heated in air (600 °C, 17 mins) and then sonicated in concentrated hydrochloric acid (250 mL, 1 h). The reaction mixture was then diluted with water (2 L) and filtered. The resulting solid was washed with acetone (1 L), to give opened P2 SWCNTs (106 mg) as a black powder.

### Opening of Nanocyl 2100s

Nanocyl 2100s (60 mg) were refluxed in concentrated nitric acid (20 mL, 83 °C, 3 h). The reaction mixture was then diluted with water (1 L) and filtered to give opened Nanocyl 2100s (48 mg) as a black powder.

### Opening of PD30 MWCNTs

PD30 MWCNTs (60 mg) were refluxed in concentrated nitric acid (20 mL, 83 °C, 3 h). The reaction mixture was then diluted with water (1 L) and filtered to give opened PD30 MWCNTs (51 mg) as a black powder.

### Encapsulation of Se into NTs

A sublimation filling method was utilized to synthesize both Se@CNT and Se@BNNT. Experimental conditions of heating to 550 °C at a pressure of 10^−5^ mbar for 70h were used, derived from previously reported conditions.^[^ ^28^ ^]^ A CS_2_ washing step was utilized after filling to remove any externally bound Se.

Opened CNTs or pristine BNNTs (10 mg) and Se (10 mg) powder were sealed under vacuum (10^−5^ mbar) in a Pyrex ampoule and heated to 550 °C for 70 h. The resultant material was then washed with CS_2_ (100 mL, stirring, 45 min), giving Se@NTs.

### Transmission Electron Microscopy

Samples were suspended in propan‐2‐ol and drop‐cast onto lacey‐carbon‐coated copper TEM grids. TEM imaging was conducted using a JEOL 2100F FEG‐TEM microscope operated at 200 kV at the Nanoscale and Microscale Research Centre (nmRC), University of Nottingham. AC‐HRTEM was performed using a chromatic (C_C_) and spherical (C_S_) aberration‐corrected SALVE TEM microscope operated at 60 kV at Ulm University. Electron fluence calculations were performed by measuring pixel counts per unit area using GMS 3.52.

### Transmission Electron Microscopy Simulations

Structural models of filled nanotubes were created using the Discovery Studio Viewer software as well as coordinate files from existing structures published in the literature, found through the ICSD. NTs were constructed using VMD software. TEM image simulations were carried out using QSTEM, a multislice program which uses the Dirac–Fock scattering potential of Rez et al.^[^ ^65^, ^66^ ^]^ A fixed number of 20 slices per nanotube was chosen, and images were calculated with a sampling set to match experimental conditions. The defocus and aberration parameters were set according to the values used in experimental imaging. The effect of limited electron dose was emulated by applying noise to the produced simulated images using a custom‐made Monte‐Carlo program exploiting the Poisson statistics of electrons. The structural coordinates of t‐Se were taken from Cherin et al., and the van Der Waals radius of Se used to create co‐linear l‐Se structures were taken from Cherin et al.^[^ ^40^ ^]^ and Bondi,^[^ ^41^ ^]^ respectively.

### Scanning Transmission Electron Microscopy‐Energy Dispersive X‐ray Analysis

Local EDX spectra were acquired for samples mounted on lacey‐carbon‐coated copper TEM grids using an Oxford Instruments XMax 80 X‐ray microanalysis system. STEM‐EDX mapping together with HAADF imaging was performed on a Thermo Fisher Talos 200X operated at 120 kV equipped with a windowless four‐segment SuperX EDX detector.

### Raman Spectroscopy

Micro Raman spectroscopy was performed using either a HORIBA LabRAM HR Raman or HORIBA LabRAM HR Evo microscope. Spectra were acquired using one or more of four different laser wavelengths: 325, 532, 633 and 785 nm. Spectra acquired at 1% (10% at 325 nm) power, a 100× objective (or 40x at 325 nm) and a 200 µm confocal pinhole. To simultaneously scan a range of Raman shifts, a rotatable diffraction grating (300 lines mm^−1^ at 785 nm, 600 lines mm^−1^ at 532 and 633 nm and 1200 lines mm^−1^ at 325 nm) along a path length of 800 mm was employed. Spectra were detected using a Synapse CCD detector (1024 pixels) thermoelectrically cooled to −60 °C. Spectra were taken from at least three random locations and averaged. Before spectra collection, the instrument was calibrated using the zero‐order line and a standard Si(100) reference band at 520.7 cm^−1^. The spectral resolution is better than 2.5 (325 nm/1200 lines mm^−1^), 1.8 (532 nm/600 lines mm^−1^), 0.8 (633 nm/600 lines mm^−1^) and 1.7 cm^−1^ (785 nm/300 lines mm^−1^) in these configurations.

### Scanning Transmission Electron Microscopy‐Energy Electron Loss Spectroscopy and Local Energy Electron Loss Spectroscopy

Scanning transmission electron microscopy (STEM) measurements and STEM‐EELS analysis were performed at 60 kV acceleration voltage on a Nion UltraSTEM 100MC ’HERMES’ at the SuperSTEM laboratory, Daresbury, UK. The microscope has a cold FEG emitter that has a native energy spread of 0.3 eV and it is equipped with a monochromator able to reach <10 meV energy resolution, while also possessing a C_5_ Nion QO corrector, with direct correction up to C_5,6_ for high spatial resolution (< 0.8 Å at 100 kV). EELS data was denoised using principal component analysis, as implemented in Gatan Microscopy Suite 3.5 (GMS3.5), with residuals carefully inspected to avoid the introduction of artefacts.

After AC‐STEM image acquisition, AC‐STEM‐EELS was utilized to acquire ultra‐low‐loss EELS maps of the respective nanocomposite materials. Two regions of interest (ROIs) of identical size were selected, producing two EEL spectra of the Se NW inside the BNNT and a reference area of BNNT sidewall. Next, the BNNT sidewall reference spectrum is subtracted from the Se NW spectrum (except in the case of the Se@BNNT material shown in Figure 5g, where an adjacent empty BNNT is used as a reference due to the encapsuling BNNT possessing fewer sidewalls), coined by L. M. Brown as the ’Difference Method’.^[^ ^67^, ^68^ ^]^ The noise in the resulting difference spectra was reduced by a Savitzky‐Golay smoothing filter (second order, 61 points, conditions used previously by Keller et al.).^[^ ^69^ ^]^ Finally, an automated fixed endpoint fitting method, published previously by Granerød et al.,^[^ ^70^ ^]^ was utilized to determine the onset of indirect optical bandgap transition for encapsulated Se. The endpoint was determined as the highest intensity point of the optical bandgap transition. An R^2^ reduction of 0.02 was chosen to indicate the uncertainty in the onset determination, the same as in work by Granerød et al.^[^ ^70^ ^]^

Local EELS analysis was performed using a Gatan Enfinium SE spectrometer at the nmRC, University of Nottingham, on a JEOL 2100 plus microscope at 200 kV.

### Thermogravimetric Analysis

A TA Q500 Thermogravimetric Analyser was used for the thermogravimetric analysis. All samples were analysed using a platinum pan and in the presence of air. Experimental parameters were as follows: 10 min isothermal hold at room temperature, ramp from room temperature to 1000 °C at 10 °C min^−1^, followed by a final 10 min isothermal hold at 1000 °C.

## Conflict of Interest

The authors declare no conflict of interest.

## Author Contributions

W.J.C., A.N.K., A.P., and Q.M.R. conceptualized the project; W.J.C. designed experiments, synthesized materials, performed TEM, EDX, STEM‐EDX, STEM‐EELS and TGA analysis, performed TEM simulations, interpreted the data and wrote the initial manuscript; Q.M.R. performed AC‐STEM‐EELS analysis and data interpretation; JB performed AC‐HRTEM imaging and STEM‐EDX mapping; G.A.R. performed and interpreted Raman analysis; I.C.Z., B.L.W., and M.W.F. assisted with fluence calculations, processing and interpretation of AC‐STEM‐EELS data and acquisition of STEM‐EELS data; R.R.W. and L.R.S. assisted with processing pristine BNNTs before filling with Se; A.N.K. and J.A.F. assisted in writing the manuscript; A.N.K., J.A.F., A.P., Q.M.R., and U.K. provided supervision. All authors contributed to editing the manuscript.

## Supporting information



Supporting Information

Supplemental Video 1

Supplemental Video 2

## Data Availability

The data that support the findings of this study are available from the corresponding author upon reasonable request.

## References

[1] R. Boyd , Nat. Chem. 2011, 3, 570. 21697880 10.1038/nchem.1076

[2] W. Smith , Nature 1873, 7, 303.

[3] W. Smith , J. Soc. Telegraph Eng. 1873, 2, 31.

[4] W. G. Adams , R. E. Day , Phil. Trans. R. Soc. 1877, 167, 313.

[5] C. E. Fritts , Am. J. Sci. 1883, s3‐26, 465.

[6] N. Bisht , P. Phalswal , P. K. Khanna , Mater. Adv. 2022, 3, 1415.

[7] J. K. Qin , F. Zhou , J. Wang , J. Chen , C. Wang , X. Guo , S. Zhao , Y. Pei , L. Zhen , P. D. Ye , S. P. Lau , Y. Zhu , C. Y. Xu , Y. Chai , ACS Nano 2020, 14, 10018. 32806043 10.1021/acsnano.0c03124

[8] B. A. Al Jahdaly , N. S. Al‐Radadi , G. M. G. Eldin , A. Almahri , M. K. Ahmed , K. Shoueir , I. Janowska , J. Mater. Res. Technol. 2021, 11, 85.

[9] W. Huang , M. Wang , L. Hu , C. Wang , Z. Xie , H. Zhang , Adv. Funct. Mater. 2020, 30, 2003301.

[10] B. Gates , B. Mayers , B. Cattle , Y. Xia , Adv. Funct. Mater. 2002, 12, 219.

[11] Y. De Chiou , Y. J. Hsu , Appl. Catal. B 2011, 105, 211.

[12] J. Qin , G. Qiu , J. Jian , H. Zhou , L. Yang , A. Charnas , D. Y. Zemlyanov , C. Y. Xu , X. Xu , W. Wu , H. Wang , P. D. Ye , ACS Nano 2017, 11, 10222. 28949510 10.1021/acsnano.7b04786

[13] Z. Wang , J. Jing , Y. Ren , Y. Guo , N. Tao , Q. Zhou , H. Zhang , Y. Ma , Y. Wang , Mater. Lett. 2019, 234, 212.

[14] T. K. Todorov , S. Singh , D. M. Bishop , O. Gunawan , Y. S. Lee , T. S. Gershon , K. W. Brew , P. D. Antunez , R. Haight , Nat. Commun. 2017, 8, 682. 28947765 10.1038/s41467-017-00582-9 PMC5613033

[15] Z. Li , L. Yin , Nanoscale 2015, 7, 9597. 25951942 10.1039/c5nr00903k

[16] S. T. Skowron , T. W. Chamberlain , J. Biskupek , U. Kaiser , E. Besley , A. N. Khlobystov , Acc. Chem. Res. 2017, 50, 1797. 28696097 10.1021/acs.accounts.7b00078

[17] K. L. Y. Fung , B. L. Weare , M. W. Fay , S. P. Argent , A. N. Khlobystov , Micron 2023, 165, 103395. 36543056 10.1016/j.micron.2022.103395

[18] B. W. Smith , M. Monthioux , D. E. Luzzi , Nature 1998, 396, 323.

[19] E. Faulques , N. Kalashnyk , C. A. Slade , A. M. Sanchez , J. Sloan , V. G. Ivanov , Synth. Met. 2022, 284, 116968.

[20] W. J. Cull , S. T. Skowron , R. Hayter , C. T. Stoppiello , G. A. Rance , J. Biskupek , Z. R. Kudrynskyi , Z. D. Kovalyuk , C. S. Allen , T. J. A. Slater , U. Kaiser , A. Patane , A. N. Khlobystov , ACS Nano 2023, 17, 6062. 36916820 10.1021/acsnano.3c00670 PMC10061931

[21] R. J. Kashtiban , C. E. Patrick , Q. Ramasse , R. I. Walton , J. Sloan , Adv. Mater. 2023, 3, 2208575. 10.1002/adma.202208575 36528852

[22] T. Fujimori , A. Morelos‐Gómez , Z. Zhu , H. Muramatsu , R. Futamura , K. Urita , M. Terrones , T. Hayashi , M. Endo , S. Y Hong , Y. C Choi , D. Tománek , K. Kaneko , Nat. Commun. 2013, 4, 1. 10.1038/ncomms3162 PMC3717502 23851903

[23] T. Fujimori , R. B. Dos Santos , T. Hayashi , M. Endo , K. Kaneko , D. Tománek , ACS Nano 2013, 7, 5607. 23683115 10.1021/nn4019703

[24] P. V. C. Medeiros , S. Marks , J. M. Wynn , A. Vasylenko , Q. M. Ramasse , D. Quigley , J. Sloan , A. J. Morris , ACS Nano 2017, 11, 6178. 28467832 10.1021/acsnano.7b02225

[25] A. Loiseau , H. Pascard , Chem. Phys. Lett. 1996, 256, 246.

[26] D. Dutta , S. Gope , D. S. Negi , R. Datta , A. K. Sood , A. J. Bhattacharyya , J. Phys. Chem. C 2016, 120, 29011.

[27] J. Chancolon , F. Archaimbault , A. Pineau , S. Bonnamy , J. Nanosci. Nanotechnol. 2006, 6, 82. 16573074

[28] K. Kobayashi , H. Yasuda , Chem. Phys. Lett. 2015, 634, 60.

[29] N. G. Chopra , R. J. Luyken , K. Cherrey , V. H. Crespi , M. L. Cohen , S. G. Louie , A. Zettl , Science 1995, 269, 966. 17807732 10.1126/science.269.5226.966

[30] D. Golberg , Y. Bando , C. Tang , C. Zni , Adv. Mater. 2007, 19, 2413.

[31] H. R. Barzegar , T. Pham , A. V. Talyzin , A. Zettl , Phys. Status Solidi B Basic Res. 2016, 253, 2377.

[32] A. Cadena , Á. Pekker , B. Botka , E. Dodony , Z. Fogarassy , B. Pécz , K. Kamarás , Phys. Status Solidi – Rapid Res. Lett. 2023, 17, 2200284.

[33] N. Koi , T. Oku , M. Nishijima , Solid State Commun. 2005, 136, 342.

[34] W. Q. Han , C. W. Chang , A. Zettl , Nano Lett. 2004, 4, 1355.

[35] K. E. Walker , G. A. Rance , Á. Pekker , H. M. Tóháti , M. W. Fay , R. W. Lodge , C. T. Stoppiello , K. Kamarás , A. N. Khlobystov , Small Methods 2017, 1, 1700184.

[36] C. Allard , L. Schué , F. Fossard , G. Recher , R. Nascimento , E. Flahaut , A. Loiseau , P. Desjardins , R. Martel , E. Gaufrès , Adv. Mater. 2020, 32, 2001429. 10.1002/adma.202001429 32483892

[37] S. Van Bezouw , D. H. Arias , R. Ihly , S. Cambré , A. J. Ferguson , J. Campo , J. C. Johnson , J. Defillet , W. Wenseleers , J. L. Blackburn , ACS Nano 2018, 12, 6881. 29965726 10.1021/acsnano.8b02213 PMC6083417

[38] G. M. Milligan , D. L. M. Cordova , Z. F. Yao , B. Y. Zhi , L. R. Scammell , T. Aoki , M. Arguilla , Chem. Sci. 2024, 15, 10464. 38994401 10.1039/d4sc01477d PMC11234864

[39] V. V. Poborchii , G. G. Lindner , M. Sato , J. Chem. Phys. 2002, 116, 2609.

[40] P. Cherin , P. Unger , Inorg. Chem. 1967, 6, 1589.

[41] A. Bondi , J. Phys. Chem. 1964, 68, 441.

[42] J. K. Qin , P. Y. Liao , M. Si , S. Gao , G. Qiu , J. Jian , Q. Wang , S. Q. Zhang , S. Huang , A. Charnas , Y. Wang , M. J. Kim , W. Wu , X. Xu , H. Y. Wang , L. Yang , Y. Khin Yap , P. D. Ye , Nat. Electron. 2020, 3, 141.

[43] R. A. Jishi , C. T. White , J. W. Mintmire , Int. J. Quantum Chem. 2000, 80, 480.

[44] Y. Chang , L. Huang , Y. Zhou , J. Wang , W. Zhai , ACS Appl. Mater. Interfaces 2022, 14, 5624. 35050577 10.1021/acsami.1c22909

[45] L. D. Laude , B. Fitton , J. Non Cryst. Solids 1972, 8–10, 971.

[46] B. Dittrich , IUCrJ 2021, 8, 305. 10.1107/S2052252521000531 PMC7924241 33708406

[47] T. W. Ebbesen , J. Phys., Chem. Solids 1996, 57, 951.

[48] A. Celik‐Aktas , J. F. Stubbins , J. M. Zuo , J. Appl. Phys. 2007, 102, 024310.

[49] G. Cheng , S. Yao , X. Sang , B. Hao , D. Zhang , Y. K. Yap , Y. Zhu , Small 2016, 12, 818. 26682873 10.1002/smll.201502440

[50] L. Sun , F. Banhart , A. V. Krasheninnikov , J. A. Rodriguez‐Manzo , M. Terrones , P. M. Ajayan , Science 1979, 312, 1199. 10.1126/science.1124594 16728637

[51] K. Cao , T. Zoberbier , J. Biskupek , A. Botos , R. L. McSweeney , A. Kurtoglu , C. T. Stoppiello , A. V. Markevich , E. Besley , T. W. Chamberlain , U. Kaiser , A. N. Khlobystov , Nat. Commun. 2018, 9, 3382. 30139935 10.1038/s41467-018-05831-z PMC6107508

[52] J. C. Meyer , F. Eder , S. Kurasch , V. Skakalova , J. Kotakoski , H. J. Park , S. Roth , A. Chuvilin , S. Eyhusen , G. Benner , A. V. Krasheninnikov , U. Kaiser , Phys. Rev. Lett. 2012, 108, 1. 10.1103/PhysRevLett.108.196102 23003063

[53] B. Rafferty , L. Brown , Phys. Rev. B Condens. Matter Mater. Phys. 1998, 58, 10326.

[54] R. Brescia , S. Toso , Q. Ramasse , L. Manna , J. Shamsi , C. Downing , A. Calzolari , G. Bertoni , Nanoscale Horiz. 2020, 5, 1610. 33140817 10.1039/d0nh00477d

[55] S. Martí‐Sánchez , M. Botifoll , E. Oksenberg , C. Koch , C. Borja , M. C. Spadaro , V. Di Giulio , Q. Ramasse , F. J. García de Abajo , E. Joselevich , J. Arbiol , Nat. Commun. 2022, 13, 1. 35835772 10.1038/s41467-022-31778-3 PMC9283334

[56] F. S. Hage , Q. M. Ramasse , Eur. Microscopy Congress 2016, 80, 405.

[57] R. Arenal , O. Stephan , M. Kociak , D. Taverna , C. Colliex , A. Rubio , A. Loiseau , AIP Conf. Proc. 2004, 723, 293.

[58] I. Hadar , X. Hu , Z. Z. Luo , V. P. Dravid , M. G. Kanatzidis , ACS Energy Lett. 2019, 4, 2137.

[59] F. Jiang , W. Cai , G. Tan , Nanoscale Res. Lett. 2017, 12, 1. 28610394 10.1186/s11671-017-2165-y PMC5468178

[60] N. S. Mohammad , J. Phys.: Condens. Matter 2014, 26, 423202. 25245123 10.1088/0953-8984/26/42/423202

[61] W. Ren , J. T. Ye , W. Shi , Z. K. Tang , C. T. Chan , P. Sheng , New J. Phys. 2009, 11, 103014.

[62] H. Akbarzadeh , S. J. Clark , G. J. Ackland , J. Phys.: Condens. Matter 1993, 5, 8065.

[63] L. Kong , G. Liua , J. Gong , Q. Hu , R. D. Schaller , P. Dera , D. Zhang , Z. Liu , W. Yang , K. Zhu , Y. Tang , C. Wang , S. H. Wei , T. Xu , H. K. Mao , Proc. Natl. Acad. Sci. U S A 2016, 113, 8910. 27444014 10.1073/pnas.1609030113 PMC4987786

[64] T. Geng , Z. Ma , Y. Chen , Y. Cao , P. Lv , N. Li , G. Xiao , Nanoscale 2020, 12, 1425. 31912845 10.1039/c9nr09533k

[65] C. Koch , Doctor of Philosophy , Arizona State University, Tempe, Arizona 2002.

[66] D. Rez , P. Rez , I. Grant , Acta Crystallogr. A 1994, 50, 481.

[67] Q. M. Ramasse , Ultramicroscopy 2017, 180, 41. 28385362 10.1016/j.ultramic.2017.03.016

[68] T. C. Lovejoy , Q. M. Ramasse , M. Falke , A. Kaeppel , R. Terborg , R. Zan , N. Dellby , O. L. Krivanek , Appl. Phys. Lett. 2012, 100, 154101.

[69] D. Keller , S. Buecheler , P. Reinhard , F. Pianezzi , D. Pohl , A. Surrey , B. Rellinghaus , R. Erni , A. N. Tiwari , Microsc. Microanal. 2014, 20, 1246. 24690441 10.1017/S1431927614000543

[70] C. S. Granerød , W. Zhan , Ø. Prytz , Ultramicroscopy 2018, 184, 39. 28843183 10.1016/j.ultramic.2017.08.006

