# Supplementary material for: Flexible Selenium Nanowires with Tuneable Electronic Bandgaps
Source: Adv Mater. 2025 May 21;37(32):2501821. doi: 10.1002/adma.202501821 (PMC12355565; doi:10.1002/adma.202501821)
Supplement: Supplementary file 1 — Supporting Information [file ADMA-37-2501821-s002.docx]

***Flexible selenium nanowires with tuneable electronic band gaps***

***Supporting Information***

*William J. Cull, Quentin M. Ramasse, Johannes Biskupek, Graham A. Rance, Ian Cardillo-Zallo, Benjamin L. Weare, Michael W. Fay, Roy R. Whitney, Lyndsey R. Scammell, Jesum Alves Fernandes, Ute Kaiser, Amalia Patanè and Andrei N. Khlobystov*

William J. Cull, Ian Cardillo-Zallo*,* Andrei N. Khlobystov

School of Chemistry, University of Nottingham, Nottingham NG7 2RD, United Kingdom
E-mail: andrei.khlobystov@nottingham.ac.uk

Quentin M. Ramasse

SuperSTEM Laboratory, SciTech Daresbury Campus, Daresbury, WA4 4AD, United

Kingdom

School of Chemical and Process Engineering, and School of Physics and Astronomy,

University of Leeds, Leeds LS2 9JT, United Kingdom

E-mail: [qmramasse@superstem.org](mailto:qmramasse@superstem.org)

Johannes Biskupek, Ute Kaiser

Electron Microscopy Group of Materials Science, Central Facility for Electron Microscopy, Ulm University, Ulm 89081, Germany

Graham A. Rance, Ian Cardillo-Zallo, Benjamin L. Weare, Michael W. Fay*,*

Nanoscale and Microscale Research Centre, University of Nottingham, Nottingham, NG7 2QL, United Kingdom

Roy R. Whitney, Lyndsey R. Scammell

BNNT LLC, Newport News, VA, USA

Amalia Patanè

School of Physics, University of Nottingham, Nottingham NG7 2RD, United Kingdom

**Contents**

List of Figures..........................................................................................................................S3

List of Tables...........................................................................................................................S4

List of Videos..........................................................................................................................S5

References..............................................................................................................................S40

**List of Figures:**

Figure S1: TEM and AC-TEM images of the NTs utilised in this study

Figure S2: Histograms showing the average internal and external diameter of the nanotubes used in this study

Figure S3: Resonance Raman analysis of Se, Se@CNTs and Se@BNNT

Figure S4: Summary of the resonance Raman analysis of Se@CNTs

Figure S5: EDX analysis of Se@CNT systems

Figure S6: STEM-EDX analysis of a single Se filled P2 SWCNT

Figure S7. AC-TEM image, AC-TEM simulations and contrast analysis of a linear atomic chain of Se inside a DWCNT

Figure S8. 60 kV AC-TEM image of Se@DWCNT, showing the longest length chain of l-Se imaged

Figure S9: AC-TEM images of fast-moving Se@CNT

Figure S10: AC-TEM images and AC-TEM simulations of a t-Se chain inside a SWCNT

Figure S11: Contrast Analysis of the TEM images shown in Figure S10

Figure S12: Rotational tableau showing 60 kV TEM simulations of a single t-Se nanowire encapsulated inside a (6,6) SWCNT.

Figure S13: AC-TEM image, AC-TEM simulations and contrast analysis of two chains of l-Se inside a SWCNT

Figure S14. 60 kV AC-TEM image of two co-linear l-Se chains twisting inside a SWCNT.

Figure S15: AC-TEM images and contrast analysis of two t-Se chains inside CNTs of different diameters

Figure S16. 60 kV AC-TEM image of two chains of t-Se, showing how the spacing between t-Se chains can decrease as a result of the two chains twisting inside the CNT

Figure S17: AC-TEM image and contrast analysis of Se@MWCNTs

Figure S18. AC-TEM images and line profile analysis of the Se structure shown in Figure S17, highlighting its mobility

Figure S19: TEM images showing the concave and convex menisci of Se@MWCNTs

Figure S20. TEM images showing the mobility of Se encapsulated by larger diameter CNTs, showing the mobility of the encapsulated Se

Figure S21: TEM simulations of multiple t-Se chains as they are rotated

Figure S22: EDX analysis of Se@BNNT

Figure S23: TEM images of fast moving Se@BNNT

Figure S24: TEM images and contrast analysis of Se@BNNT following continued irradiation

Figure S25: Un-cropped AC-TEM images of the AC-TEM images shown in Figures 4a, b and c

Figure S26: TEM images of an empty BNNT and Se@MWCNT following irradiation by an electron fluence of 25 x 108 e^-^nm^-2^ at 200 kV

Figure S27: STEM-EDX and local probe EELS of Se@BNNT before and after administering a high electron fluence.

Figure S28: Mechanism of e-beam defect formation in BNNT (80 kV)

Figure S29: Reaction coordinate diagram for the formation of defects and the shrinking of BNNTs under the electron beam

Figure S30: Mechanism of e-beam defect formation in CNTs (200 kV)

Figure S31: Raw EELS data used in Figures 5b, d, f, and h

Figure S32: Images of solid Se and Se@BNNT, showing how encapsulation (i.e., nanoconfinement) causes colour change

Figure S33: Resonance Raman Analysis of Se@P2 SWCNTs

Figure S34: Resonance Raman Analysis of Se@PD30 MWCNTs

Figure S35: Summary of resonance Raman analysis of Se@PD30 MWCNTs

Figure S36: Raman analysis of Se@BNNT with a 532 nm excitation laser

Figure S37: TGA analysis of the CNTs utilised in this study, before and after opening procedures

**List of Tables**

Table S1: Identity and technical data for the NTs utilised in this study

Table S2: Average internal diameters, external diameters and number of walls of the NTs utilised in this study

Table S3: Summary of the G-band and RBM positions shown in Figure S3

Table S4: Summary of the Se band positions shown in Figure S3

Table S5: Dimensions of Se and CNTs used for Figure 2m

Table S6: Dimensions of Se and CNTs used in Figure 2n

Table S7: Summary of the data shown in figure 5i

Table S8: Summary of TGA of the CNTs utilised in this study

**List of Videos**

Video S1: Movie of AC-TEM images of Se@BNNT at 80 kV

Video S2: Movie of TEM images of Se@BNNT at 200 kV

Table S1. Summary of the types of NTs utilised in this study.

| **Nanotube** | **Supplier** | **Synthesis Method** | **Impurities Present** |
| --- | --- | --- | --- |
| HiPCO^a^ SWCNT^[1]^ | Nanointegris | HiPCO | Amorph. C, Fe |
| P2 SWCNT^[2,3]^ | Carbon Solutions | Arc Discharge | Amorph. C, Ni, Y |
| Nanocyl 2100^[4,5]^ | Nanocyl | CVD^b^ | Amorph. C, Fe, Ni |
| PD30 MWCNT^[6]^ | NANOLAB | CVD | Amorph. C, Fe, S |
| BNNT^[7]^ | BNNT LLC | HTP^c^ | Amporh. B, h-BN |

^a^ HiPCO: **Hi**gh-**P**ressure **C**arbon M**o**noxide

^b^ CVD : **C**hemical **V**apor **D**eposition

^c^ HTP : **H**igh **T**emperature/ High **P**ressure


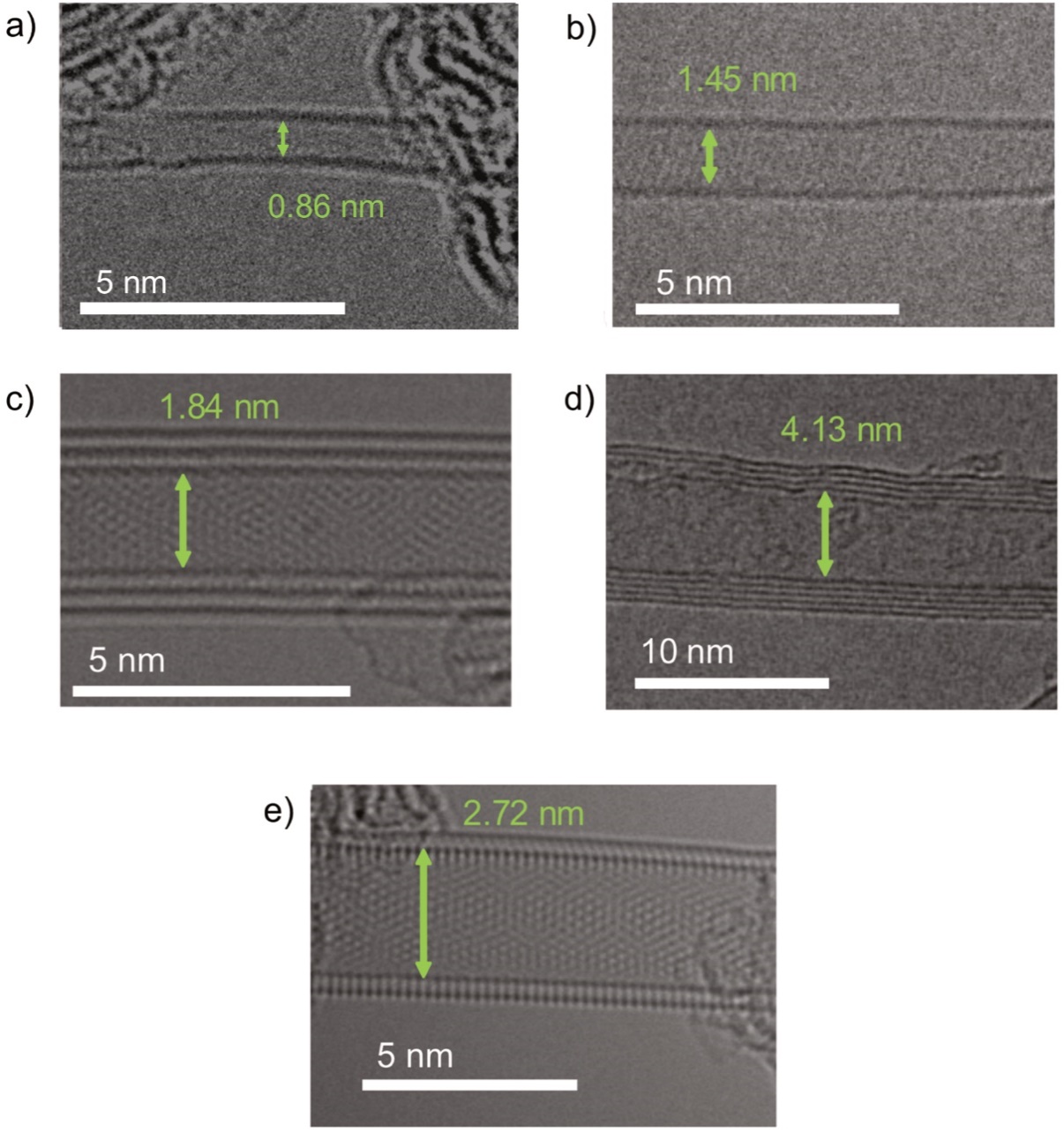


Figure S1. Representative TEM images of the NTs used in this study: a) HiPCO SWCNT, b) P2 SWCNT, c) Nanocyl 2100, d) PD30 MWCNT, and e) BNNT. a), b), c), and e) are 60 kV AC-TEM images. d) is a 200 kV TEM image.


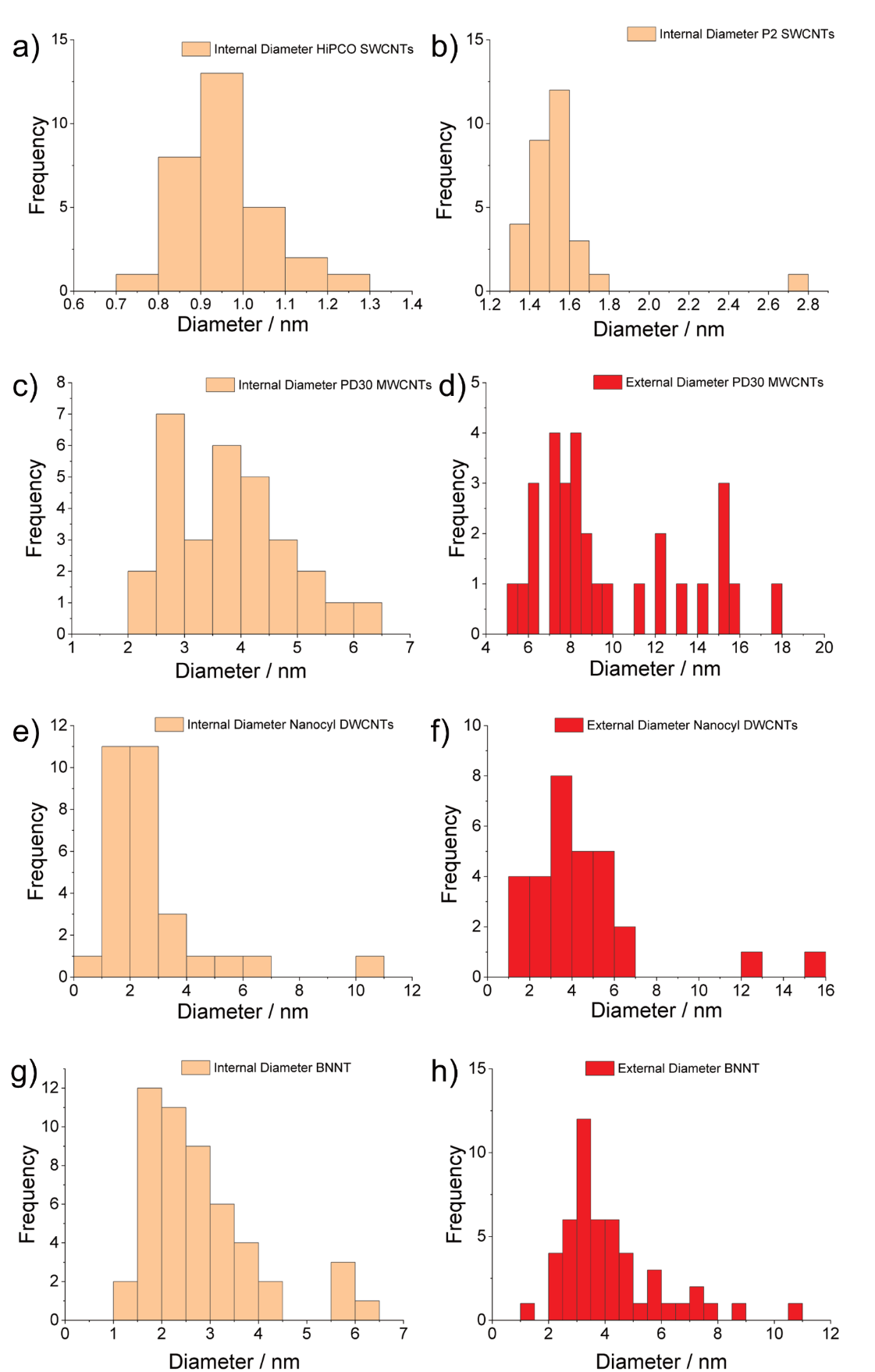


Figure S2. Histograms showing the diameter distributions of the NTs used in this study, a) internal diameter distribution of HiPCO SWCNTs, b) internal diameter distribution of P2 SWCNTs, c) internal diameter distribution of PD30 MWCNTs, d) external diameter distribution of PD30 MWCNTs, e) internal diameter distribution of Nanocyl DWCNTs, f) external diameter distribution of Nanocyl DWCNTs, g) internal diameter distribution of BNNTs, h) external diameter distribution of BNNTs.

Table S2. Summary of experimentally measured diameters and number of walls of BNNT and CNTs used in this study. N=30 and 50 for CNTs and BNNTs, respectively.

|  | **Internal diameter / nm** | | **External diameter / nm** | | **Number of Walls** | |
| --- | --- | --- | --- | --- | --- | --- |
| **Nanotube** | **Mean** | **Standard deviation** | **Mean** | **Standard deviation** | **Mean** | **Standard deviation** |
| HiPCO SWCNT | 0.95 | 0.12 | N/A | N/A | 1 | 0 |
| P2 SWCNT | 1.55 | 0.25 | N/A | N/A | 1 | 0 |
| Nanocyl 2100 | 2.75 | 1.89 | 4.42 | 3.01 | 3.23 | 2.01 |
| PD30 MWCNT | 3.79 | 1.04 | 9.82 | 3.57 | 9.27 | 4.71 |
| BNNT | 2.82 | 1.14 | 4.23 | 1.93 | 2.48 | 1.23 |


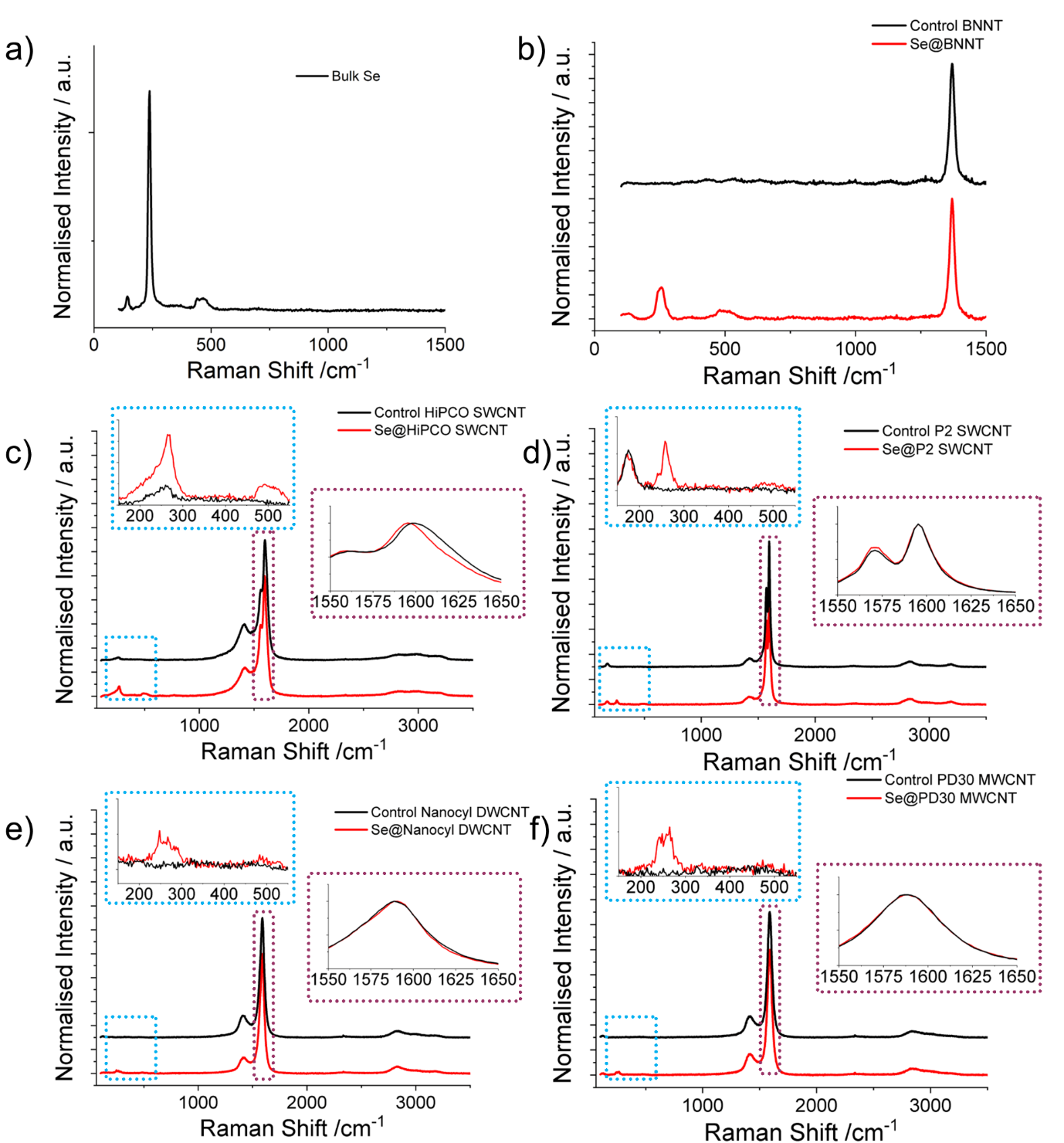
Figure S3. 325 nm Raman analysis of a) bulk Se, b) Se@BNNT (red) and control BNNT (black), c) HiPCO SWCNT (black) and Se@HiPCO SWCNT (red), where the dashed blue bordered inset (right) shows a zoomed in perspective of the RMB region and dashed maroon bordered inset shows a zoomed in perspective of the G-band region, d), e) and f) show the same but for control P2 SWCNT (black) and Se@ P2 SWCNT (red), control Nanocyl 2100 (black) and Se@Nanocyl 2100 (red) and control PD30 MWCNT (black) and Se@PD30 MWCNTs (red), respectively. Spectra have been baseline-corrected, normalised to the intensity of the spectral maximum and offset on the y-axis for ease of visual comparison.

Table S3. Summary of G-band and RBM position for control CNTs and Se@CNTs, extracted from the data shown in Figure S3.

| **Material** | **G Band Position / cm ^-1^** | **Radial Breathing Mode Position / cm^-1^** |
| --- | --- | --- |
| HiPCO SWCNT | 1600 | 262 |
| Se@HiPCO SWCNT | 1596 | n/a ^a^ |
| P2 SWCNT | 1595 | 175 |
| Se@P2 SWCNT | 1595 | 172 ^b^ |
| Nanocyl 2100 | 1591 | n/a ^c^ |
| Se@Nanocyl 2100 | 1591 | n/a ^c^ |
| PD30 MWCNT | 1589 | n/a |
| Se@PD30 MWCNT | 1589 | n/a |

^a^ The position of the RBM in Se@HiPCO cannot be accurately determined due to strong overlap with the stretching mode of t-Se.

^b^ A red shift in the position of the RBM in Se@P2 SWCNT, relative to P2 SWCNT, provides further evidence for the confinement of Se NWs inside these nanotubes.

^c^ The position of the RBM could not be determined for Nanocyl 2100 and Se@Nanocyl 2100 as the low-energy cut-off for the Rayleigh rejection filter used with the 325 nm laser was above the expected position for the RBMs.

Table S4. Summary of Se band positions for Solid Se and filled NT samples, extracted

from the data shown in Figure S3

| **Material** | **Se band position(s) / cm^-1^** |
| --- | --- |
| Bulk Se | 143, 237, 440 |
| Se@BNNT | 256, 479 |
| Se@HiPCO SWCNT | 270, 493 |
| Se@P2 SWCNT | 258, 484 |
| Se@Nanocyl 2100 | 248, 267 |
| Se@PD30 MWCNT | 243, 265 |


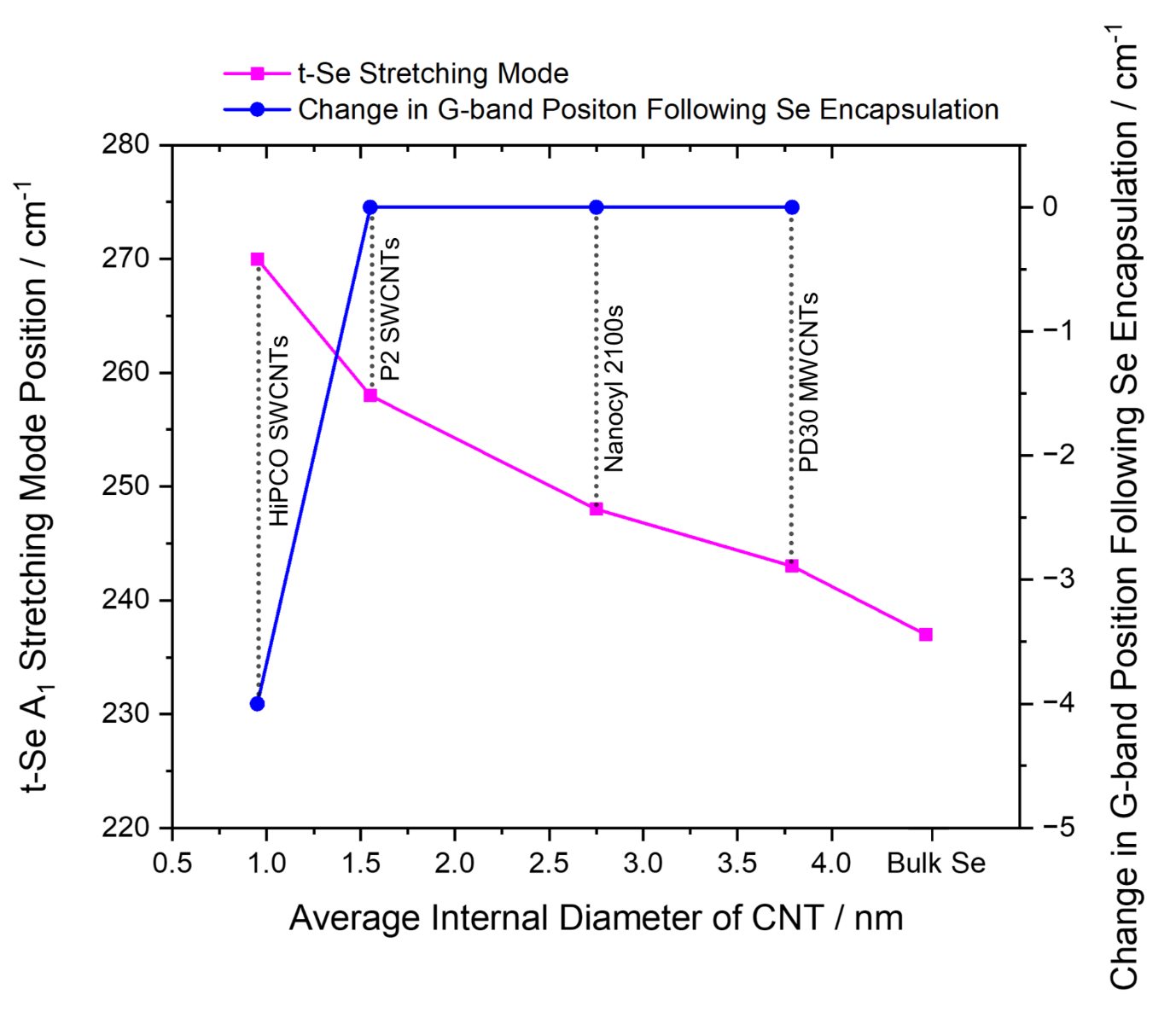


Figure S4. A summary of the 325 nm Raman analysis is shown in Figure S3. As the diameter of the t-Se is reduced by encapsulation inside smaller diameter CNTs, the position of the t-Se stretching mode (A_1_) blueshifts, an effect also seen when trigonal Te is encapsulated in CNTs.^[8]^ The A_1_ stretching mode in Se@BNNT (not shown in this graph, but the spectrum is shown in Figure S3b) also experiences a blueshift, confirming successful encapsulation of Se. In the smallest average diameter CNTs used in this study (HiPCO SWCNTs) there is a redshift in the position of the CNT G-band following encapsulation of Se, indicating electron transfer from the CNT to the Se. This likely suggests a good geometric match between host and guest.


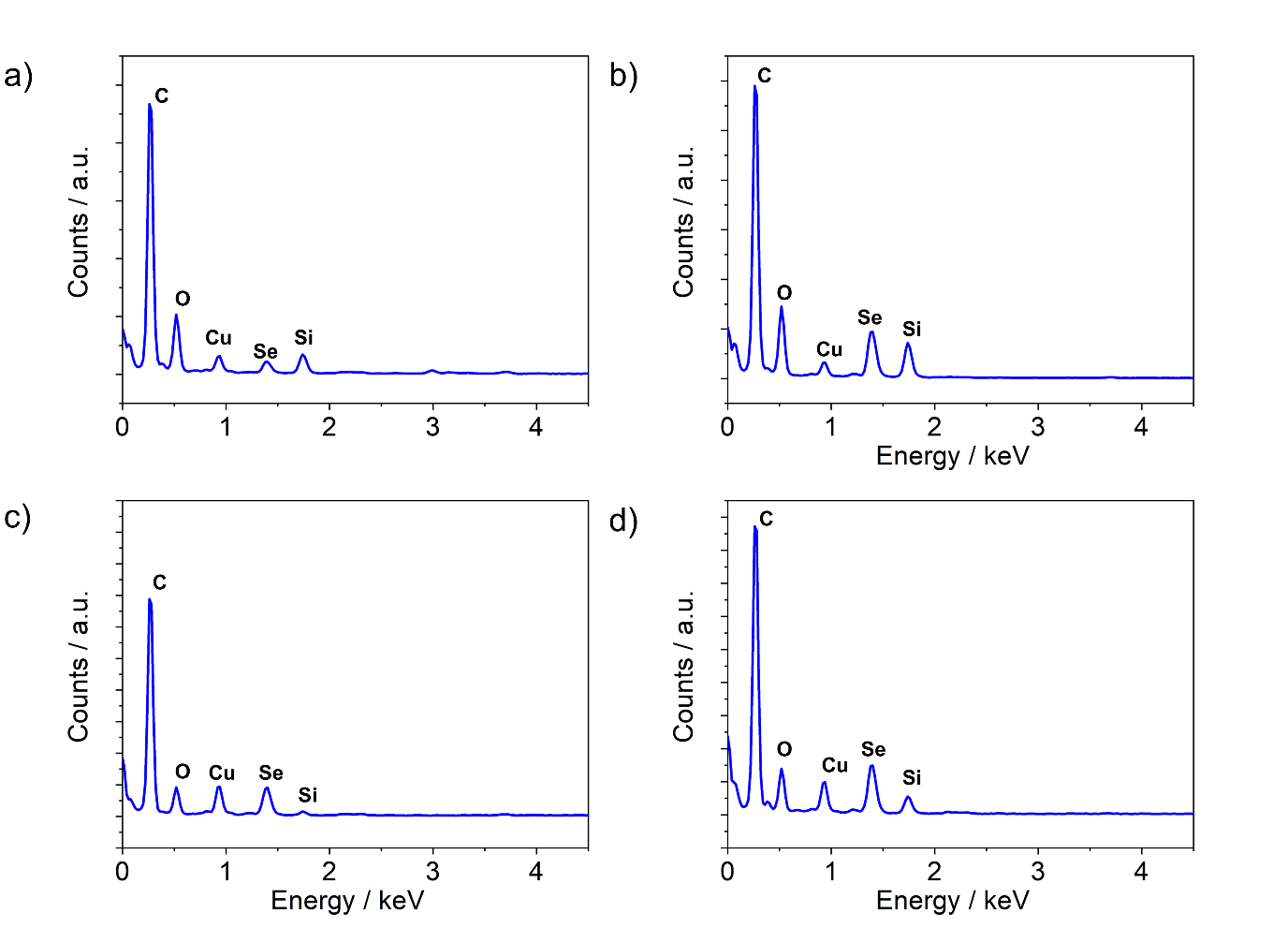


Figure S5. EDX analysis of the Se@CNT samples used in this study, a) Se@HiPCO SWCNTs, b) Se@P2 SWCNTs, c) Se@Nanocyl 2100s, d) Se@PD30 MWCNTs.


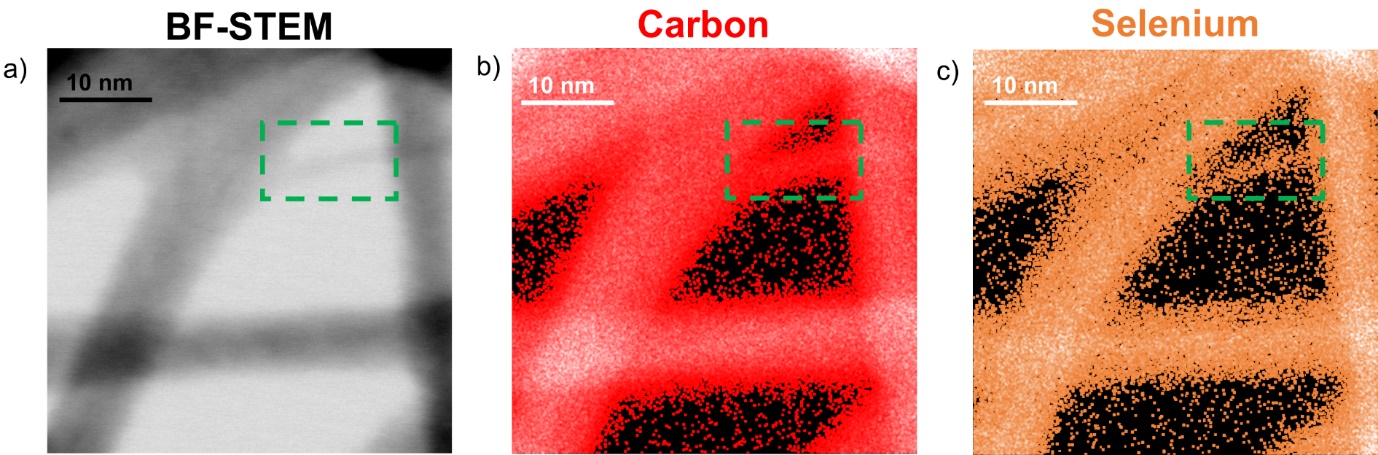


Figure S6. STEM-EDX analysis of Se@P2SWCNTs, a) Bright field STEM image, b) carbon elemental map, c) selenium elemental map. A single Se-filled SWCNT measuring around 1.5 nm in diameter is highlighted in the green dashed box.


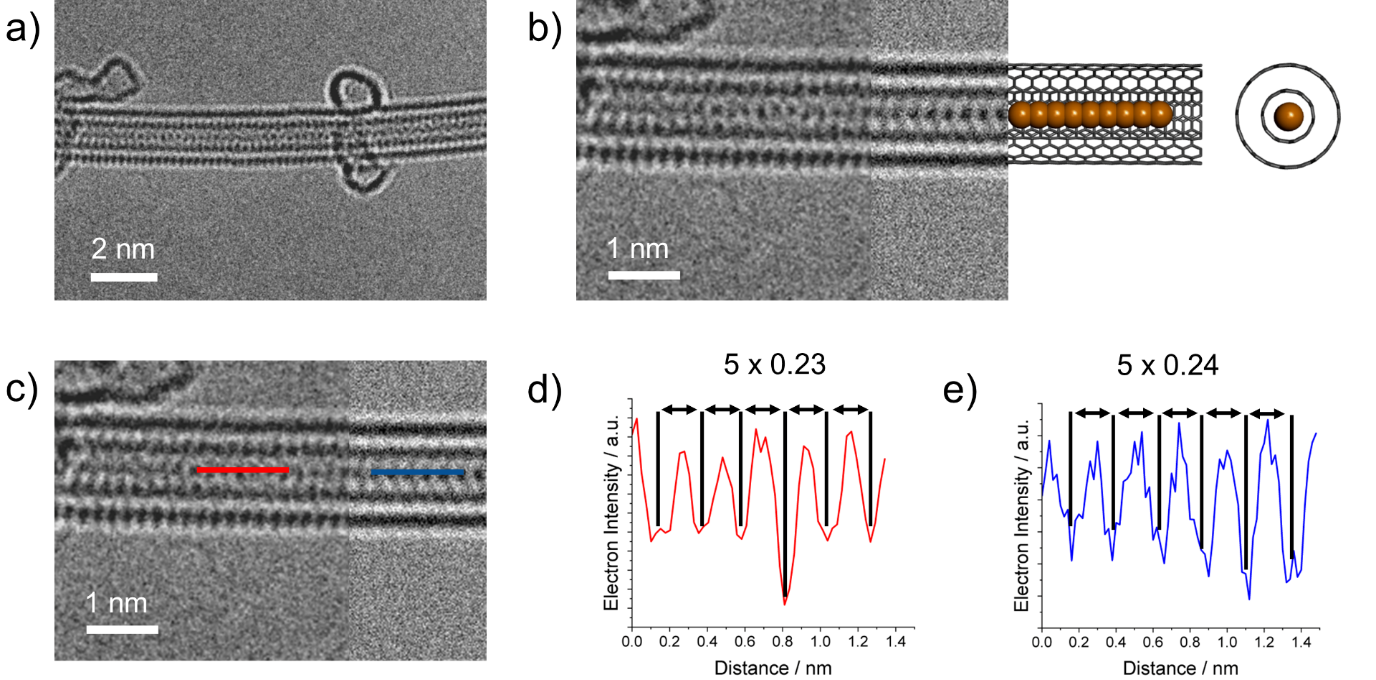


Figure S7. a) 60 kV AC-TEM image of Se@DWCNT, b) composite image of the AC-TEM image shown in b), including experimental AC-TEM image (left), simulated TEM image (centre), molecular model (right) and end-on view of the molecular model (far right), c) composite AC-TEM image of Se@Nanocyl 2100, from b), consisting of experimental AC-TEM image (left) and simulated TEM image (right), d) electron intensity profile map, generated from the red line superimposed over the experimental image in c), e) electron intensity profile map, generated from the blue line superimposed over the simulated image in c). The model of linear Se was created using the same Se-Se bond length as in t-Se (0.237 nm).^[9]^


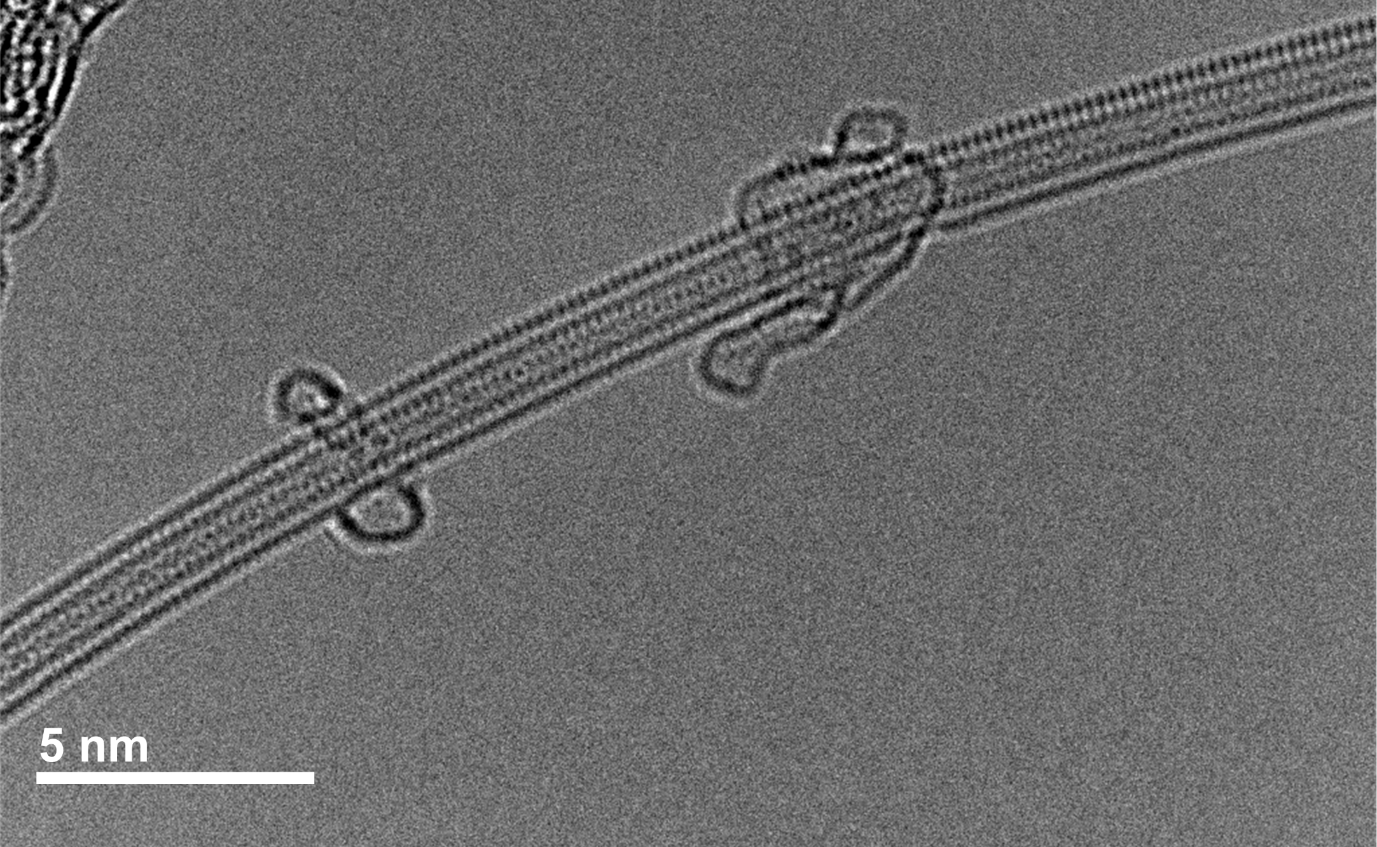


Figure S8. 60 kV AC-TEM image of Se@DWCNT, showing the longest length chain of l-Se imaged, measuring around 28 nm. The true maximum length of these chains could be far greater.


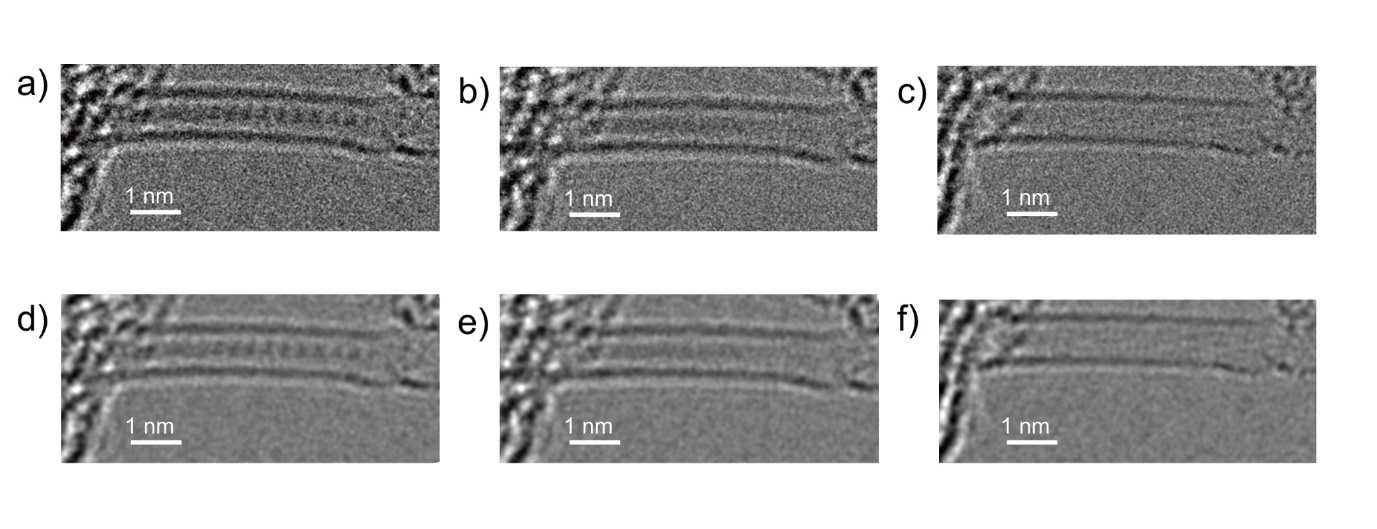


Figure S9. a) - f) 60 kV AC-TEM image series of a defect free Se@HiPCO SWCNT, with encapsulated Se freely translating and rotating inside the SWCNT cavity.


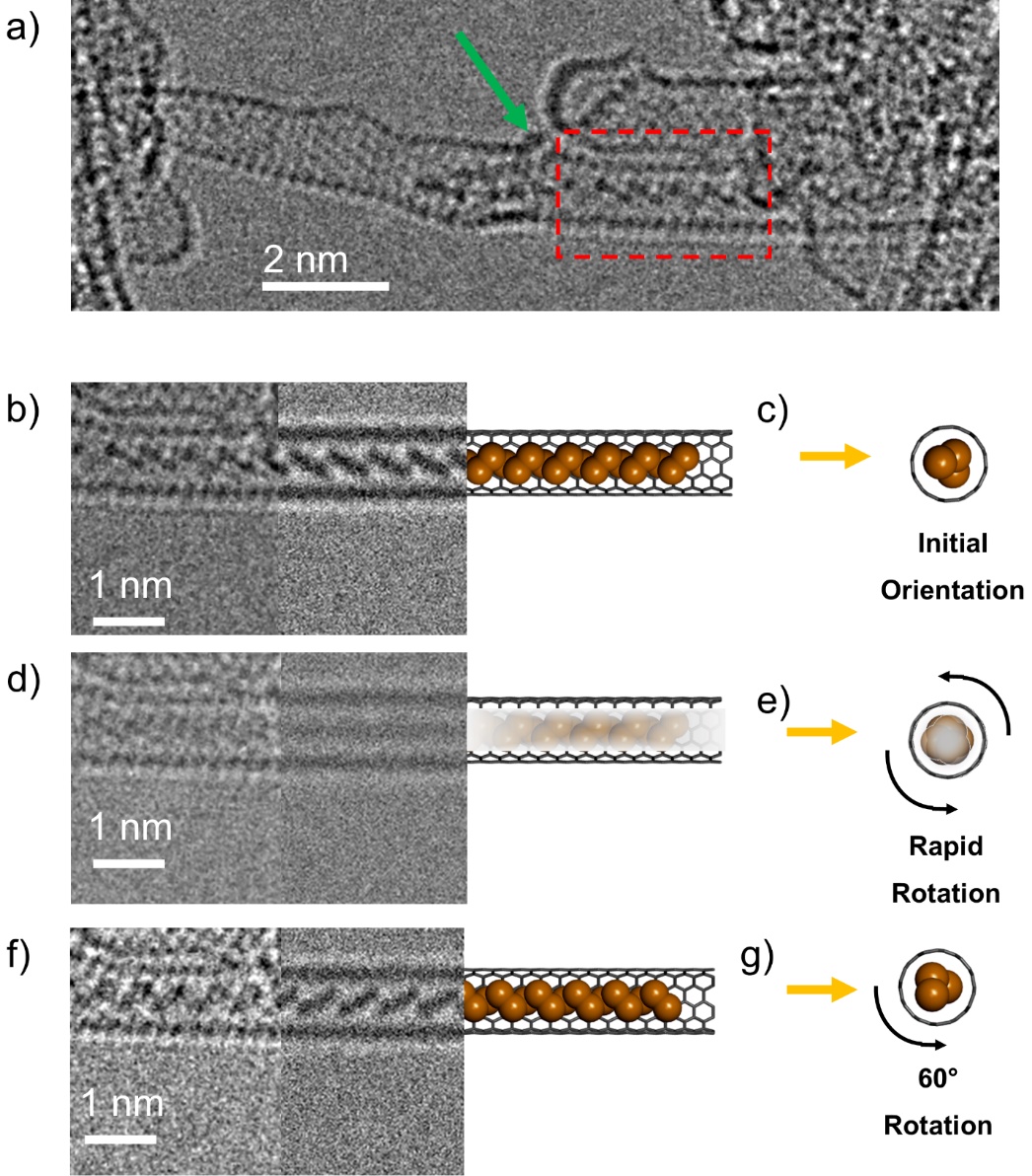


Figure S10. a) 60 kV AC-TEM image of Se@HiPCO SWCNTs, with a defect in the CNT wall designated by the green arrow, b), d) and f) shown composite AC-TEM images of the area highlighted by the red box in a) after continued imaging, where left is an experimental AC-TEM image, centre is a simulated AC-TEM image and right is a molecular model. c), e) and g) shown end-on projections of the models used in c), e) and g), respectively, and the degree they are rotated in the axis of the nanotube with respect to one another, with the yellow arrows representing the direction of the electron beam. Our measurements show that in order to correctly determine the exact atomic structure of a fast-moving Se chain it is important to restrain translation motion so that it is commensurate with the image capture rate and to image a range of projections of the same 3D object.


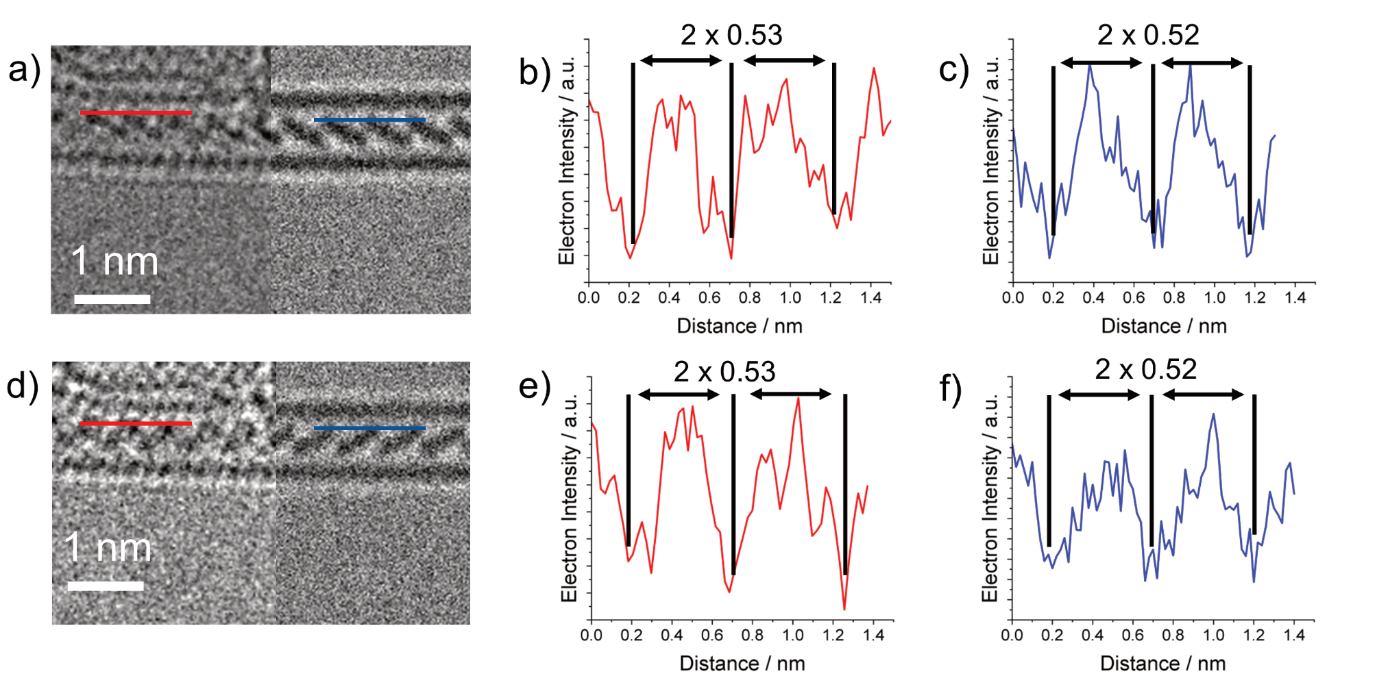


Figure S11. Two part composite image of an AC-TEM image (left) and a simulated TEM image (right) of a Se nanowire, b) and c) show electron density profile maps in red, generated from the red line superimposed over the experimental AC-TEM image in a), and in blue, generated from the blue line superimposed over the simulated TEM image in a), with calculated interatomic distances highlighted in nm. d), e) and f) show the same as a), b) and c), respectively, but for a second orientation of the Se NW.


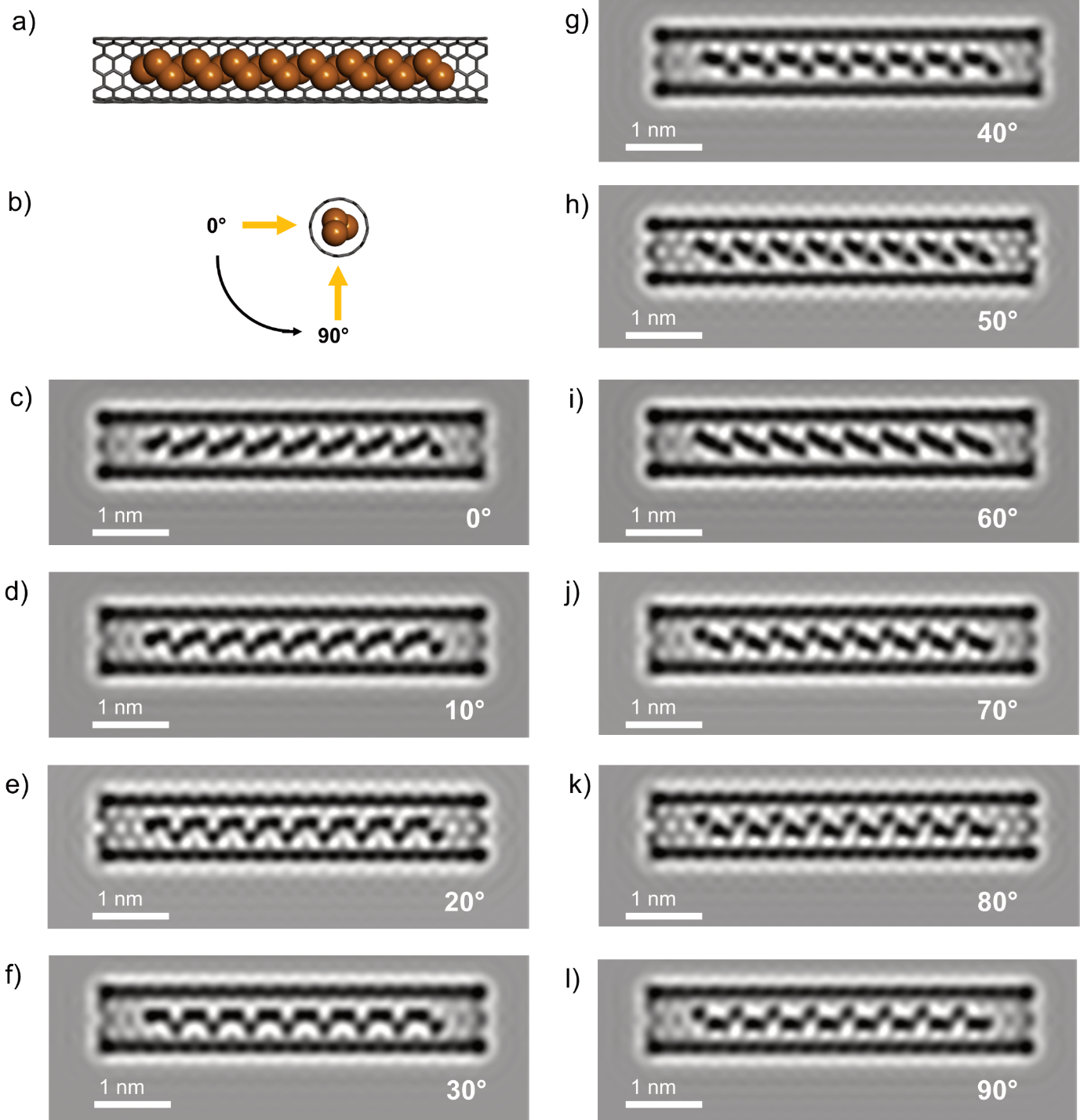


Figure S12. Rotational tableau showing 60 kV TEM simulations of a single t-Se nanowire encapsulated inside a (6,6) SWCNT. a) Molecular model used to create the simulations, with Se atoms in yellow/orange, b) end on projection of the model in a), with the orientation of the electron beam (yellow arrow) with respect to the model as it is rotated. *c) -* l) show a rotational series of simulated TEM images as the model shown in a), rotated in 10° steps.


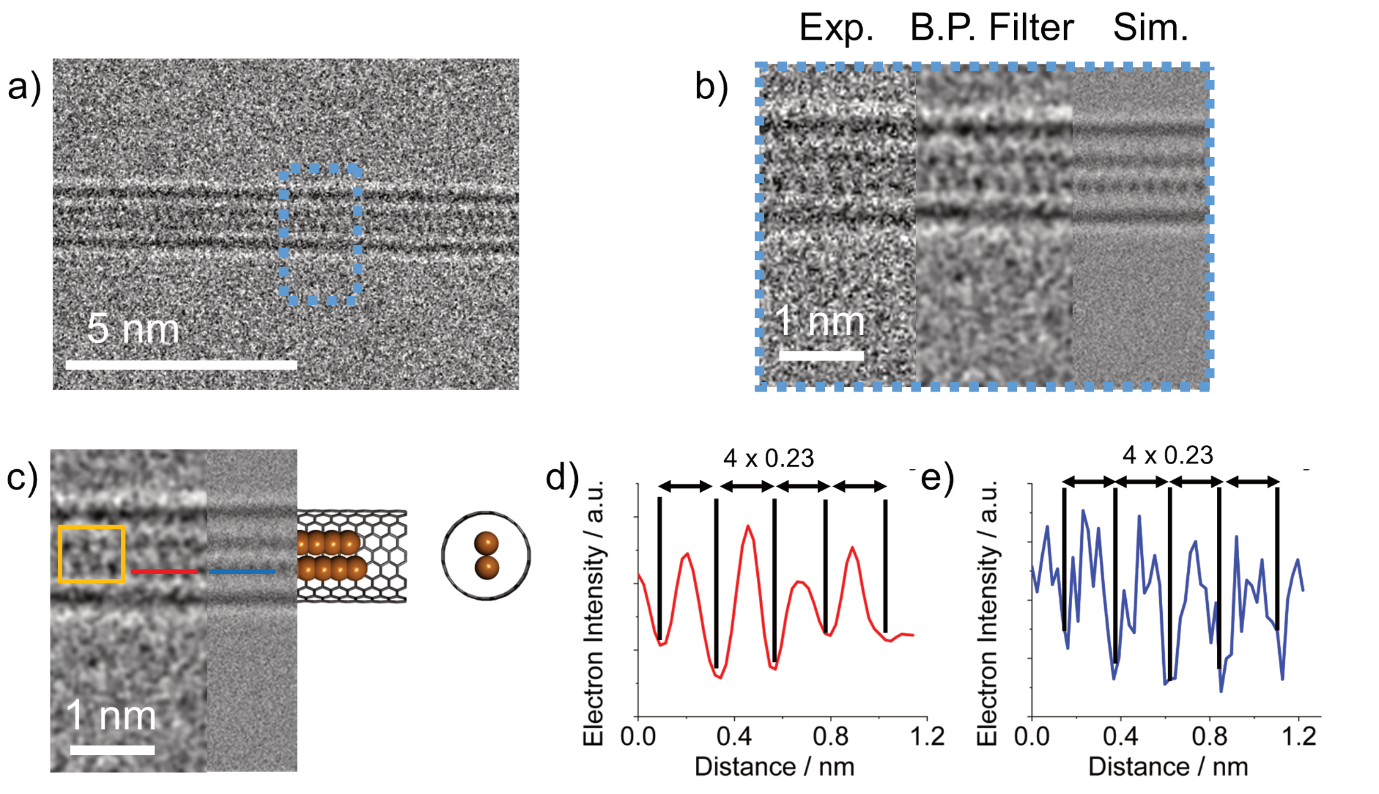


Figure S13. a) AC-TEM image of Se@HiPCO SWCNT, b) composite image of the blue dashed area in a), consisting of 60 kV experimental AC-TEM image (left), bandpass filtered AC-TEM image (centre) and simulated TEM image (right), c) Composite TEM image of Se@HiPCO SWCNT, consisting of bandpass filtered (smaller than 3 and larger than 40 pixels) experimental AC-TEM image (left), simulated image (centre) and molecular model (right). Also shows end on orientation of the molecular model (far right) and a yellow box highlighting the staggered conformation of the two Se atomic chains, d) electron intensity profile map, generated from the red line superimposed over the experimental image in c), e) electron intensity profile map, generated from the blue line superimposed over the simulated image in c.).


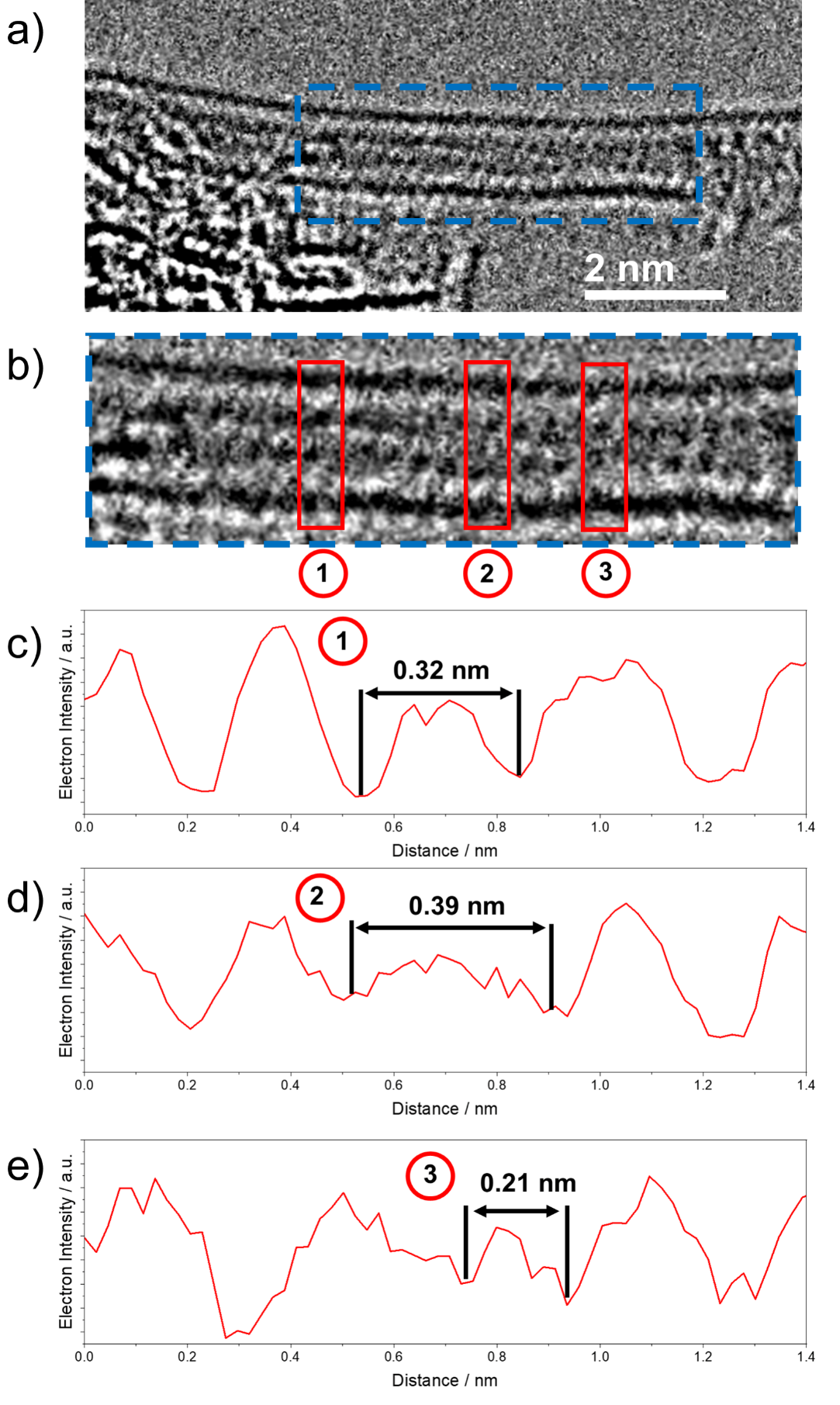


Figure S14. 60 kV AC-TEM image of two co-linear l-Se chains twisting inside a SWCNT. a) 60 kV AC-TEM image of two co-linear l-Se chains inside a CNT, b) digitally magnified AC-TEM image of the blue dished box area in the above image, c), d) and e) line profile analysis of the areas in the red boxes shown in b), corresponding to areas 1, 2 and 3 respectively. Line profile analysis confirms the two co-linear chains of l-Se twist around one another, as demonstrated by the changing projected distance between Se atoms.


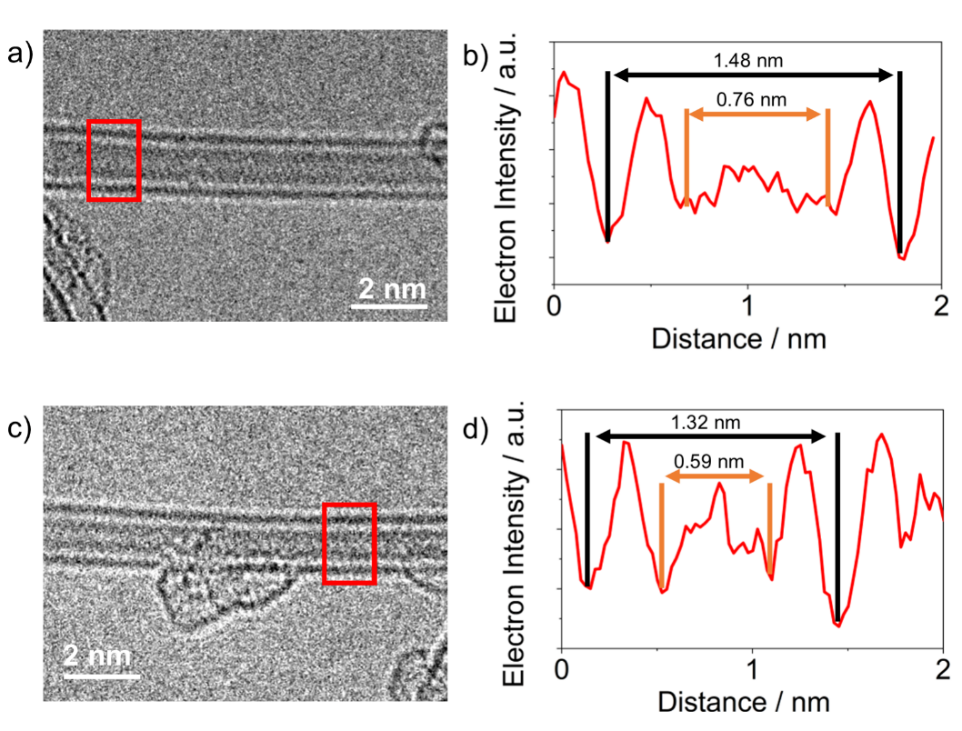


Figure S15: a) 60 kV AC-TEM image of Se@P2 SWCNT, b) electron intensity profile map generated from the red rectangle superimposed over the experimental image in a) with the diameter of encapsulated Se highlighted in orange and the SWCNT internal diameter highlighted in black.


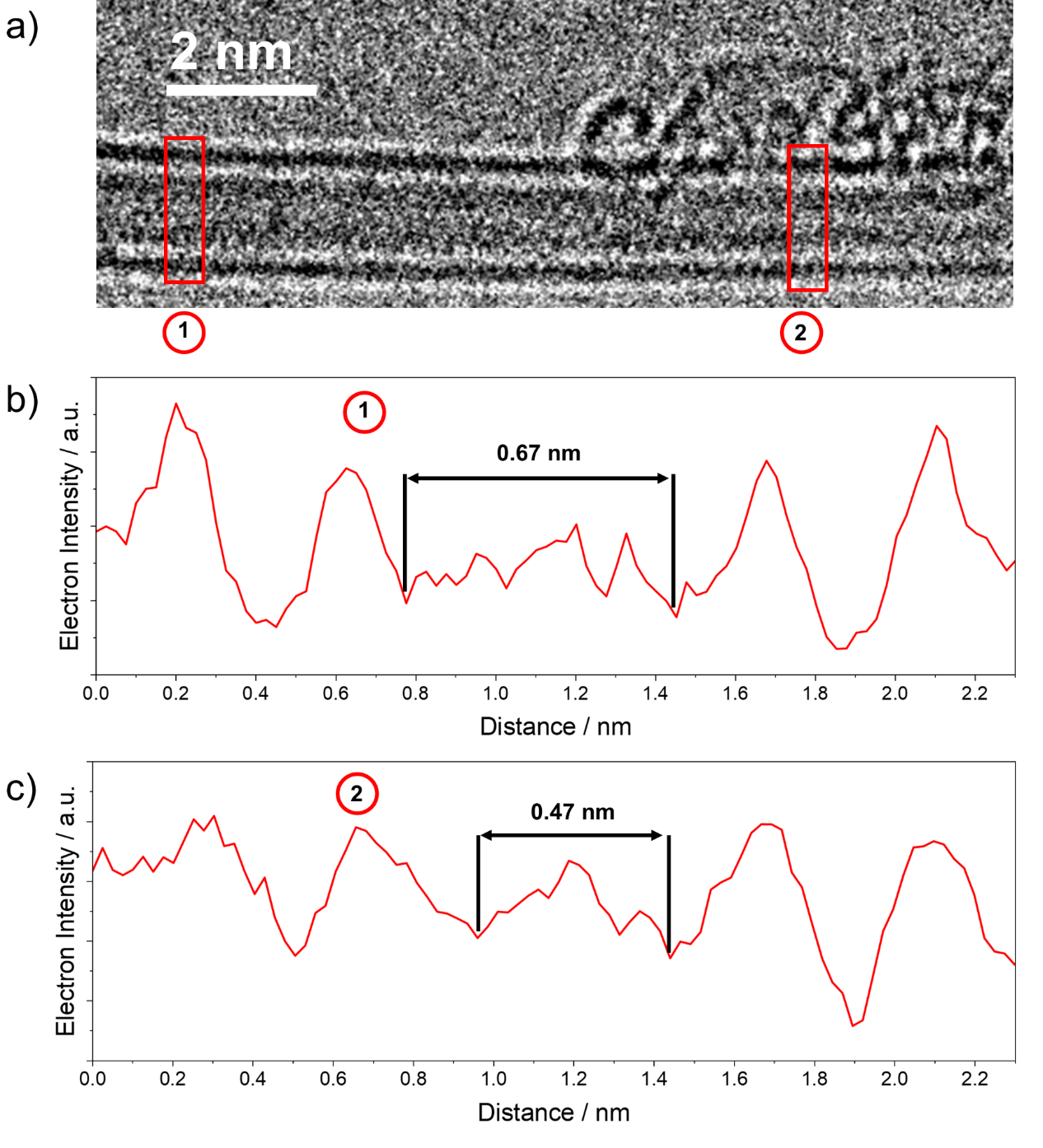


Figure S16. 60 kV AC-TEM image of two chains of t-Se, showing how the spacing between t-Se chains can decrease as a result of the two chains twisting inside the CNT. a) 60 kV AC-TEM image of two co-linear l-Se chains inside a CNT, b) and c) line profile analysis of the areas in the red boxes shown in a), corresponding to areas 1 and 2, respectively.


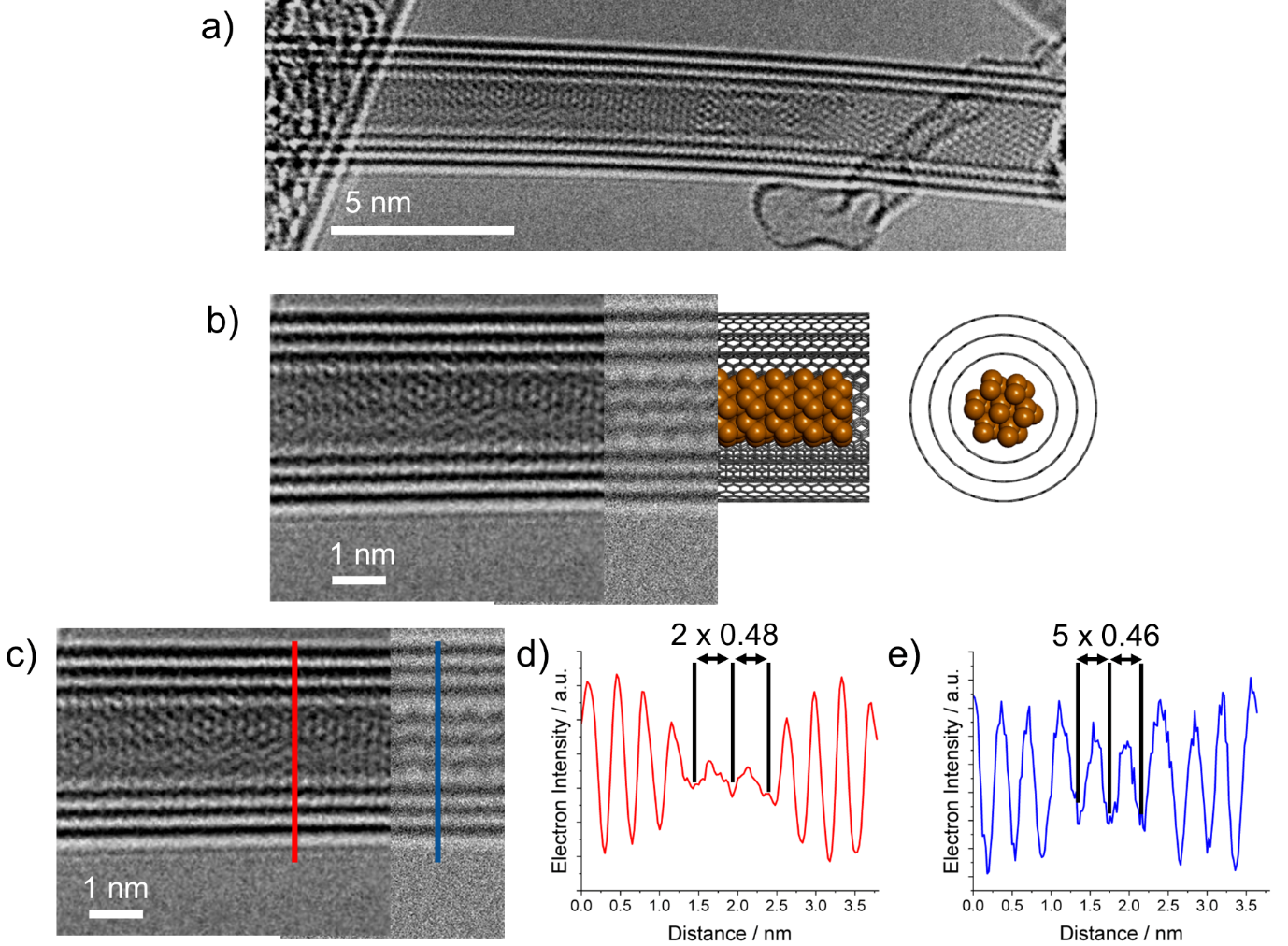


Figure S17: a) 60 kV AC-TEM image of multiple trigonal Se chains inside a triple wall CNT, composite image of the AC-TEM image shown in a), including experimental image (left), simulated image (centre) and side-on molecular model (right) and end-on view of the molecular model (far right) c) composite AC-TEM image of the image shown in a) and b) consisting of experimental AC-TEM image (left) and simulated TEM image (right), e) electron intensity profile map, generated from the red line superimposed over the experimental image in c), f) electron intensity profile map, generated from the blue line superimposed over the simulated image in c). The encapsulated Se seen in Figure S17a had a similar structure to that of seven chains of t-Se, rotated 10° in the axis of the nanotube. When TEM simulations of this structure were made, a slightly larger interchain spacing in projection was recorded when compared to bulk t-Se due to the 10° rotation (0.46 nm at 10° vs 0.44 nm at 0°). Comparing the simulated TEM image to the experimental TEM image, significantly less contrast is seen in spaces in between t-Se chains. This is attributed to the high mobility of t-Se chains with respect to one another, leading to a ’smearing’ of contrast. This independent movement of t-Se chains could not be replicated in simulated images, leading to a slight discrepancy in appearance.


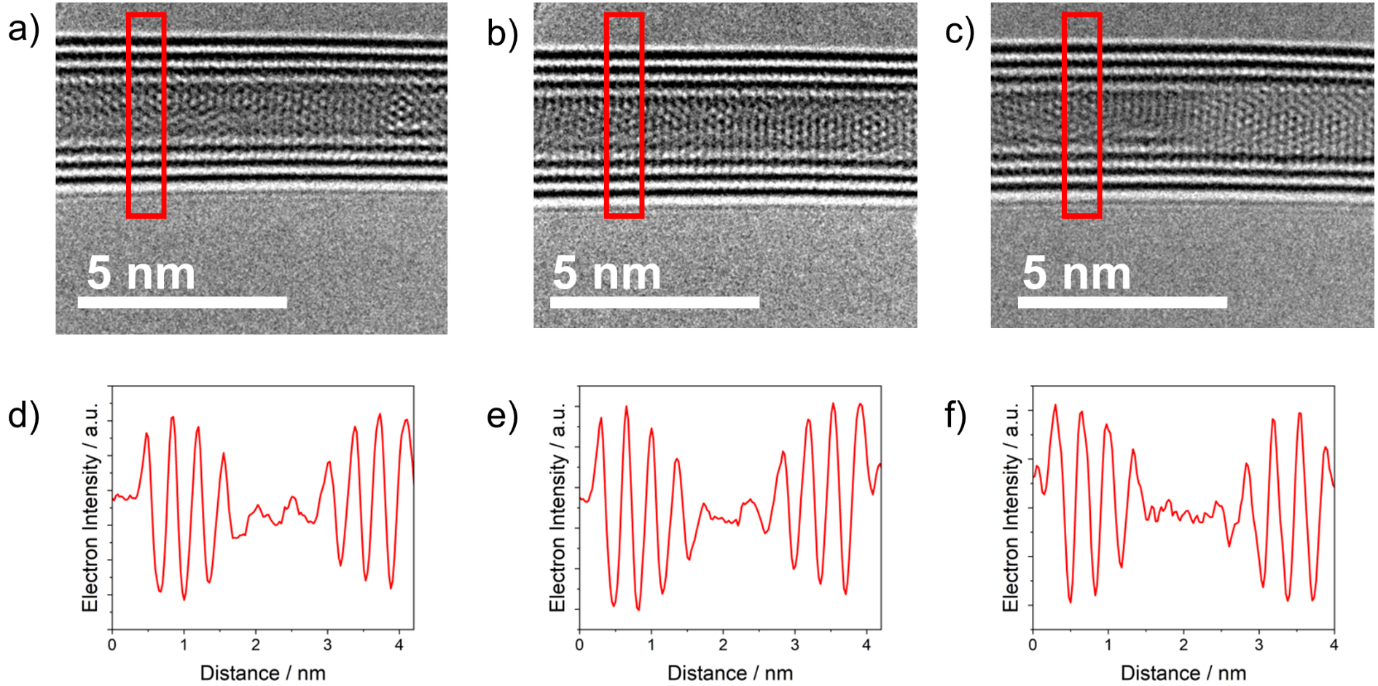


Figure S18. AC-TEM images and line profile analysis of the Se structure are shown in Figure S17, highlighting its mobility. a), b) and c) 60 kV AC-TEM images of t-Se@CNT, d), e) and f), line profile analysis of the areas highlighted in the red box of a), b) and c), respectively.


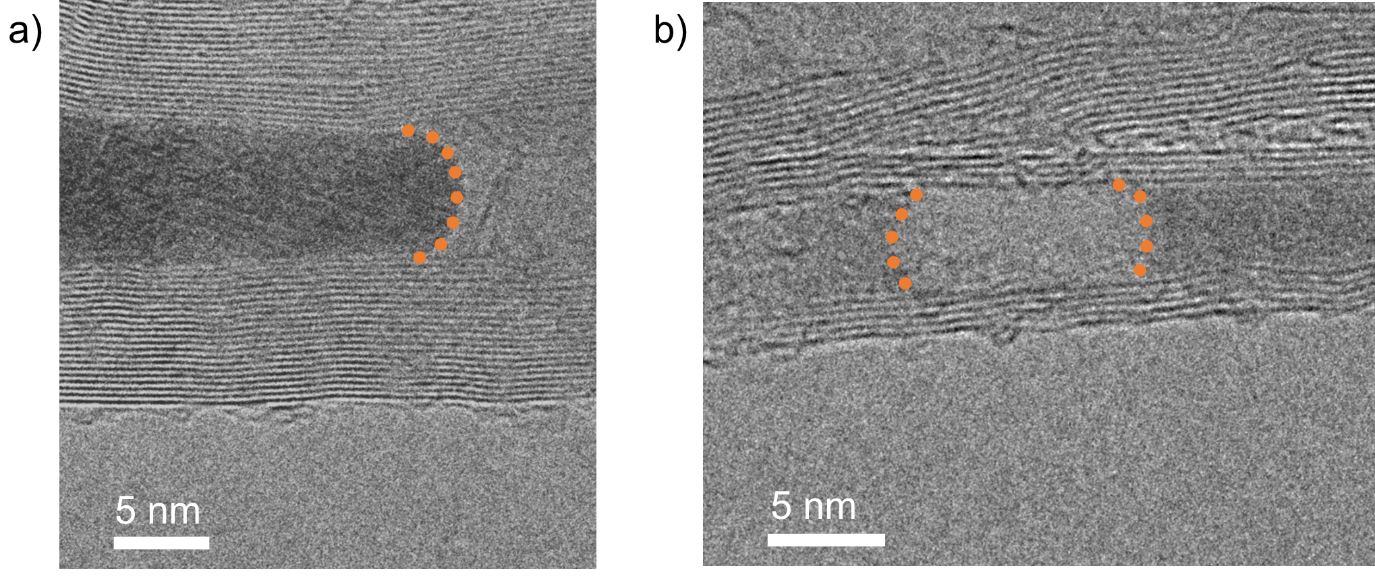


Figure S19: a) 200 kV TEM image of Se@PD30 MWCNTs, showing a convex meniscus (orange dotted line), b) 200 kV TEM image of Se@PD30 MWCNTs, showing two concave menisci (orange dotted line).


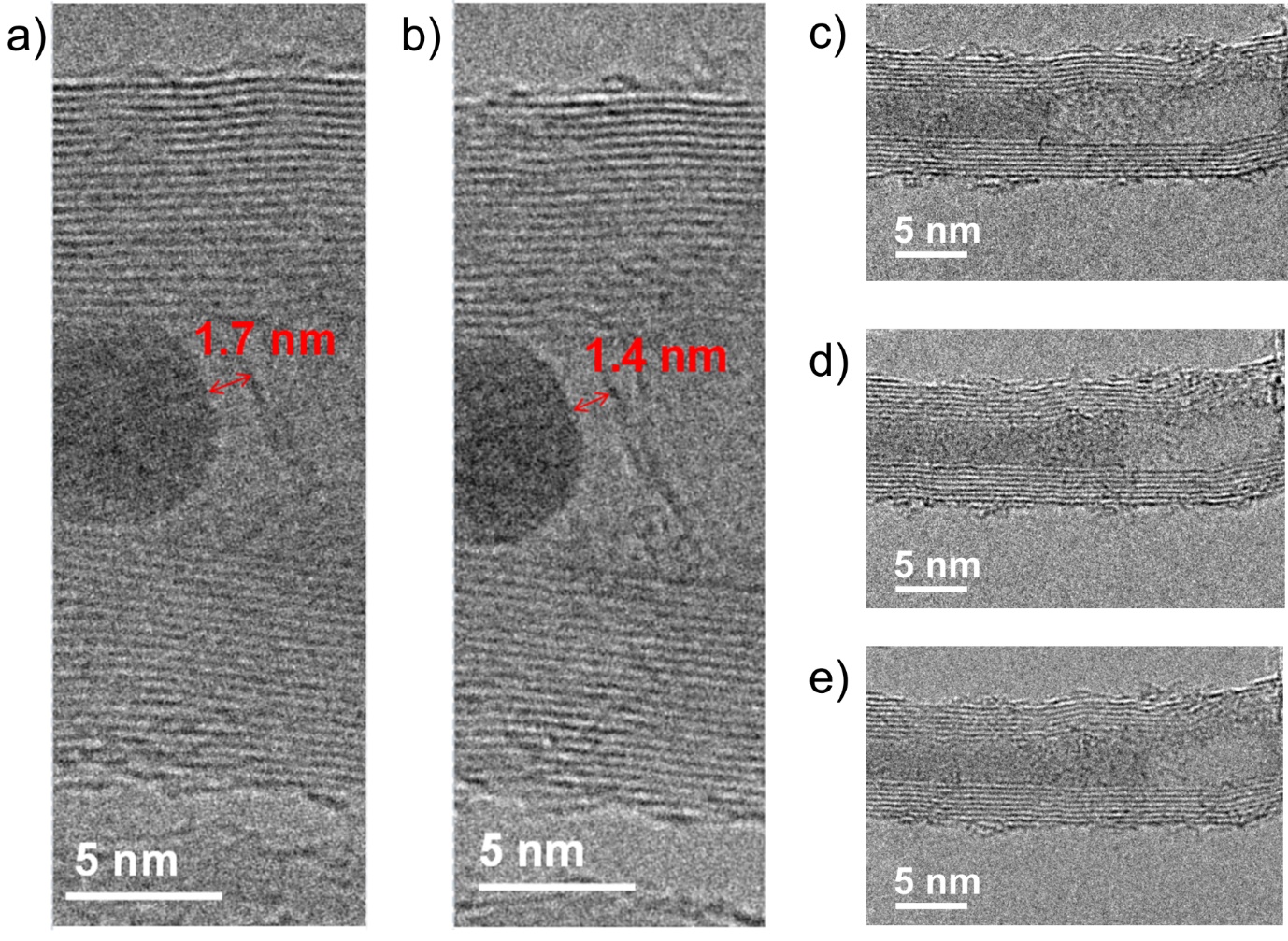


Figure S20. TEM images showing the mobility of Se encapsulated by larger diameter CNTs, showing the mobility of the encapsulated Se. a) and b) show how the encapsulated Se shown in Figure S19a moves along the internal cavity of the CNT during continued image acquisition. c), d) and e) show the same translational motion but for Se encapsulated by a smaller diameter CNT, imaged at 200 kV.


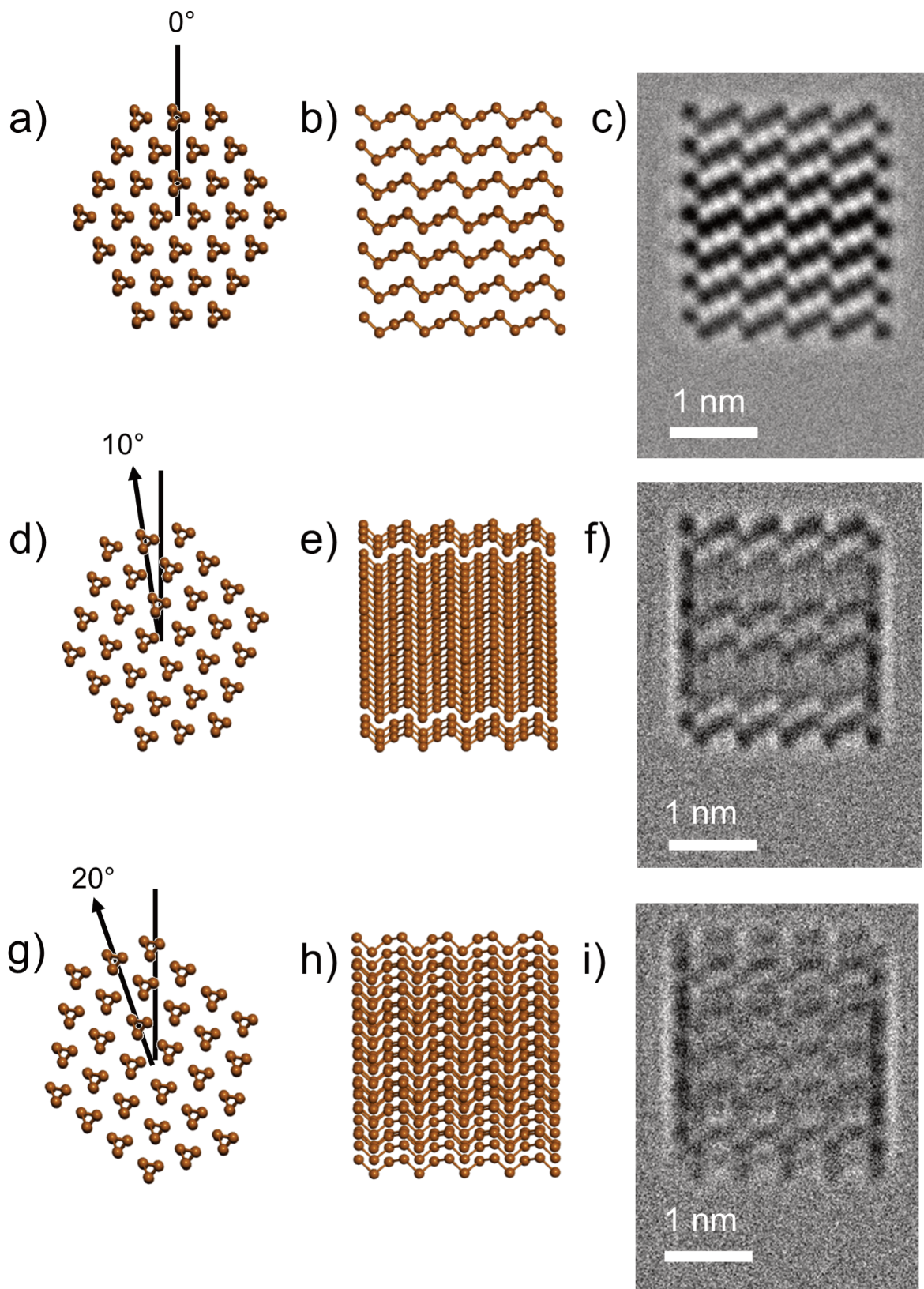


Figure S21: Structural diagrams in a), b) and c) show an end-on projection, side-on projection and TEM simulation of side-on projection of a bundle of t-Se chains, respectively, d), e) and f) and g), h) and i) show the same but for a 10° and 20° rotation of the t-Se bundle, respectively. Orange/yellow atoms are selenium. Defined spacings corresponding to t-Se appear more faintly as the bundle is rotated off-axis, as has been previously for other nanoscale materials.^[10]^

Table S5: Dimensions of Se and CNTs used for Figure 2m.

| **Figure in main text** | **Conformation of Se** | **Measured CNT diameter / nm** | **Simulated Se vdW diameter / nm** | **Type of NT used for encapsulation** |
| --- | --- | --- | --- | --- |
| 2d | Linear chain | 0.73 | 0.38 | Nanocyl 2100 |
| 2e | Single t-Se chain | 0.88 | 0.53 | HiPCO SWCNT |
| 2f | Co-linear Se chains | 1.1 | 0.76 | HiPCO SWCNT |
| 2g | Two t-Se chains | 1.32 | 0.97 | P2 SWCNT |
| 2h | Two t-Se chains | 1.48 | 1.14 | P2 SWCNT |
| 2i | Seven t-Se chains | 1.78 | 1.4 | Nanocyl 2100 |
| 2j | >10 t-Se chains | 4.38 | 4.02 | PD30 MWCNT |

Table S6: Dimensions of Se and CNTs used in Figure 2n

| **Conformation of Se** | **vdW diameter / nm** | **Experimentally recorded encapsulating CNT diameter / nm** | **Smallest possible encapsulating CNT diameter / nm** |
| --- | --- | --- | --- |
| Linear chain | 0.38 | 0.73 | 0.72 |
| Single t-Se chain | 0.53 | 0.88 | 0.87 |
| Co-linear Se chains | 0.76 | 1.10 | 1.10 |
| Two t-Se chains | 0.97 | 1.32 | 1.31 |


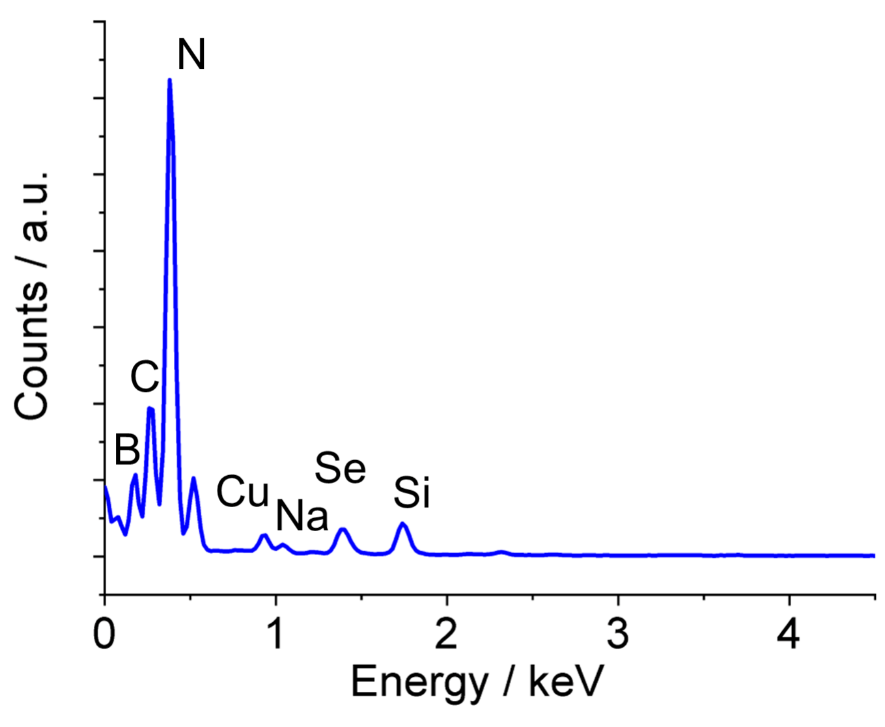


Figure S22: EDX analysis of Se@BNNT. Na and Si are trace contaminants from the pyrex ampoules used in synthesis. C and Cu are from the lacey carbon on Cu TEM grid.


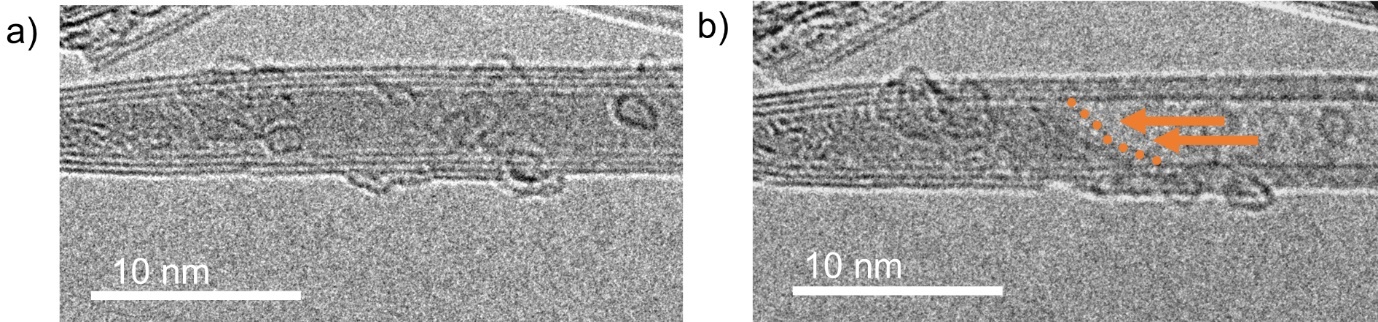


Figure S23: a) and b) 200 kV TEM images of the same Se-filled BNNT after continued imaging, showing the mobility of encapsulated Se (orange annotations in b)).


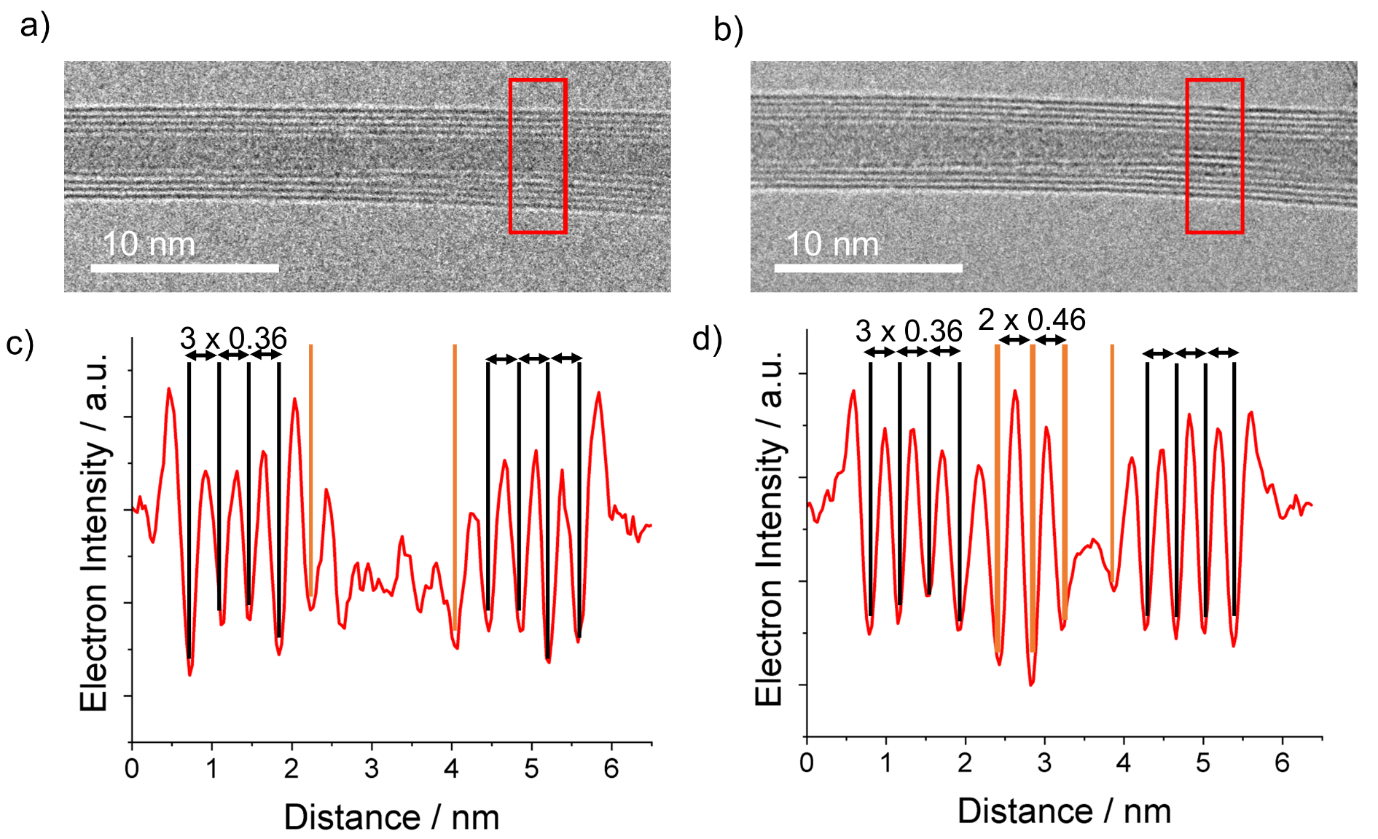


Figure S24: a) and b) show 200 kV TEM images of Se@BNNT before and after electron beam irradiation. c) and d) show electron intensity profile maps, generated from the area highlighted in the red rectangle in a) and b) respectively.

Video S1: AC-HRTEM image series of Se@BNNT


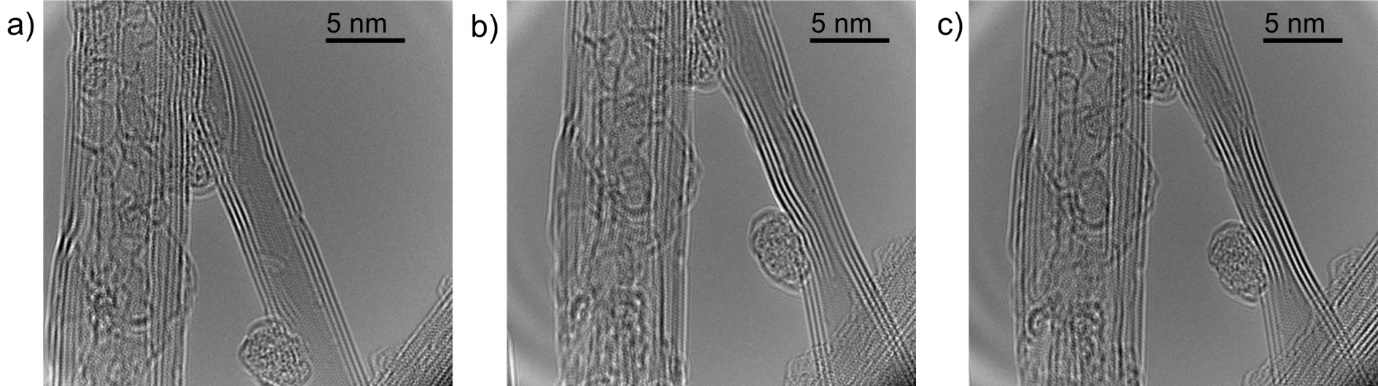


Figure S25. a), b) and c) un-cropped 80 kV AC-TEM images of the BNNT shown in Figures 4a, b and c.

Video S2. HR-TEM irradiation of Se@BNNT


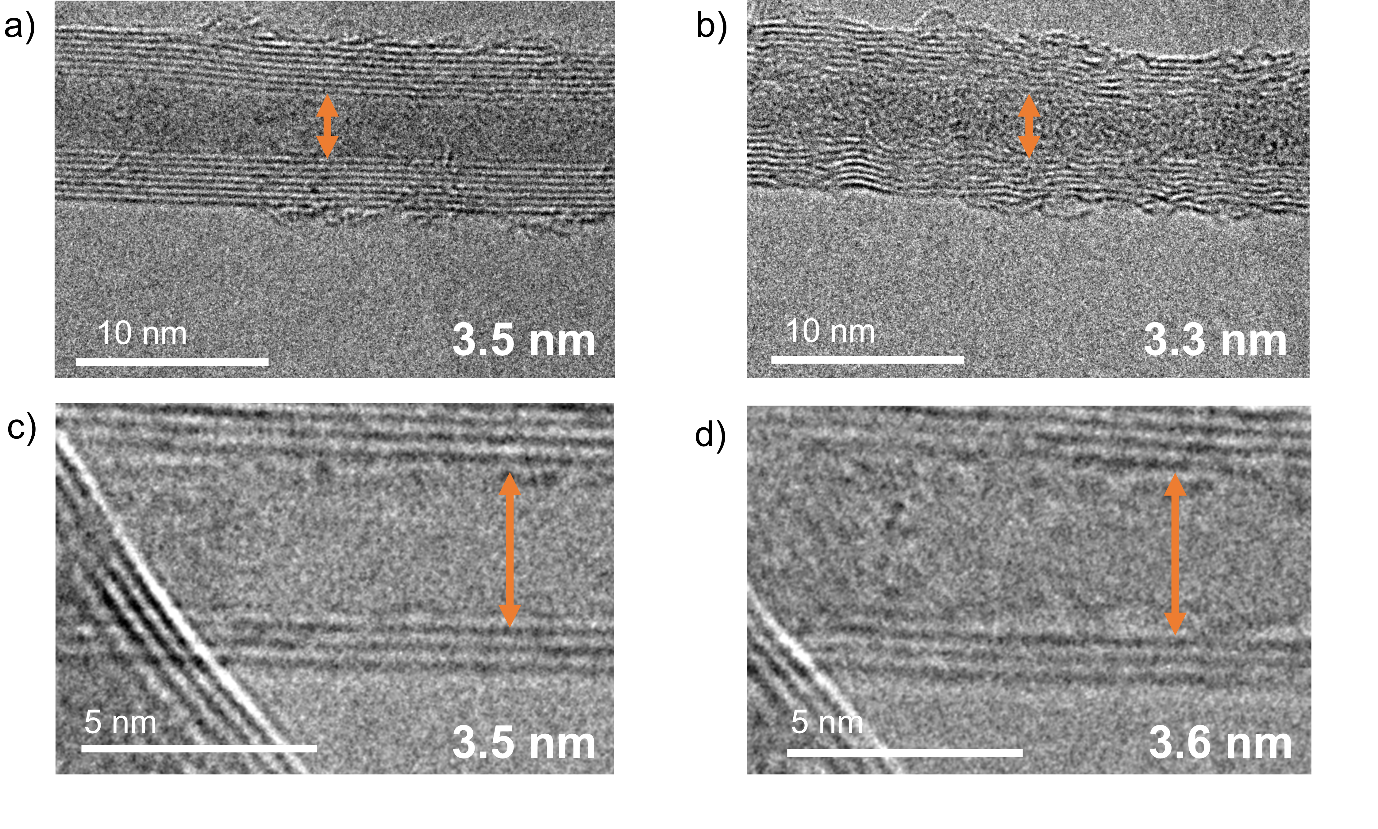


Figure S26. a) and b) show 200 kV TEM images of Se@PD30 MWCNT in it’s initial and final state (i.e. before and after 25 × 10^8^ e^-^ nm^-2^ of irradiation), c) and d) shows the same but for an empty BNNT. Measured internal diameters are shown in the bottom right of each TEM image in white.


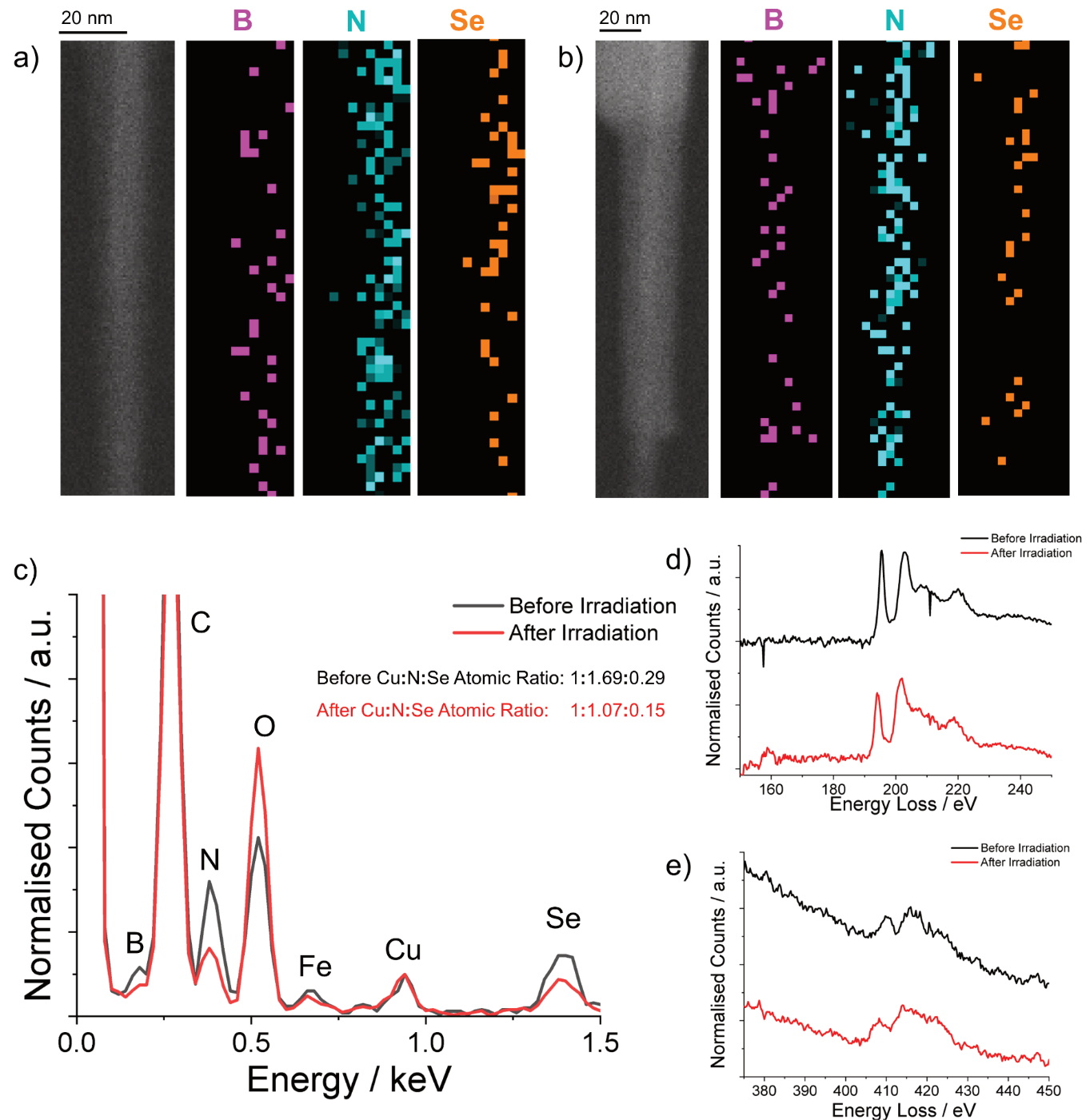


Figure S27: STEM-EDX analysis of a bundle of Se@BNNT before irradiation, showing dark field 200 kV STEM image (left), B elemental map (centre left), N elemental map (centre right), Se elemental map (right), b) shows the same as a) but for the same area after electron beam irradiation, c) shows quantitative EDX analysis of the areas in a) and b) before (black line) and after (red line) irradiation, normalised to the counts for Cu, d) shows the B K-edge of the bundle of Se@BNNT before (black) and after (red) irradiation, e) shows the N K-edge of the bundle of Se@BNNT before (black) and after (red) irradiation. EELS spectra were baseline corrected in Gatan digital micrograph using a second order power law fit. The BNNT bundle was irradiated with an electron fluence of 7.28 ×10^8^ e^-^ nm^-2^ at 200 kV. The fluence to which the sample was exposed during STEM analysis was not accounted for in this calculation, meaning the true fluence value is likely higher than this. Following irradiation B, N and Se concentrations are shown to decrease, indicating that the extrusion process seen in Figure 4 is also occurring here.


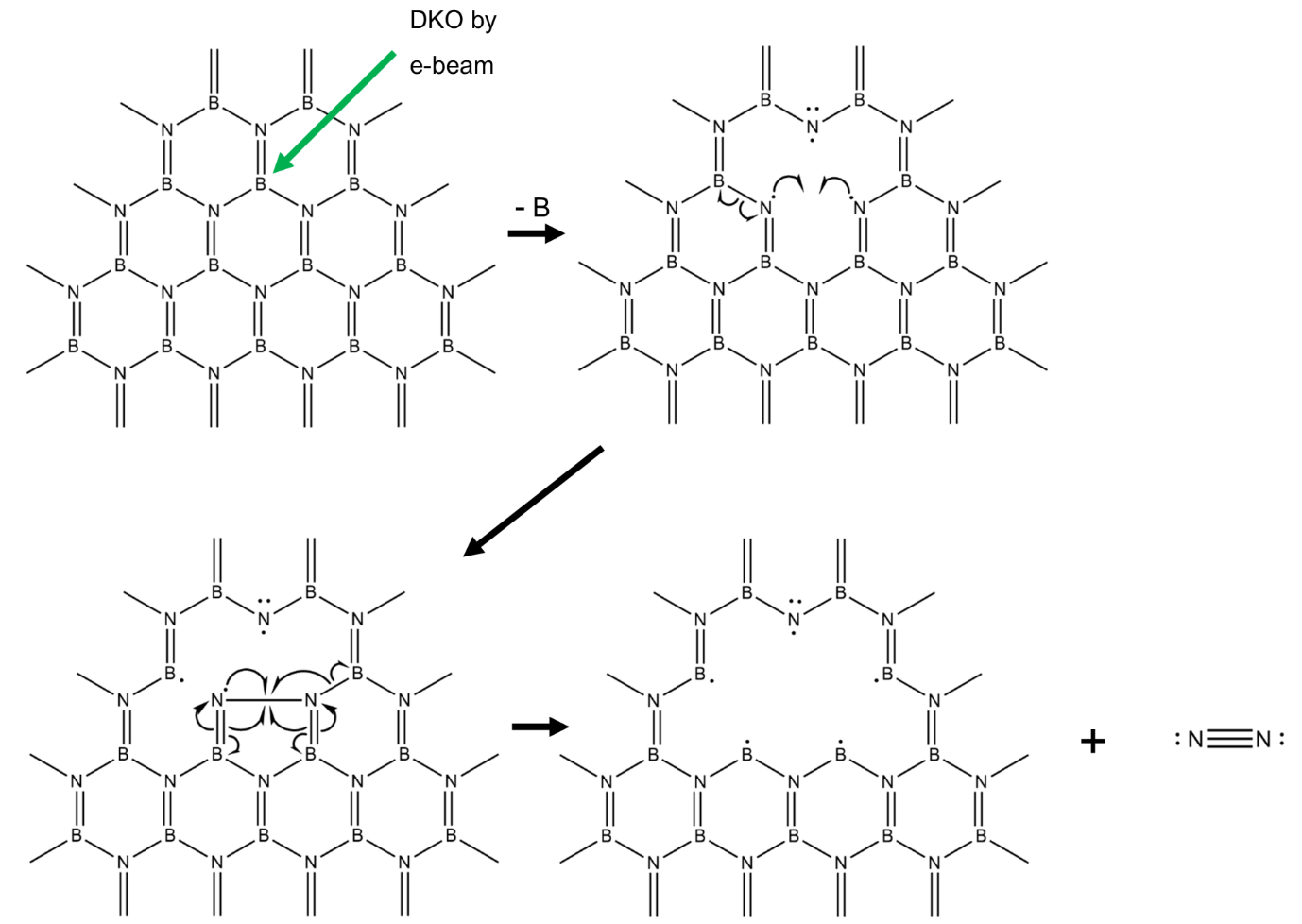


Figure S28: Schematic of one possible route of e-beam defect formation in BNNT (80 kV). In the first step, the electron beam causes the ejection of a B atom by DKO. The accelerating voltage threshold that allows for DKO of atoms in a BNNT has been reported as 79.5 kV for B and 118.6 kV for N. Following this, N_2_ can be eliminated, leaving a defect site, bordered with radicals. This is drastically different from graphene-based structures (i.e., as shown in Figure S30), where bonding between adjacent radicals causes stabilisation rather than further growth at the defect site. As shown by Kotakoski *et al.*, Nitrogen radicals adjacent to boron vacancies (such as those depicted in the second step of the above reaction) can also be removed by direct knock-on at 80 kV, providing another route to the growth of the defect site.^[11]^


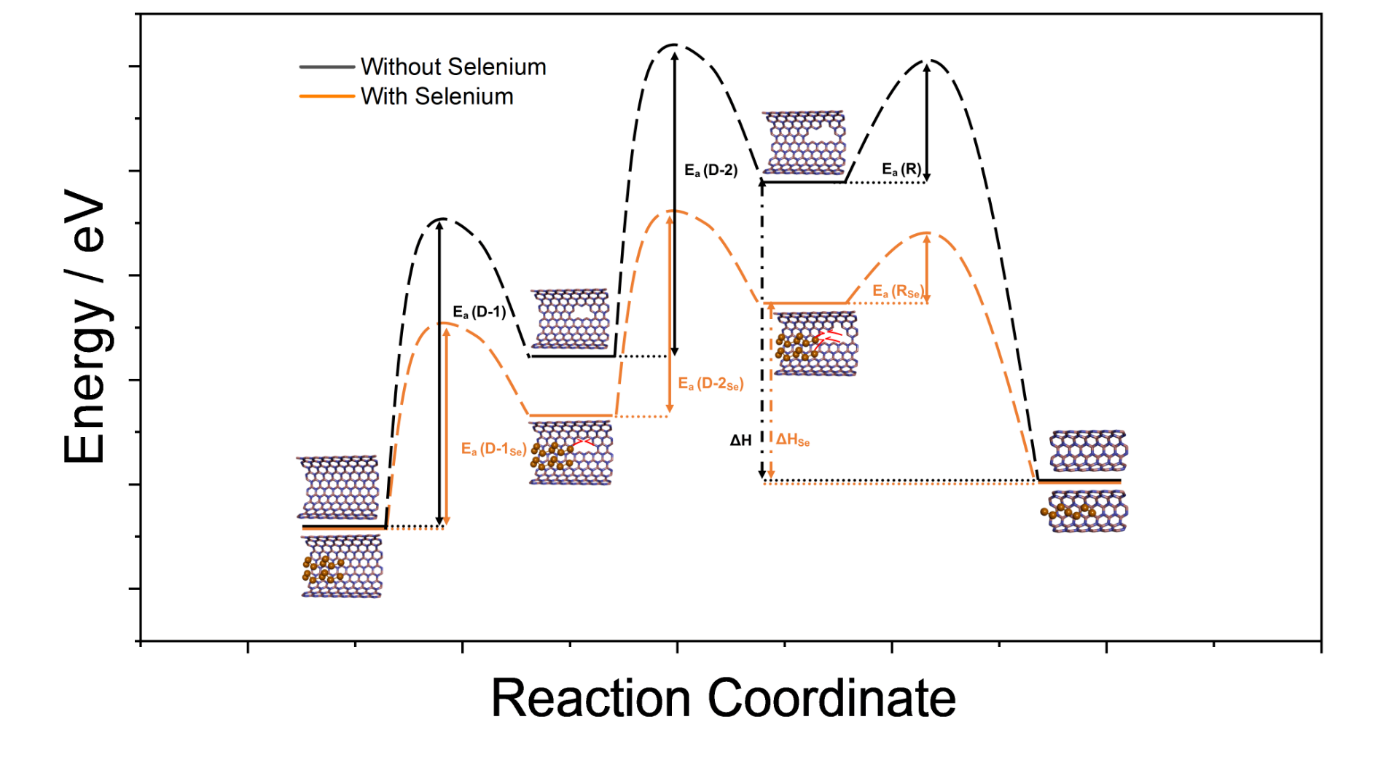


Figure S29: Reaction coordinate diagram showing how encapsulated Se catalyses the electron-beam-induced shrinkage of BNNT (reported previously by Celik-Akas *et al*.^[12]^ and Cheng *et al.*^[13]^). This reaction has two main parts: i) the formation of defects in the BNNT sidewall due to direct knock-on, the activation energy of which is represented by E_a_(D-1) and E_a_(D-2), and ii) the rearrangement reaction that causes the BNNT to shrink when a defect becomes large enough, the activation energy of which is represented by E_a_(R). The presence of t-Se chains in the internal cavity of the BNNT allows for the coordination between the termini of t-Se chains and the defect sites formed during e-beam irradiation. As per the Bell Evans Polyani principle, the difference in activation energy for reactions of a similar type is proportional to the difference in enthalpy of their transition states. As coordination between t-Se chains and BNNT defect sites lowers the enthalpy of transition states, activation energies will lower in turn, resulting in a lower activation energy for both the formation of defects in the Se@BNNT sidewalls (E_a_(D-1_Se_) and E_a_(D-2_Se_) and the rearrangement reaction that causes the BNNT to shrink and Se to be extruded (E_a_(R_Se_)). It follows that E_a_(D-1_Se_) < E_a_(D-1), E_a_(D-2_Se_) < E_a_(D-2) and Ea(RSe) < Ea(R), affording an explanation for the increased rate of BNNT shrinkage when Se is internally encapsulated.


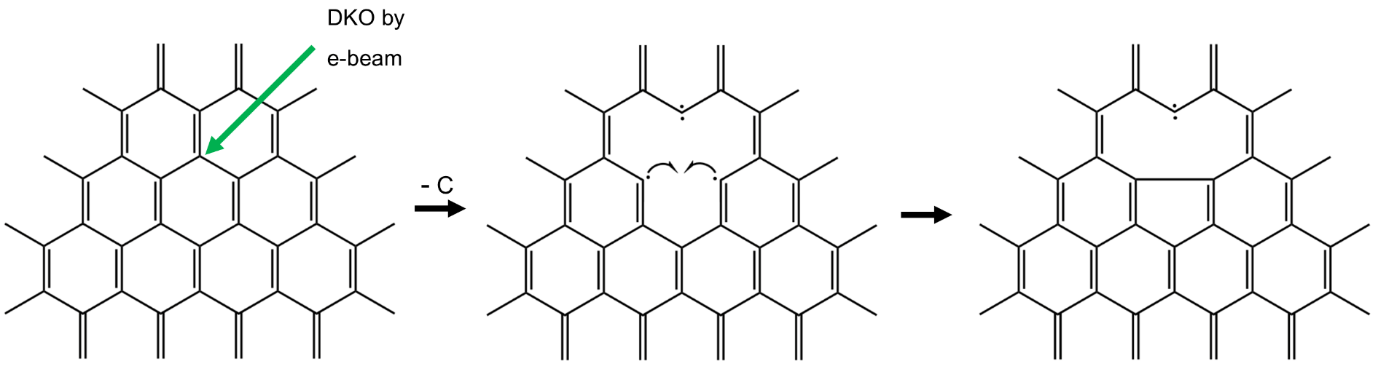


Figure S30: Schematic of e-beam defect formation in CNTs/Graphene (200 kV). Following the DKO of a carbon atom form the hexagonal lattice of the CNT. This damage mechanism has previously been studied in graphene by Meyer *et al.^[14]^* and also occurs in CNTs due to the same extended sp^2^ carbon lattice. Unlike in hBN, bonds can be formed between neighbouring atoms, which stabilise the defect centre, reducing the rate of CNT collapse compared to BNNTs.


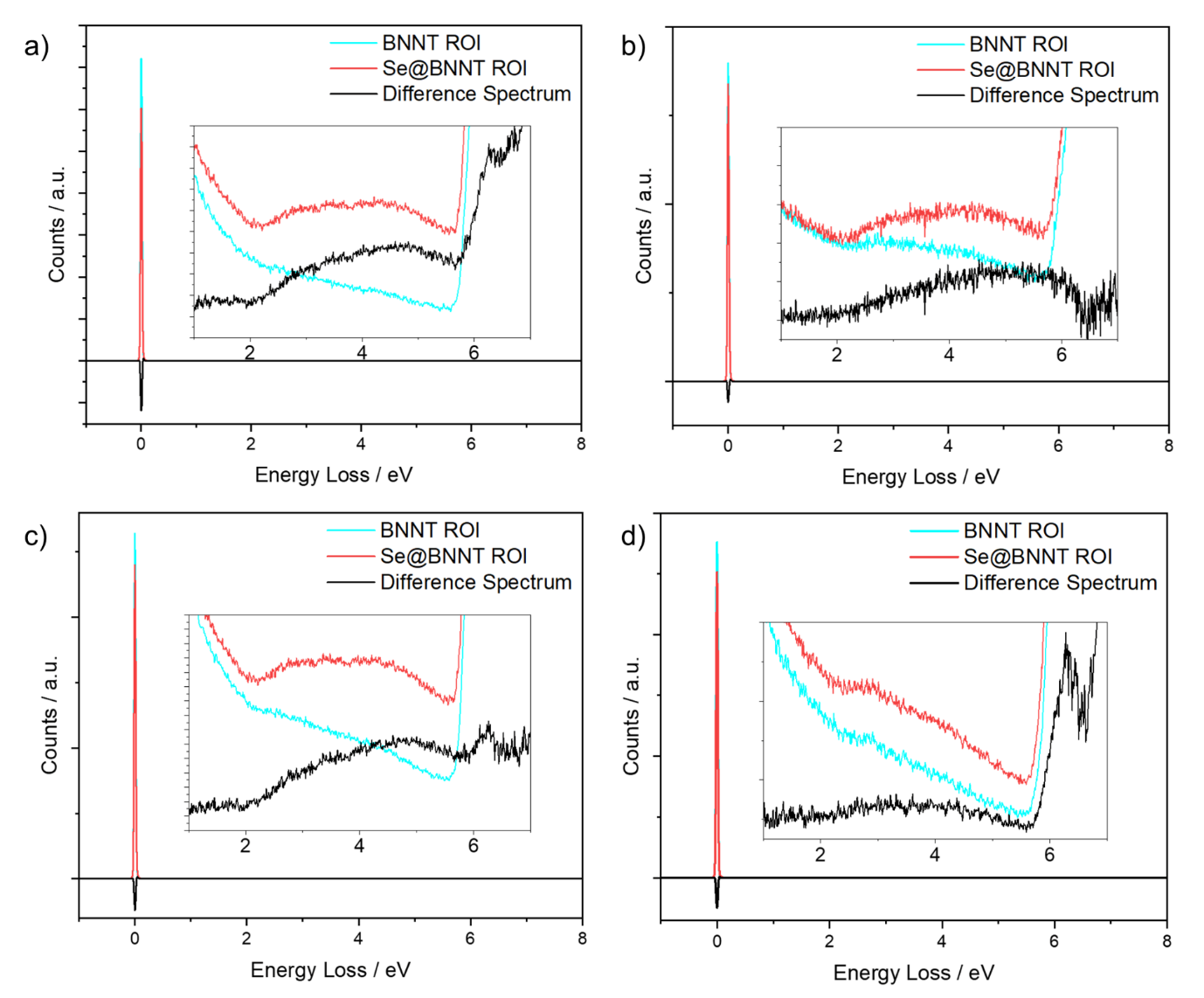


Figure S31: a), b), c) and d) raw EELS data corresponding to the difference spectra fits in Figures 5b, d, f and h, respectivley. In the case of a) b) and c), the zero loss peak (ZLP) is less intense for the Se@BNNT ROI (red) compared to the BNNT ROI (cyan). This is due to the Se@BNNT ROI having a greater thickness than the corresponding BNNT ROI, and therefore more scattering of the incedent ebeam occurs.

Table S7: The values from figure 5i

| **Corresponding Image** | **Measured Se Diameter / nm** | **Optical Band Gap / eV** | **Positive Error in Optical Band Gap / eV** | **Negative Error in Optical Band Gap / eV** |
| --- | --- | --- | --- | --- |
| 5a | 2.8 | 2.30 | +0.04652 | -0.07348 |
| 5c | 1.8 | 2.45 | +0.09961 | -0.15039 |
| 5e | 1.3 | 2.33 | +0.16 | -0.2 |
| 5f | 0.8 | 2.24 | +0.04652 | -0.07348 |


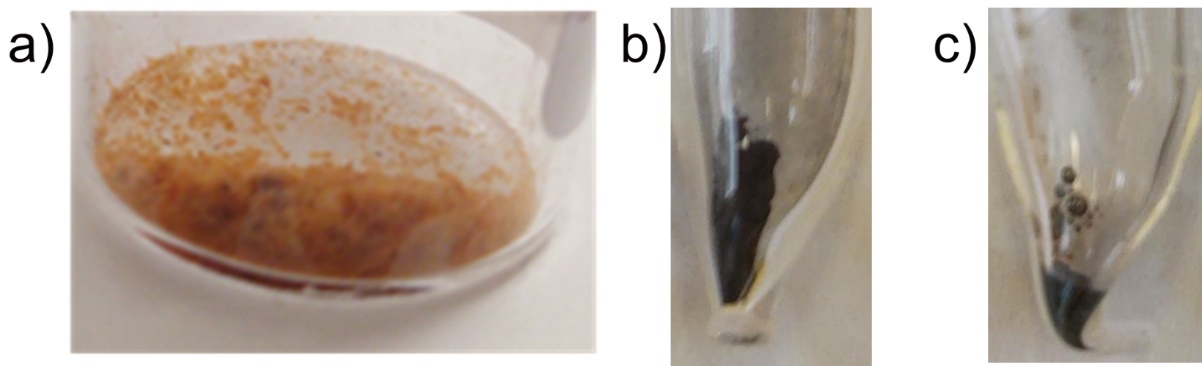


Figure S32: Photos of a) Se@BNNT, b) Se powder before heating to 550 °C at a pressure of 10^-5^ mbar and c) Se powder after heating to 550 °C at a pressure of 10^-5^ mbar. After encapsulating Se inside BNNTs the filled BNNT powders turned from white to orange. The Se powder used to fill these nanotubes is black/grey, and when exposed to the same reaction conditions with no BNNT present, no colour change occurred. This implies that encapsulation of Se inside BNNTs causes a change in the optical properties of Se due to nanoconfinement effects, rather than a thermal transformation.


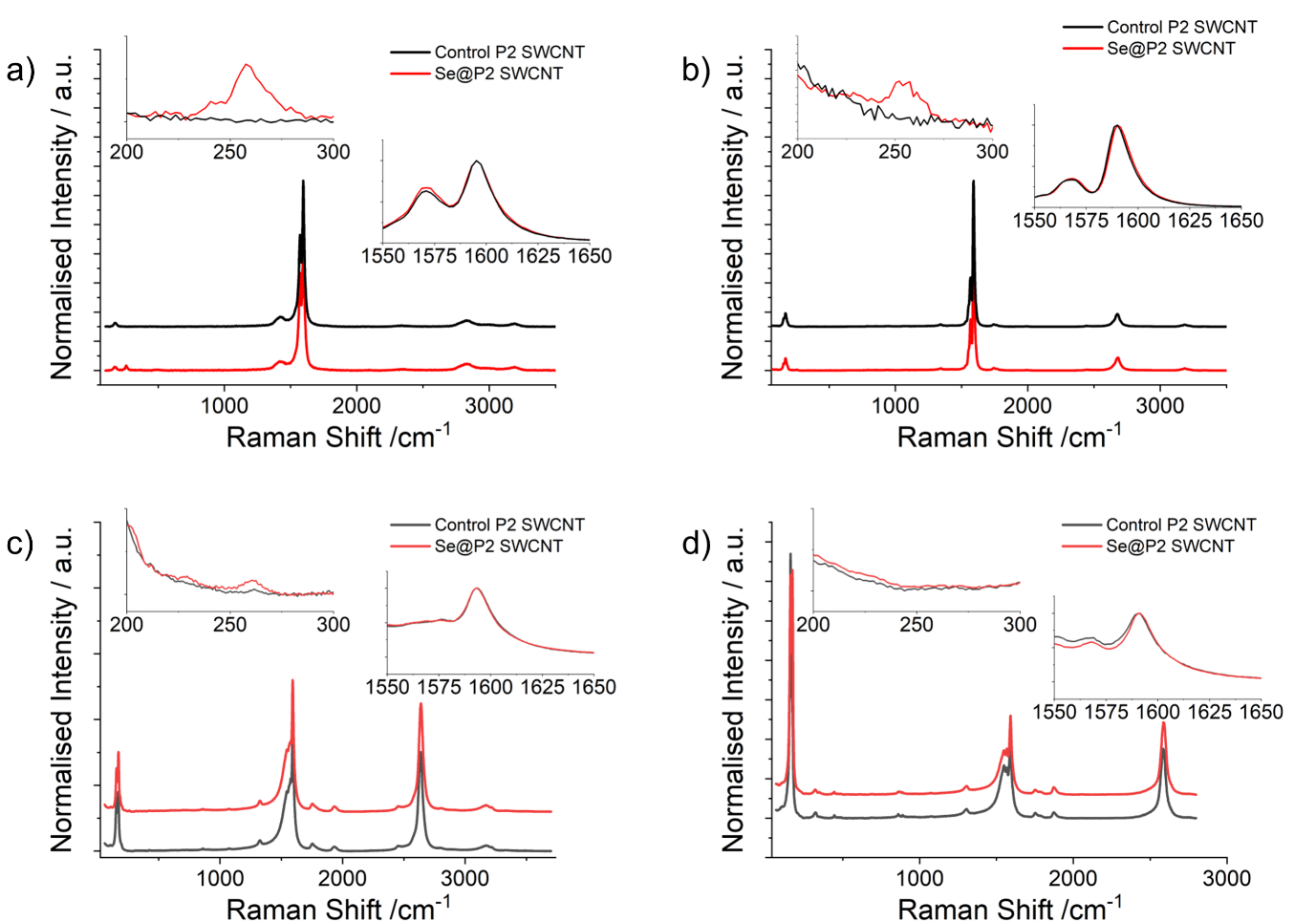


Figure S33. Resonance Raman spectroscopy analysis of Se@P2 SWCNTs at four different laser wavelengths, a) 325 nm, b) 532 nm, c) 663 and d) 785 nm. Spectra have been baseline-corrected, normalised to the intensity of the spectral maximum and offset on the y-axis for ease of visual comparison.


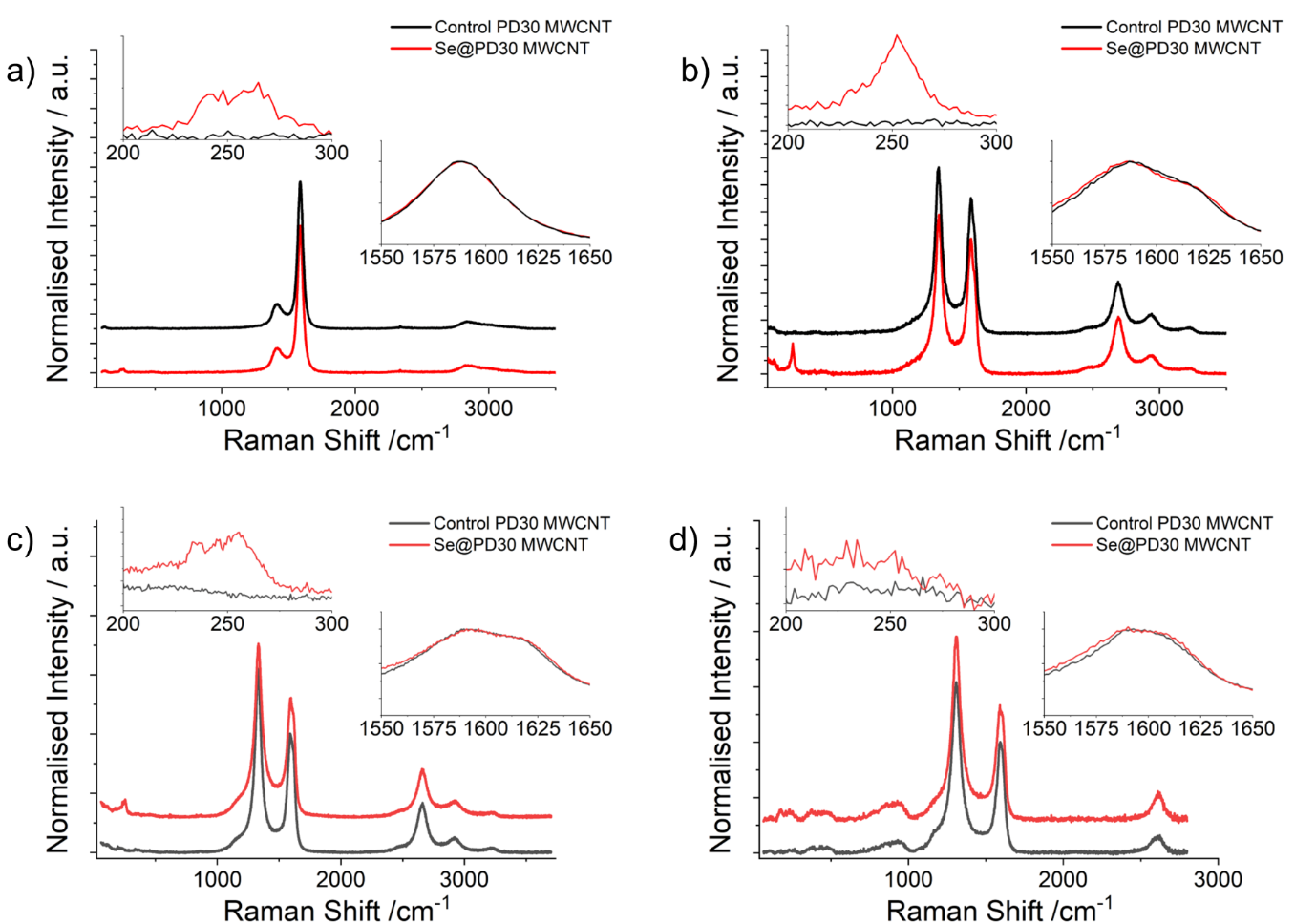


Figure S34. Resonance Raman spectroscopy analysis of Se@PD30 MWCNTs at four different laser wavelengths, a) 325 nm, b) 532 nm, c) 663 and d) 785 nm. Spectra have been baseline-corrected, normalised to the intensity of the spectral maximum and offset on the y-axis for ease of visual comparison.


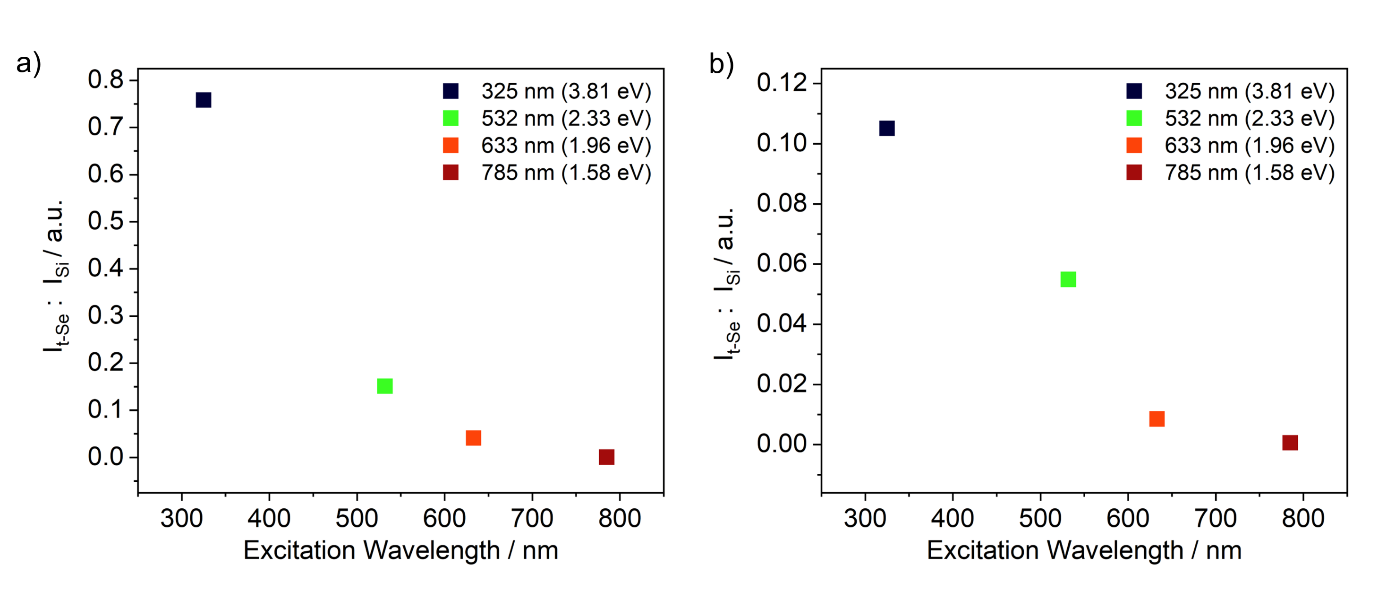


Figure S35. Summary of resonance Raman analysis of a) Se@P2 SWCNTs and b) Se@PD30 MWCNTs. By normalising the intensity of the t-Se A_1_ band to the intensity of the Si phonon mode from a reference standard collected under analogous conditions (I_t-Se_:I_Si_), the wavelength of the laser that best satisfies the resonance Raman condition for encapsulated t-Se and is thus closest in energy to the band gap can be identified. Both CNTs show a maximum I_t-Se_:I_Si_ at 325 nm, inferring an increase in the band gap from the bulk value (1.6 eV). More detailed examination of the wavelength dependence of I_t-Se_:I_Si_ indicates that the band gap of Se inside P2 SWCNTs is slightly higher than in PD30 MWCNTs due to an increased I_t-Se_:I_Si_ value when a 532 nm excitation laser is utilised, i.e., the intensity ratio maximum is likely closer to 532 nm than 325 nm for Se@PD30 MWCNT. This is commensurate with our STEM-EELS analysis, which predicts a band gap of 2.23 eV for Se inside P2 SWCNTs (0.8 nm average NW diameter) and a band gap of 2.0 eV for Se inside PD30 MWCNTs (3.2 nm average NW diameter). A full resonance window measurement would be needed to give a fully conclusive assessment of the effect of CNT diameter on the band gap of encapsulated Se in this way, and this is beyond the scope of this study and our instrumentation. Analogous analysis of Se@BNNT could not be performed due to dominant fluorescence seen when using longer wavelength excitation lasers (Figure S36). Additionally, analogous analysis of Se@HiPCO SWCNTs and Se@Nanocyl 2100 could not be performed due to overlapping of the RBM of the NTs with the t-Se A_1_ band at higher wavelengths.


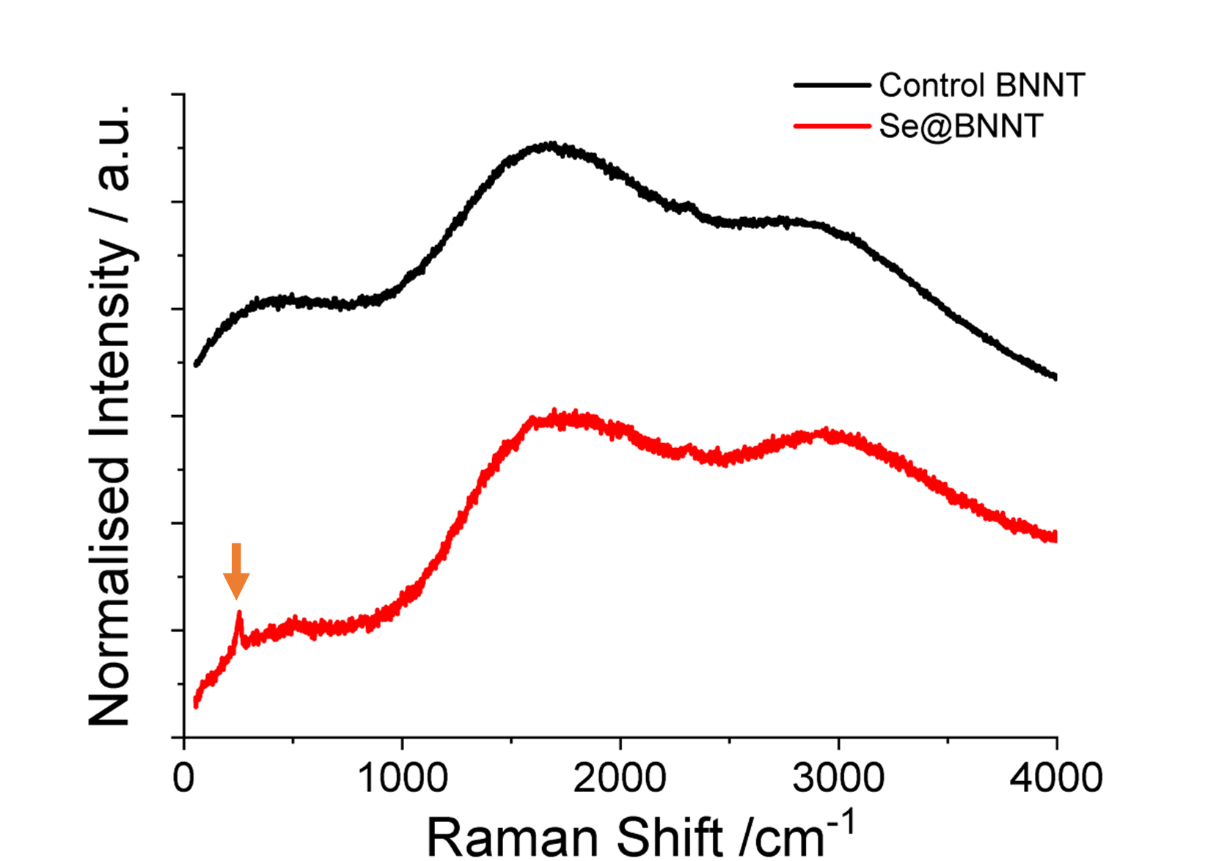


Figure S36. 532 nm Raman analysis of Se@BNNT. The orange arrow corresponds to the t-Se A_1_ stretching mode. As can be seen, the spectra are dominated by fluorescence, leading to broad emission across the entire spectral range, and likely obscuring weak Raman modes, including the expected A_1g_ tangential mode of h-BN, which should be observed at ~1370 cm^-1^. The origin of the fluorescence is likely defects in the BN wall, which can arise during the heating and cleaning steps of the Se@BNNT synthesis, possessing a broad range of emission wavelengths.^[15,16]^ Fluorescence-dominated spectra were also observed at 633 and 785 nm, precluding a multi-wavelength analysis of Se@BNNT as shown for Se@P2 SWCNT and Se@PD30 MWCNT (Figures S33 and S34).


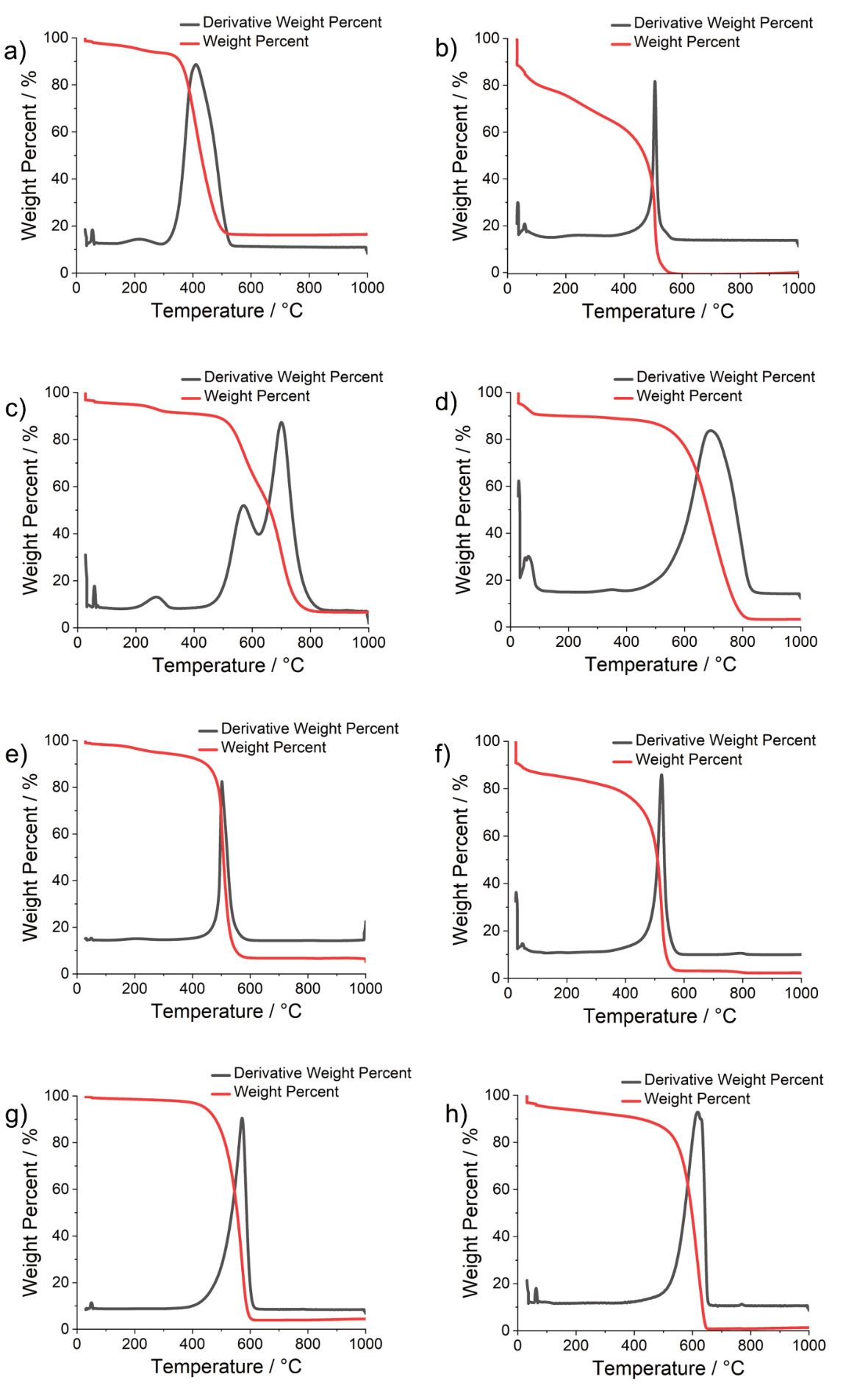


Figure S37. TGA analysis in air of pristine and opened CNTs used in this study. Black lines show the derivative weight percent, and red lines show the weight percent. a) TGA analysis of pristine HiPCO SWCNTs, b) TGA analysis of opened HiPCO SWCNTs, c) TGA analysis of P2 SWCNTs, d) TGA analysis of opened P2 SWCNTs, e) TGA analysis of pristine Nanocyl 2100, f) TGA analysis of opened Nanocyl 2100, g) TGA analysis of pristine PD30 MWCNTs, h) TGA analysis of opened PD30 MWCNTs.

Table S8. Summary of TGA analysis of the CNTs used in this study before and after CNT opening. In all cases, residual percentage weight is reduced following the nanotube opening procedure, corresponding to the removal of the metal catalyst during the acid treatment step. Additionally, in all cases except P2 SWCNTs, the position of the main derivative peak is shifted to a higher temperature upon performance of the opening procedure. This is likely due to the lesser amount of metal catalyst present following acid washing, as this catalyst is likely to also catalyse nanotube combustion. This reduction in main derivative peak position does not occur in the opened P2 SWCNTs. This is likely due to the addition of a thermal opening step, which is known to shorten nanotubes, reducing their decomposition temperature compared to pristine P2 SWCNTs.

| **Nanotube** | **Residual Weight / % (±0.01)** | **Position of main derivative peak / °C (±0.001)** |
| --- | --- | --- |
| Pristine HiPCO SWCNT | 16.4 | 409.0 |
| Opened HiPCO SWCNT | 0.0 | 506.6 |
| Pristine P2 SWCNT | 5.1 | 699.5 |
| Opened P2 SWCNT | 3.3 | 687.0 |
| Pristine Nanocyl 2100 | 6.8 | 501.9 |
| Opened Nanocyl 2100 | 2.3 | 523.3 |
| Pristine PD30 MWCNT | 4.4 | 573.1 |
| Pristine PD30 MWCNT | 1.3 | 619.7 |

References

[1] M. J. Bronikowski, P. A. Willis, D. T. Colbert, K. A. Smith, R. E. Smalley, *Journal of Vacuum Science & Technology A: Vacuum, Surfaces, and Films* **2001**, *19*, 1800.

[2] M. E. Itkis, D. E. Perea, S. Niyogi, J. Love, J. Tang, A. Yu, C. Kang, R. Jung, R. C. Haddon, *Journal of Physical Chemistry B* **2004**, *108*, 12770.

[3] M. E. Itkis, D. E. Perea, S. Niyogi, S. M. Rickard, M. A. Hamon, H. Hu, B. Zhao, R. C. Haddon, *Nano Lett* **2003**, *3*, 309.

[4] M. Lahelin, M. Annala, A. Nykänen, J. Ruokolainen, J. Seppälä, *Compos Sci Technol* **2011**, *71*, 900.

[5] F. Tian, N. C. Habel, R. Yin, S. Hirn, A. Banerjee, N. Ercal, S. Takenaka, G. Estrada, K. Kostarelos, W. Kreyling, T. Stoeger, *European Journal of Pharmaceutics and Biopharmaceutics* **2013**, *84*, 412.

[6] “Nanolab Website,” DOI 10.1088/2058-7058/13/6/27can be found under https://www.nano-lab.com/nanotubes-research-grade.html, accessed 01/07/23, **n.d.**

[7] A. Matatyaho Ya’akobi, C. J. S. Ginestra, L. R. Scammell, M. W. Smith, M. Pasquali, Y. Talmon, *J Mater Res* **2022**, *37*, 4508.

[8] J. K. Qin, P. Y. Liao, M. Si, S. Gao, G. Qiu, J. Jian, Q. Wang, S. Q. Zhang, S. Huang, A. Charnas, Y. Wang, M. J. Kim, W. Wu, X. Xu, H. Y. Wang, L. Yang, Y. Khin Yap, P. D. Ye, *Nat Electron* **2020**, *3*, 141.

[9] P. Cherin, P. Unger, *Inorg Chem* **1967**, *6*, 1589.

[10] B. L. Weare, R. W. Lodge, N. Zyk, A. Weilhard, C. L. Housley, K. Strutyński, M. Melle-Franco, A. Mateo-Alonso, A. N. Khlobystov, *Nanoscale* **2021**, *13*, 6834.

[11] J. Kotakoski, C. H. Jin, O. Lehtinen, K. Suenaga, A. V. Krasheninnikov, *Phys Rev B Condens Matter Mater Phys* **2010**, *82*, 1.

[12] A. Celik-Aktas, J. F. Stubbins, J. M. Zuo, *J Appl Phys* **2007**, *102*, 024310.

[13] G. Cheng, S. Yao, X. Sang, B. Hao, D. Zhang, Y. K. Yap, Y. Zhu, *Small* **2016**, *12*, 818.

[14] J. C. Meyer, F. Eder, S. Kurasch, V. Skakalova, J. Kotakoski, H. J. Park, S. Roth, A. Chuvilin, S. Eyhusen, G. Benner, A. V. Krasheninnikov, U. Kaiser, *Phys Rev Lett* **2012**, *108*, 1.

[15] T. T. Tran, K. Bray, M. J. Ford, M. Toth, I. Aharonovich, *Nat Nanotechnol* **2016**, *11*, 37.

[16] S. X. Li, T. Ichihara, H. Park, G. He, D. Kozawa, Y. Wen, V. B. Koman, Y. Zeng, M. Kuehne, Z. Yuan, S. Faucher, J. H. Warner, M. S. Strano, *Commun Mater* **2023**, *4*, 1.
